# Genomic Characterization of *Candida* spp. Highlights a Persistent, Azole-Resistant *C. parapsilosis* Clone Circulating in a Tertiary Care Hospital During the First COVID-19 Wave

**DOI:** 10.1007/s11046-026-01070-9

**Published:** 2026-03-16

**Authors:** Michela Vumbaca, Gherard Batisti Biffignandi, Caterina Cavanna, Greta Bellinzona, Marta Corbella, Irene Mileto, Johanna Rhodes, Jukka Corander, Fausto Baldanti, Davide Sassera

**Affiliations:** 1 Department of Biology and Biotechnology, Università di Pavia, Pavia, Italy; 2 Microbiology and Virology Department, Fondazione IRCCS Policlinico San Matteo, Pavia, Italy; 3 Regional Centre for Infectious Diseases, CEREMI, Lombardy Region, Italy; 4 Department of Clinical, Surgical, Diagnostic and Pediatric Sciences, Università di Pavia, Pavia, Italy; 5 Department of Medical Microbiology, Radboudumc, Nijmegen, The Netherlands; 6 MRC GIDA, Imperial College London, London, UK; 7 Department of Biostatistics, University of Oslo, Oslo, Norway; 8 Department of Mathematics and Statistics, University of Helsinki, Helsinki, Finland; 9 Parasites and Microbes, Wellcome Sanger Institute, Hinxton, UK; 10 Fondazione IRCCS Policlinico San Matteo, Pavia, Italy

**Keywords:** Candidemia, Azole resistance, *Candida albicans*, *Candida parapsilosis*, COVID-19, ERG11

## Abstract

Yeasts belonging to the *Candida* genus typically reside on the mucosal surface and within the respiratory and gastrointestinal tract as commensals. Under conditions of host vulnerability, they can act as opportunistic pathogens, leading to various forms of candidiasis, including candidemia. Such infections can be particularly problematic when caused by isolates that exhibit resistance to antifungal drugs, which are becoming more prevalent in many regions. One hundred and seven samples of *Candida* spp. were isolated from patients with candidemia in the hospital San Matteo in Pavia (Italy) over a period of 6 years, from 2015 to the first COVID wave in spring 2020. In order to understand the epidemiology of *Candida* infections in this hospital setting, the isolates were whole-genome sequenced, confirming that most belonged to *C. parapsilosis* and *C. albicans*. Comparative genomics revealed that isolates of *C. albicans* were genomically diverse, indicative of repeated introductions in the hospital from the community. *C. parapsilosis* isolates on the other hand belonged to two groups of highly similar isolates, representing strains capable of long-term persistence in the hospital. All isolates of the main persistent group were resistant to fluconazoleresulting from the Y132F substitution in *ERG11* and the N455D substitution in *UPC2*, while presenting variable levels of resistance to voriconazole and itraconazole. Interestingly, with the exception of the single isolate susceptible to both voriconazole and itraconazole, all the 61 isolates presented one unreported missense mutation in *MRR1* (S1907C).

**Supplementary Information:**

The online version contains supplementary material available at 10.1007/s11046-026-01070-9.

## Introduction

*Candida* species are the leading cause of fungal infections in hospital settings [1] typically colonizing the skin and mucosal surfaces as commensal pathogens. However, in immunocompromised individuals they are often associated with more serious infections referred to as candidiasis, which can occur as cutaneous, mucosal, or systemic infections, and also lead to systemic and bloodstream infections (also known as candidemia) [2].

Over the past few decades the most prevalent species of *Candida* causing nosocomial infections worldwide has been *Candida albicans*, although its incidence is decreasing with a concomitant increase of non-*albicans* species such as *Candida auris*, *Nakaseomyces glabratus*, *Candida parapsilosis*, *Candida tropicalis* and *Candida krusei* [3–5]. *C. parapsilosis* has become the most common non-*albicans* species isolated in southern Europe, some regions of Asia and Latin America [6], together with *C. norvegensis, C. fermentati,* and *C. kefyr *[7]*.* While *C. albicans* isolates remain susceptible to azole antifungal therapy, drug resistance has been increasing in non-*albicans* species, posing challenges for treatment and infection control measures. Among the reported instances of resistance, azole resistance in *C. parapsilosis* [8] has been frequently reported associated to outbreaks, especially during the COVID-19 pandemic, due to forced relaxation of infection control procedures and change in preventive protocols [8, 9]. Indeed, the rapid spread of COVID-19 in 2020 led to a significant increase in the number of hospitalized patients with a consequent breakdown in hospital control measures. As a result, along with the use of contaminated devices and patients' vulnerability, the number of bacterial and fungal infections, including *Candida,* increased*.* It must be noted that this trend has not reverted after the end of the pandemic, possibly indicating that these azole-resistant strains are here to stay [4].

Azole resistance is a complex phenomenon influenced by gene copy number variation [10, 11], changes in gene expression levels and/or amino acid substitutions in azole targets [12]. The primary mechanism responsible for azole resistance in *C. parapsilosis* involves mutations in the azole target gene lanosterol 14-alpha demethylase (*ERG11*), belonging to the pathway of ergosterol biosynthesis, which is necessary for fungal growth. Multiple amino acid substitutions in this gene have been reported to reduce the affinity for the drug, conferring azole resistance. Y132F in *ERG11* is the most common substitution detected in fluconazole-resistant strains, followed by others such as G458S, K128N, K143R or D412N [13–15]. Instances of these substitutions in *ERG11* have been reported worldwide in *C. parapsilosis* isolates non-susceptible to fluconazole [15]. Less common resistance mechanisms involve mutations in other genes, including the transcription factors *UPC2*, *TAC1* and *MRR1*. These respectively control transcription of *ERG11* [16], of the ATP-binding cassette transporter (composed of two subunits, *CDR1* and *CDR2*) [17] and of the facilitator superfamily transporter MDR1 [18]. Gain-of-function mutations in *TAC1* and *MRR1* transcription factors lead to an overexpression of these two efflux pumps, enhancing the drug efflux [12, 19].

In this study we conducted a retrospective epidemiological analysis on *Candida* spp. isolated from patients affected by candidemia in the hospital IRCCS Policlinico San Matteo (Pavia, Italy) over a six-year period. Different species of *Candida* were identified, with most infections caused by *C. albicans* and *C. parapsilosis*, and an increase of the latter during the first wave of the COVID-19 pandemic. Evolutionary and epidemiological relationships between samples were inferred through genomic analysis, highlighting the presence of a persistent, azole-resistant *C. parapsilosis* strain circulating within the hospital. Isolates were characterized for their antifungal resistance and genotyping analyses were conducted, detecting the presence of both common and novel non-synonymous mutations in multiple genes associated with azole resistance.

## Materials and Methods

### Sample Collection and Species Identification

Blood cultures from patients with suspected sepsis were processed using BD-Bactec FX (Becton Dickinson, Franklin Lakes, NJ, USA) employing BD BACTEC Plus Aerobic/F and Plus Anaerobic/F culture vials. When Gram staining from a positive blood culture indicated the presence of yeast, multiplex PCR (BioFire® FilmArray®) was conducted, allowing to obtain a preliminary classification, as this method allows to identify isolates belonging to five *Candida* species (*C. albicans, N. glabratus, C. norvegensis, C. parapsilosis, C. rugosa*). *Candida* species were then grown on conventional culture and the identification of the isolates was performed using Matrix-assisted laser desorption/ionization time of flight mass spectrometry (MALDI-TOF-MS, Bruker Daltonics, Bremen, Germany) using the software Bruker Biotyper 3.1.

The 107 isolates included in the analysis represent all candidemias that occurred in the San Matteo Hospital in Pavia during the study period (January 2015 to April 2020), with the exception of the period between 2015 and 2017, when only a portion of samples isolated from affected patients could be recovered (the total number of candidemia in this period is unknown). Our analyses include 107 isolates of *Candida* spp*.* isolated from 106 patients diagnosed with candidemia admitted to multiple wards, with a single case of two isolates collected from a single patient.

### Antifungal Susceptibility Test

Antifungal susceptibility testing was performed using Sensititre YeastOne (SYO, Thermo Scientific Trek Diagnostic System, East Grinstead, UK). Antifungals tested were those included in the SYO panel, namely amphotericin B, anidulafungin, caspofungin, micafungin, fluconazole, itraconazole, posaconazole and voriconazole. Minimal inhibitory concentration (MIC) values obtained with SYO were interpreted according to Clinical Laboratory and Standards Institute (CLSI) species-specific breakpoints [20] or, in case of lack of these breakpoints, according to the SYO epidemiological cutoff values (ECV). Specifically, as posaconazole is not included in the CLSI guidelines, we used EUCAST v.7.0 [21] to assess resistance to this drug.

### DNA Extraction, Genome Sequencing and Genome-Based Species Identification

For extraction of genomic DNA, cells from single colonies of each candidemia isolate were picked and grown overnight at 30 °C in four ml cultures of YPD broth (1% [wt/vol] Difco yeast extract, 2% [wt/vol] Bacto peptone, 2% [wt/vol] dextrose) with shaking. DNA was then extracted with the Qiagen Genomic-tip 100/G using the Qiagen genomic buffer set (Qiagen, Hilden, Germany), following the manufacturer’s yeast protocol. Cell wall digestion was accomplished with lyticase (catalog number L2524; Sigma-Aldrich St. Louis, Missouri, USA). Library preparation and 2 × 150 bp paired-end sequencing was performed using the Illumina NovaSeq platform.

Raw sequencing reads were quality checked and trimmed to remove low quality bases and adapter sequence using FastP v.0.23.2 [22]. fastANI v.1.33 was used for genome-based species identification [23]. One clinical isolate of *Candida parapsilosis* (35763_2_2) was chosen for additional long read sequencing using the MinION sequencer (Oxford Nanopore Technology, Oxford, UK). The selection of a sample from the persistent strain for long-read sequencing was based on a comparative evaluation of multiple samples. The selection criteria encompassed the number of short reads generated, the quality of a preliminary genome assembly, and the possibility to revive the isolate in culture.

### Genome Mapping, Assembly and Phylogeny

*C. albicans* and *C. parapsilosis* reads of each isolates were mapped to their reference genomes, ASM18296v3 and ASM3628897v1 respectively, with Bowtie2 v.2.5.4 [24] with default settings and converted to sorted BAM format using Picard SortSam v.3.1.1 (https://broadinstitute.github.io/picard/). PCR duplicates reads were identified and marked through Picard MarkDuplicates. Genome Analysis Toolkit (GATK) v.4.4.2 HaplotypeCaller was employed for variant calling (single nucleotide polymorphism (SNPs) and indels) using default settings, while specifying samples diploidy and enabling the option -ERC GVCF [25]. The GVCFs were subsequently imported into a GATK GenomicsDB to merge GVCFs from multiple samples into a combined VCF (one for each *Candida* species) with the GenotypeGVCFs GATK function. Variants from the merged VCF were filtered with the VariantFiltration GATK option, to select high-confidence variants using the following parameters: DP <  = 20, QD < 2.0, MQ < 40.0, FS > 60.0, MQRankSum < − 12.5 and ReadPosRankSum < − 8.0. Heterozygous positions were identified using the same GATK VariantFiltration function by applying a genotype-level filter with the expression isHet == 1, which allowed us to flag genotypes in heterozygosity across our isolates, which were then included in our analyses. Only SNPs were retained through GATK SelectVariants with –select-type-to-include SNP option. Samples that had genome quality (–minGenomeQuality) lower than 50 and sample depth (–minSampleDepth) lower than 30 were removed using VcfFilter v.0.2 (https://github.com/biopet/vcffilter). The multi-samples VCF resulting from the mapping procedure was converted into a FASTA alignment using ‘vcf2phylip’ (Ortiz et al. 2019) and invariant sites were removed through snp-sites v.2.5.1 [26]. Pairwise SNPs distances between isolates were calculated and visualized as heatmaps using “Snpbreaker.R” script from P-DOR pipeline [27]. Maximum-likelihood phylogenetic trees were inferred with IQTREE v.2.0.7 [28] using the MFP-GTR model and 1000 bootstrap replicates. To place the isolates from San Matteo Hospital within a broader global context, phylogenies of both *C. albicans* and *C. parapsilosis* were performed including additional publicly available genomes representing the geographical diversity of the two species. Strains metadata retrieved from public databases are listed in Table S2.

Illumina reads from isolates belonging to the four less common species *N. glabratus*, *D. rugosa*, *C. norvegensis* and *C. orthopsilosis* were de novo assembled to provide novel Italian references for further studies, since only a limited number of genomes were available on public databases (searching NCBI on September 2024 we found: *N. glabratus*: 69; *C. norvegensis*: 3; *D. rugosa*: 2; *C. orthopsilosis*: 7). They were subjected to genome assembly using Unicycler v.0.5 [29] with default parameters. The reads from the *C. parapsilosis* isolate 35763_2_2, sequenced with both Illumina and Nanopore technology were assembled into an hybrid assembly using Unicycler v.0.5 [29] with the –linear_seqs 8 option, representing the predicted presence of eight linear chromosomes in the *C. parapsilosis* reference assembly ASM3628897v1. Completeness and quality of the assembly were evaluated with BUSCO v5.8.0 [30].

### Genomic Characterization of *C. parapsilosis* Isolates

Genomic variants of *C. parapsilosis* were annotated with snpEff v.5.2a [31] from the VCF files, using a custom database generated from the ASM3628897v1 reference genome after annotation with Augustus [32] using the yeast mitochondrial codon tables. SnpEff was used to identify mutations present in the genes previously reported to be involved in azole resistance: *ERG11*, *FKS1*, *MRR1*, *ERG3*, *TAC1*, *UPC2* and *NDT80*. Copy number variations (CNVs) were determined from BAM files using Delly v.1.2.6. CNV calling and the output files were plotted using rd.R script [33].

### Statistical Analysis

Univariate analysis was used to evaluate the relationship between *Candida* infections, patient outcome, length of hospital stay and the number of days from hospital admission to onset of infection (this latest inferred from the date of clinical sampling that leads to pathogen isolation). Categorical variables were compared using the Fisher exact test, followed by calculation of standardized residuals to explore specific associations. Residuals with values > 1.96 or < 1.96 were considered statistically significant deviations from the expected frequency. Continuous variables were compared using the Kruskal–Wallis test for multiple-group comparison. Pairwise comparisons were conducted with the Wilcoxon rank-sum test and Benjamini–Hochberg correction for multiple testing when there was a significant difference. All statistical tests were two-tailed and *p* values < 0.05 were considered statistically significant. Analyses were performed using R (version 4.2.2).

## Results

### *Candida* spp. Distribution Over the Study Period

Over the six years period of the study (between January 2015 to April 2020), a total of 107 isolates of *Candida* spp*.* were obtained and characterized from 106 patients diagnosed with candidemia (median age 64; range 19–89). Species identification was initially performed through MALDI-TOF, which identified five different species, namely *C. albicans* (*n* = 18), *Nakaseomyces glabratus* (formerly known as *C. glabrata*) (*n* = 3), *C. norvegensis* (*n* = 1), *C. parapsilosis* (*n* = 84) and *Diutina rugosa* (formerly known as *C. rugosa*) (*n* = 1). The metadata, including information on the species identified, the date and ward of sample isolation, as well as MIC values for each antifungal drug tested are provided in Table S1.

All isolates were subjected to Illumina whole genome sequencing, obtaining an average of 40,750,143 reads per sample. Genome-based species identification was then performed, detecting three cases of discordance with the MALDI-TOF results. Specifically, three *C. parapsilosis* were re-classified as *C. orthopsilosis*. The misidentification of *C. orthopsilosis* as *C. parapsilosis* by MALDI-TOF is likely due to the high similarity between these closely related species. Other studies reported inconsistencies in species identification within the *C. parapsilosis* complex between MALDI-TOF results and molecular methods, suggesting that the accuracy of MALDI-TOF identification could be improved or complemented by genomic approaches [34, 35]. Genome-based species identification was used for subsequent analyses.

A non-parametric test was used to analyze the time from onset of infection and the length of hospitalization for isolated *Candida* species. The Kruskal–Wallis test did not reveal statistically significant differences among isolated *Candida* species for neither onset of infection (χ^2^ = 10.023 and *p* value = 0.074) nor length of hospitalization (χ^2^ = 3.2217 and *p* value = 0.6658). These results indicate that no species was significantly linked to a longer stay or a delayed time to onset after patient admission.

**Fig. 1:**
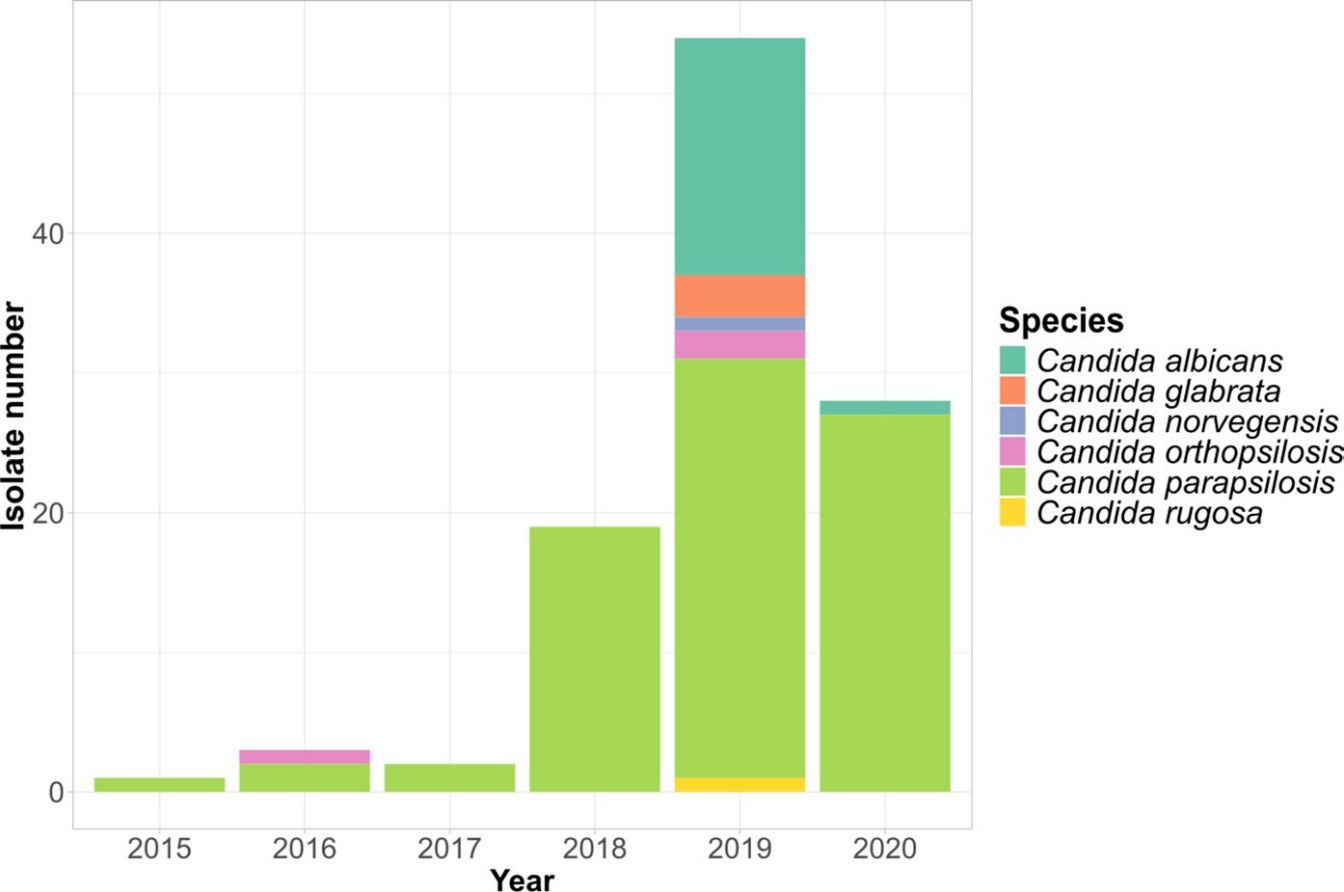
Barplot of the 107 *Candida *isolates from the hospital San Matteo in Pavia over the six years period, colour coded by species. The last bar shows the isolates collected untile 14 April 2020, when the sample collection for this study ended

Among the identified species, *C. parapsilosis* and *C. albicans* were the most prevalent (n > 17), with a stable trend over the years (Fig. 1). Genomes of these species were thus subjected to ad-hoc analyses below.

Although no further epidemiological investigation was carried out for the less common *Candida* species (n < 4), we performed genome assembly and reported the corresponding sequencing results, as the number of available genomes belonging to these species in public databases is currently limited. Draft genome assemblies were obtained, with the total assembly length ranging from 12.16 to 12.23 Mb for *N. glabratus*, from 13.57 to 15.79 Mb for *C. orthopsilosis*, of 13.58 Mb for *D. rugosa* and of 8.03 Mb for *C. norvegensis*.

### *Candida albicans* Isolates are not Epidemiologically Linked

**Fig. 2:**
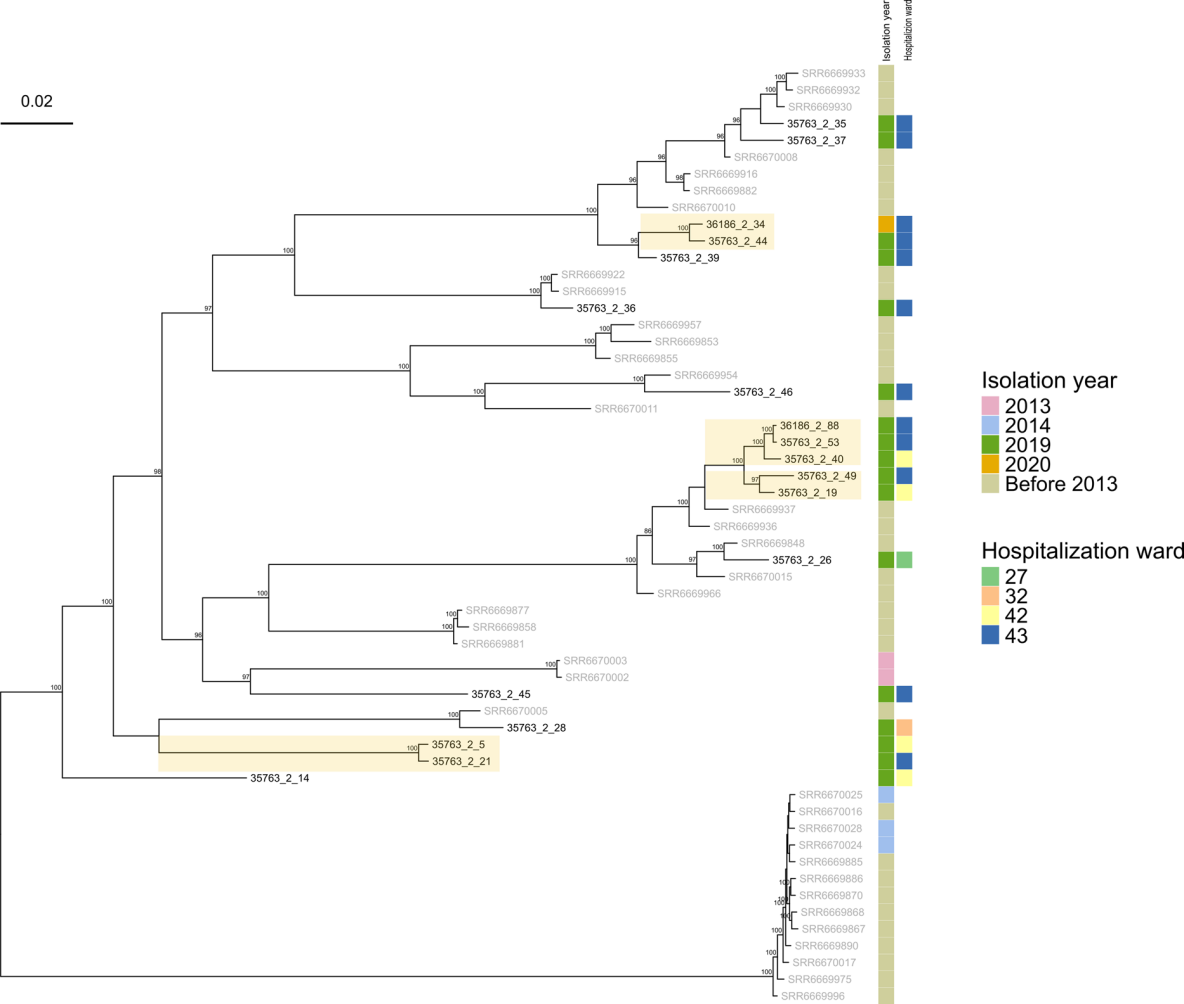
*Candida albicans* phylogeny with global contextualisation (see **Table S2** for the complete dataset), obtained from a whole genome SNP alignment of sequencing reads mapped to reference genome ASM18296v3, using IqTREE v.2.0.7. Isolate labels from San Matteo Hospital are displayed in black, while labels for publicly available genomes are shown in gray. Phylogeny is midpoint-rooted. Bootstrap values above 80 are reported on the corresponding node. For the novel isolates, year and hospital ward of isolation are indicated. The scale bar represents the branch length of the phylogenetic tree measuring the evolutionary distance between isolates

*C. albican*s was the second most prevalent species in our dataset, for a total of 18 isolates*.* In this case, all isolates except one were collected in 2019, from different wards in the San Matteo Hospital (Table S1). To evaluate the potential epidemiological connections we constructed a dataset including additional 38 publicly available genomes from NCBI (see Methods section). Reference-based genome mapping allowed us to obtain a set of 41,403 whole-genome SNPs, which was then used to generate a maximum likelihood tree (Fig. 2).

The resulting phylogeny revealed the local *C. albicans* isolates are scattered throughout the tree and do not cluster in large monophyletic groups, indicating that these are independent cases that have been introduced in the hospital separately. However, we observed four small highly supported monophyletic clades (bootstrap 97 or above) composed only of genomes from the San Matteo hospital (Fig. 2). The SNP distances within these clades are relatively high, from a minimum of 146 SNPs to a maximum of 424 SNPs. Isolates 36186_2_34 and 35763_2_44 are separated by 212 SNPs, isolates 36186_2_88, 35763_2_53 and 35763_2_40 exhibit an average SNP distance of 249, while isolates 35763_2_49-35763_2_19 and isolates 35763_2_5-35763_2_21 are separated by 424 SNPs and 339 SNPs respectively. These distances suggest that the isolates likely represent related lineages rather than direct epidemiological links (Fig. S1).

### *Candida parapsilosis* phylogenomics Reveals Introduction of a Persistent Clone Resulting in a Nosocomial Outbreak

**Fig. 3:**
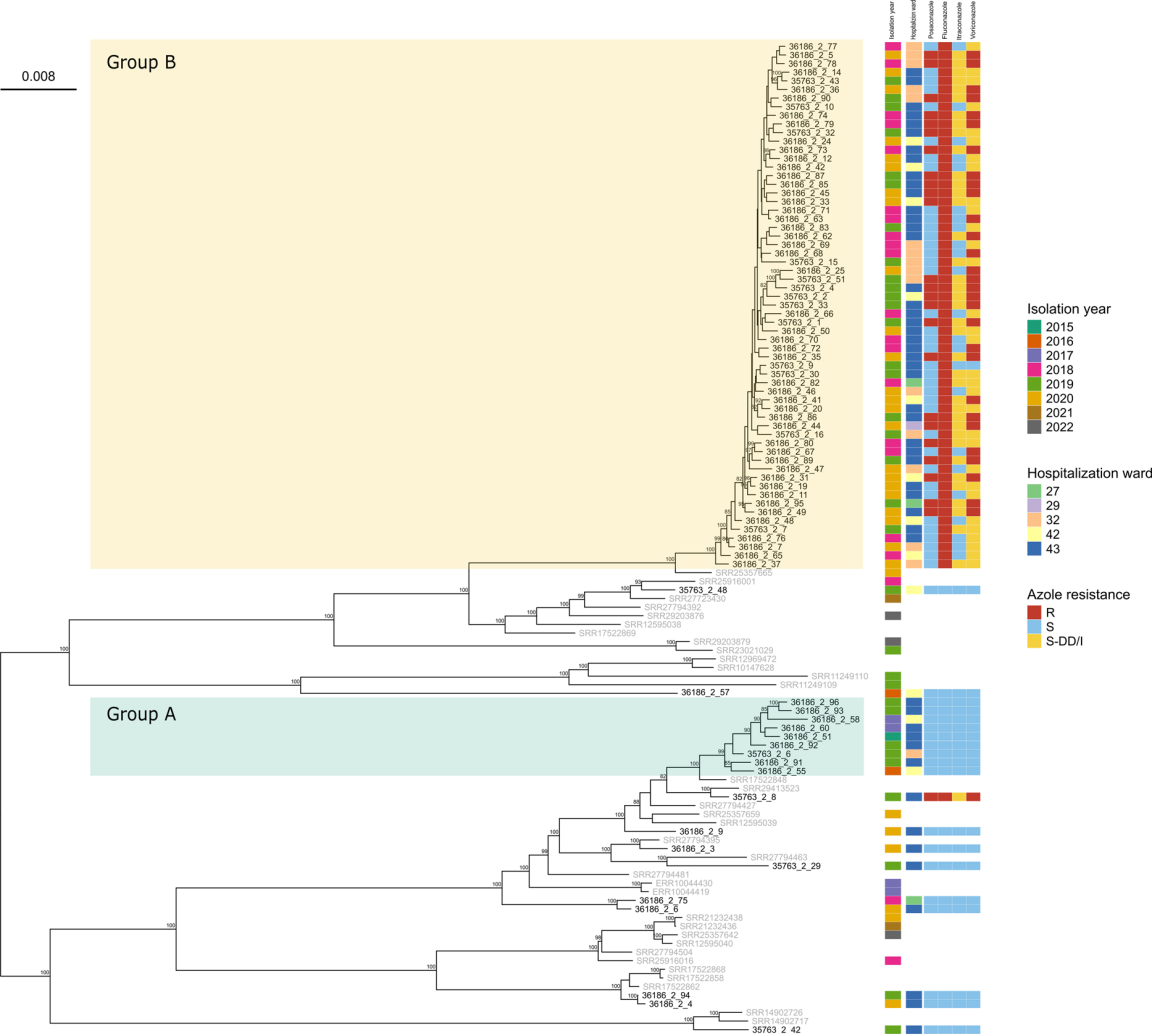
Whole genome SNP maximum-likelihood phylogeny of *Candida parapsilosis* isolates with a global context (Table S2). Isolates from San Matteo Hospital are labeled in black, while labels for publicly available genomes are shown in gray. Phylogeny is midpoint rooted. Bootstrap values above 80 are reported on the corresponding node. For novel isolates, year, hospital ward of isolation and profiles of resistance to azoles are indicated. The scale bar represents the branch length of the phylogenetic tree measuring the evolutionary distance between isolates

A phylogenomic approach was applied also to the 81 *C. parapsilosis* genomes obtained from samples collected from different wards of the hospital between 2015 and 2020, with the addition of 34 publicly available genomes from other studies (Table S2**)** [8, 36–47], to represent global diversity and to provide broader comparative context (see Methods section) (Fig. 3).

The phylogeny shows that 70 out of 81 genomes belong to two fully supported monophyletic clades (bootstrap 100) of novel isolates. The first (Fig. 3**, **Group A) consists of nine isolates collected in 2019 and 2020 and the second (Fig. 3, Group B) consists of 61 isolates collected from 2018 to 2020, with SNPs distance < 70 and < 40 respectively. The remaining 11 novel isolates are scattered across the global phylogeny without clear clustering (Fig. 3). This pattern suggests that these last 11 isolates represent multiple sporadic introductions into the hospital over time. The pairwise SNP distances among all *C. parapsilosis* genomes of the dataset are represented via a heatmap in Fig. S2.

In order to investigate the clinical impact of the different *C. parapsilosis* groups, we evaluated whether belonging to Group A, Group B or being a sporadic isolate influenced time from admission to onset of infection and length of stay. The Kruskal–Wallis test highlighted significant differences across the groups for both the time to infection (χ^2^ = 21.053, *p* < 0.001) and the length of stay (χ^2^ = 9.6362, *p* = 0.008). The Kruskal–Wallis test was followed by the Wilcoxon Rank test which revealed significant association between belonging to Group B and longer intervals from admission to onset compared to both Group A and sporadic cases (*p* = 0.001), while no difference was found between Group A and sporadic cases. Similarly Group B exhibited significantly longer lengths of hospitalization compared to Group A and to sporadic cases (*p* = 0.032), while no significant difference was found between Group A and sporadic cases. See Supplementary Table S3.1 for detailed descriptive statistics, the distribution of these values is also visualized in the boxplot in Fig. S3.

Additional statistical analyses were performed to evaluate associations between the different groups and patient outcome (Deceased, Discharged, Transferred). Although the association between groups and outcomes was not statistically significant (Fisher’s exact test, *p* = 0.104), a clinical pattern was identified by the analysis of standard residuals and relative frequencies. Group B emerged to be negatively associated with hospital discharge (22.9%; std res = − 2.29), as opposed to sporadic isolates which have a higher frequency of discharge (63.6%; std res =  + 2.65). See Supplementary Table S3.2 for the complete distributional analysis.

Due to the impact of the main persistent strain (Group B) and considering its peculiar pattern of drug resistance (all isolates resistant to fluconazole, multiple isolates resistant to other azoles, see Fig. 3), we selected one isolate (see Materials and Methods for the parameters) for long read sequencing with Oxford Nanopore technology, allowing us to obtain a chromosome-level assembly (BUSCO score: C:97.3% [S:97.0%, D:0.3%], F:0.3%, M:2.4%). This novel genome is consistent both for genome length (13.0–13.1 Mbp) and number of genes (5927–5958) with the ASM3628897v1 reference genome of this species.

### Antifungal Susceptibility Profiles and MIC Distributions Across Isolates

We determined the antifungal resistance and susceptibility profiles for all isolates via MIC values and CLSI breakpoints (Table S1**)**. None of the *C. albicans* isolates were found to exhibit resistance to any of the antifungal drugs evaluated, consistent with the literature, which reports very rare occurrences of resistance in this species [48]. The same pattern was observed in *D. rugosa* and *C. orthopsilosis* isolates, although the lack of well-established clinical breakpoints for these rare species limits interpretability of these results. Conversely, *N. glabratus* isolates exhibited reduced susceptibility to itraconazole and fluconazole (classified as S-DD) and the single *C. norvegensis* isolate showed resistance to 5-flucytosine (MIC: 32 μg/mL), fluconazole (MIC: 128 μg/mL) and voriconazole (MIC: 1 μg/mL).

**Fig. 4:**
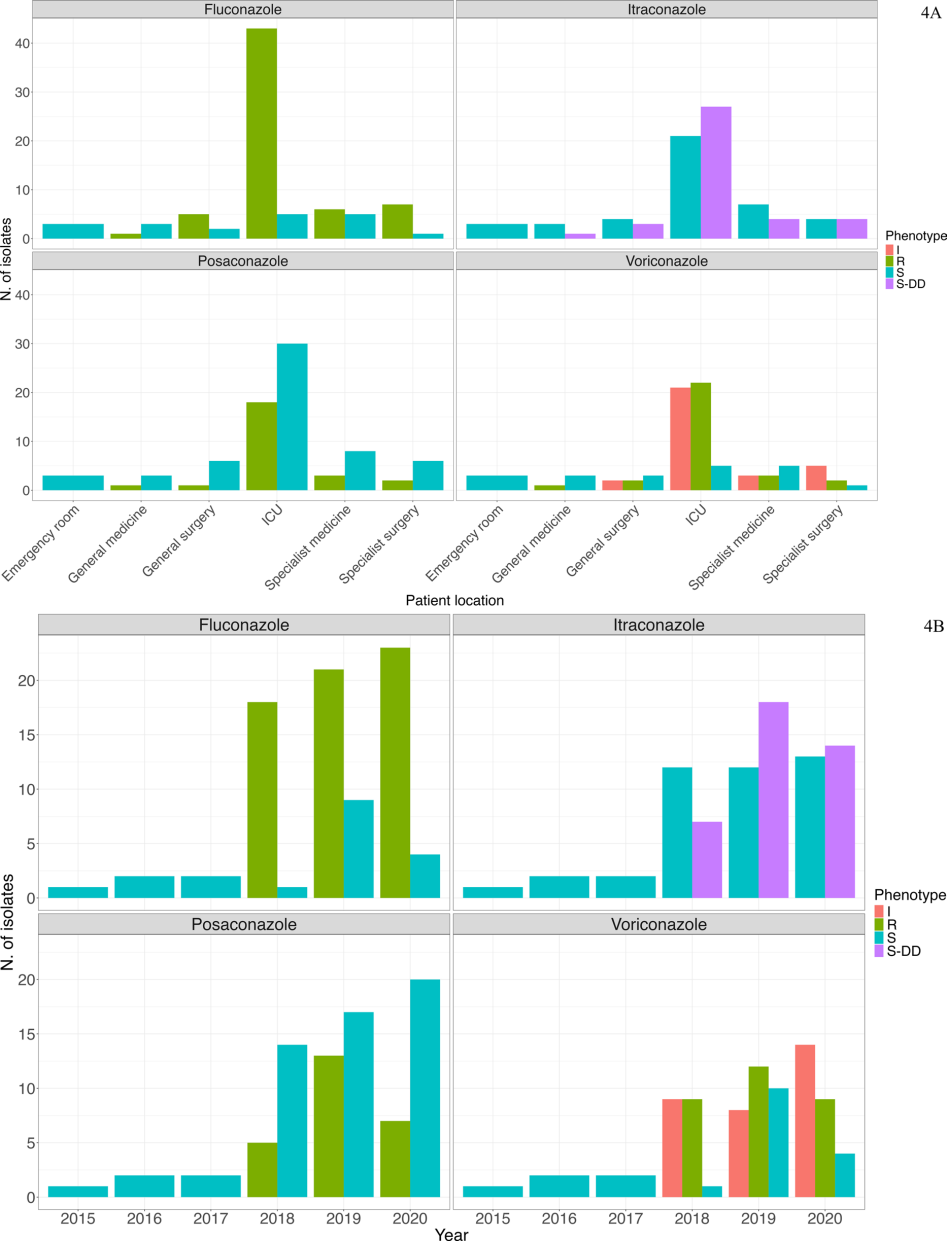
Susceptibility profile of *C. parapsilosis* isolates to azoles tested. **A** per patient location, **B** per year

Whereas the other species considered in the study showed more uniform resistance patterns, a broader range of phenotypes was observed in the case of *C. parapsilosis*, whose isolates exhibited the most diverse resistance profile. The 61 isolates belonging to the main persistent clone (Group B) showed variable profiles of resistance to azoles. All 61 isolates resulted resistant to fluconazole with MICs ranging from 16 to 256 μg/mL. MICs to itraconazole, posaconazole and voriconazole were more variable, resulting in all combinations of susceptible, intermediate or resistant phenotypes. The remaining 20 *C. parapsilosis* isolates*,* the ones not belonging to the main persistent clade, resulted susceptible to all tested azoles, with the single exception of 35763_2_8 which displayed high MICs to fluconazole, posaconazole and voriconazole and intermediate MIC to itraconazole (256 μg/mL for fluconazole, 0.125 μg/mL for posaconazole, 0.1 μg/mL for voriconazole and 0.25 μg/mL to itraconazole). All 81 isolates resulted to be susceptible to echinocandins (micafungin, caspofungin and anidulafungin, MIC: 0.25–2 μg/mL), amphotericin B (MICs ≤ 1 μg/mL) and 5-flucytosine (MICs ≤ 2 μg/mL) with the single exception of isolate 36186_2_91 which resulted to have intermediate MIC to anidulafungin (MIC: 4 μg/mL) and micafungin (MIC: 4 μg/mL). Our data show that resistance to azoles emerged in 2018 and spread primarily in the ICU (Fig. 4a). This trend reflects the emergence of the persistent clone; indeed, isolates from 2015 to 2017 were fully susceptible to the four tested azoles. From 2018 onwards most isolates maintained a susceptible phenotype for posaconazole, while for itraconazole and voriconazole the distribution was more balanced, with susceptible isolates identified in similar numbers to those classified as resistant, susceptible dose-dependent (S-DD) and intermediate (Fig. 4b).

### *Candida parapsilosis* Genomic Determinants of Resistance to Azoles

Considering their pattern of resistance to azoles, all 81 isolates of *C. parapsilosis* were analyzed to try to detect the genetic determinants of resistance. The presence of non-synonymous SNPs (nsSNP) were investigated in specific genes: *ERG11*, *FKS1*, *ERG3*, *MRR1*, *UPC2*, *TAC1* and *NDT80*, which have previously been reported to be involved in resistance [12, 16, 49, 50]. All 61 fluconazole-resistant isolates belonging to the persistent clone harbor the well-known Y132F substitution in *ERG11*, caused by the 395A>T DNA transversion, present in homozygosity in all these isolates, while absent in all susceptible isolates. None of the other common mutations in *ERG11*, G458S, K128N, and K143R [51, 52] were detected in our dataset.

Mutations in *UPC2*, gene that encodes for a transcription factor that regulates the ergosterol biosynthesis by modulating the *ERG11* activity, can lead to an increased production of ergosterol, allowing the organism to evade the effects of azole antifungals [53]. All *C. parapsilosis* isolates belonging to the main persistent group (*n* = 61) harbor the heterozygous nsSNP resulting in the amino acid substitution N455D.

We also detected a previously unreported heterozygous nsSNP substitution in *MRR1*, a zinc cluster transcription factor which influences the expression of Cdr1 and Mdr1 efflux pumps, with the result of an increased secretion of azoles from the cell [18]. The novel nsSNP causes a serine to cysteine (S1907C) amino acid substitution, and is present in all 61 persistent group isolates, with the single exception of 35763_2_9. Finally, analyses of *ERG3*, *TAC1* and *NDT80*, also reported to be involved in development of azole resistance [16, 19, 54], showed no mutation in any isolate.

All isolates displayed susceptibility to echinocandins, with the exception of 36186_2_91, which displayed intermediate MICs for anidulafungin and micafungin. We therefore investigated the presence of mutations in *FKS1*, as catalytic subunits of Fks1 are the target of echinocandins drugs [55]. Analysis of *FKS1* coding regions showed a single mutation in isolate 35763_2_7, which harbored a glycine to serine substitution (G268S) within Fks1. This mutation has, to our knowledge, never been reported in the literature.

In addition to substitutions in specific genes, azole resistance in *C. parapsilosis* as in other *Candida* species can be driven by chromosomes aneuploidy [11, 56], through which organisms can adapt to environmental changes, providing a selective advantage during clinical treatment [57]. Copy number variations (CNVs) were thus investigated. The chromosome names provided here adhere to the nomenclature used in the reference genome ASM3628897v1. Variations in chromosome ploidy were found in 15 of the 81 isolates of *C. parapsilosis*. Specifically, trisomy of different chromosomes was detected (Fig. S4). Chromosome CP137568.1 trisomy was the most common, found in 11 isolates, all belonging to the main persistent clone resistant to azoles, trait that may be linked to the increased expression of the *ERG11* and *TAC1* genes, both located on this chromosome. This result was confirmed by the read coverage of the chromosome level hybrid assembly of isolate 35763_2_2, which is one of the 11 genomes containing this trisomy. Other chromosomal trisomies were reported in only one isolate: in 35763_2_48 for chromosome CP137570.1, in 36186_2_12 for chromosome CP137567.1 and in 36186_2_58 for chromosome CP137566.1. These chromosomes do not harbor any of the genes known to be determinants of antifungal resistance. Lastly, aneuploidy was detected in five different chromosomes (CP137570.1, CP137568.1, CP137566.1, CP137565.1, CP137569.1) in isolate 35763_2_42. Although aneuploidy and chromosomal trisomy have been associated with azole resistance in *Candida* species [11, 56], we cannot conclusively attribute a causal role to the presence of trisomy in mediating azole resistance in our dataset, as the patterns of chromosomal alteration was not coherent with the pattern of azole resistance.

## Discussion

In this study we provide insights into the genomic epidemiology and antifungal resistance of *Candida* spp. isolated from the hospital San Matteo, Pavia, Italy over a six-year period, until April 2020, during the first wave of COVID-19. Our dataset shows the onset of the COVID-19 pandemic caused a significant rise of fungal infections, highlighted by a notable increase of candidemia caused by *C. albicans* and *C. parapsilosis*, as previously reported [58]. *C. albicans* is still one of the most prevalent species reported in cases of candidemia worldwide, however a significant increase in infections caused by non-*albicans* species has been documented in many settings [4]. Among non-*albicans* species, *N. glabratus* has often been reported as the most abundant one in multiple geographic regions [59, 60], however, we did not observe a high prevalence (*n* = 3), a result consistent with the specific epidemiological scenario in Italy, where *C. albicans* and *C. parapsilosis* show higher prevalence [59, 61].

We performed a genomic characterization of two most prevalent *Candida* species circulating in Hospital San Matteo, *C. albicans* and *C. parapsilosis*. Phylogenomic analysis indicates that the *C. albicans* isolates were primarily transmitted through community-acquired infections rather than healthcare-associated outbreaks, in line with existing literature on *C. albicans* that reports mainly vertical transmissions [6]. We observed a different picture for *C. parapsilosis*, as we identified 81 isolates most belonging to two clonal groups (average SNPs distance of 36 and 11 for group A and B respectively). A SNPs difference threshold to define direct epidemiological links based on genomics is available for few bacterial and fungal species [62, 63], but to our knowledge it has not been determined yet for *C. parapsilosis* [37]. However, multiple studies identified low genetic variation among isolates of this species belonging to outbreaks (e.g. below 20 SNPs in [64] and below 57 in [63]). The two monophyletic clades described here clearly fall within the ranges previously reported. It must also be considered that our isolates were collected in a span of multiple years. The two monophyletic clusters can thus be classified as two single strains capable of persisting over time, and in such cases, we would expect them to gradually accumulate the reported low, but significant number of SNPs.

Interestingly, all isolates belonging to Group A were sensitive to all azoles, which could explain their limited success (fewer isolates) and eventual disappearance. The principal and successful clone (Group B), on the other hand, entered San Matteo hospital in 2018 and its highly effective spread was likely driven by resistance to fluconazole, a characteristic common in nosocomial *C. parapsilosis*. Global data support this hypothesis, as, the prevalence of fluconazole resistance in *C. parapsilosis* has been reported to be between 5 and 20%, increasing to up to 90% in outbreaks, leading to high persistence and spread of isolates within hospital environments [15, 65].

During the last three years of our surveillance, the Group B clone spread in the hospital, infecting multiple patients while developing resistance to voriconazole and itraconazole, finally causing a bona fide outbreak in 2020 during the first wave of COVID-19. The clinical relevance and nosocomial behaviour of Group B were further confirmed by statistical analyses, which highlighted a clear difference from Group A and sporadic isolates. A statistically longer delay between admission and pathogen isolation of isolates from Group B supports the nosocomial nature of this strain, clearly shown by the genomic data. Persistence in the hospital could be due to a combination of direct transmission between patients and persistence in the hospital environment. Furthermore, statisticall association with a longer hospitalisation period compared to Group A and sporadic cases suggests that the persistent Group B strain is more capable of causing severe infections, likely aided by its resistance to azoles. The similarities between Group A and sporadic cases further highlight the distinct characteristics of Group B isolates.

Given the high rates of azole resistance, we screened all *C. parapsilosis* isolates to identify potentially responsible mutations. In all 61 isolates belonging to the main persistent clone (Group B), we detected the Y132F amino acid substitution in *ERG11*, described as having a role in the clonal spread of fluconazole-resistance strains in hospital outbreaks [51, 65–67]. Along with this gene, Group B genomes show non-synonymous SNPs in *UPC2* and *MRR1*, reported to be involved in azole resistance. Specifically, the N455D substitution detected in Upc2 has been observed before [68], while the substitution identified in *MRR1* (S1907C) in this study represents a previously undescribed mutation. Notably, this mutation was observed in 60 of the 61 isolates belonging to the persistent clone, with the exception of the only isolates resistant to fluconazole but susceptible to the other three antifungal drugs (posaconazole, itraconazole and voriconazole). The absence of this mutation in this single isolate supports its potential implication in the development of resistance to posaconazole, itraconazole and voriconazole. However, further ad-hoc studies (for example monitoring patterns of gene expressions) and experimental validation are needed to determine whether this mutation is linked to the variations in the phenotypic resistance levels. In parallel, expanding the investigation to the hospital environment would represent an important future development, as it would allow to further evaluate the persistence in the hospital, and monitor the potential reservoirs and transmission routes of nosocomial strains.

Our study has the important limitation of having a partial sampling during the first three years of the examined period. Consequently, the number of cases during this earlier period may be underrepresented, which makes it difficult to assess candidemia incidence comprehensively in those years. Nevertheless, we included these data as they still provide valuable insights into the hospital’s epidemiological picture.

## Supplementary Information

Below is the link to the electronic supplementary material.

Supplementary file 1 (XLSX 27 KB)

Supplementary file 2 (XLSX 13 KB)

Supplementary file 3 (XLSX 11 KB)

Supplementary file 4 (PDF 3400 KB)

Supplementary file 5 (PDF 85 KB)

## Data Availability

Sequences and assemblies have been uploaded to the National Centre for Biotechnology Information (NCBI) together with the reads and the chromosome-level hybrid assembly of C. parapsilosis isolate with the primary accession code PRJNA1189032.
