# Supplementary material for: Genomic Characterization of Candida spp. Highlights a Persistent, Azole-Resistant C. parapsilosis Clone Circulating in a Tertiary Care Hospital During the First COVID-19 Wave
Source: Mycopathologia. 2026 Mar 16;191(2):44. doi: 10.1007/s11046-026-01070-9 (PMC12992396; doi:10.1007/s11046-026-01070-9)
Supplement: Supplementary file 4 — (PDF 3400 KB) [file 11046_2026_1070_MOESM4_ESM.pdf]

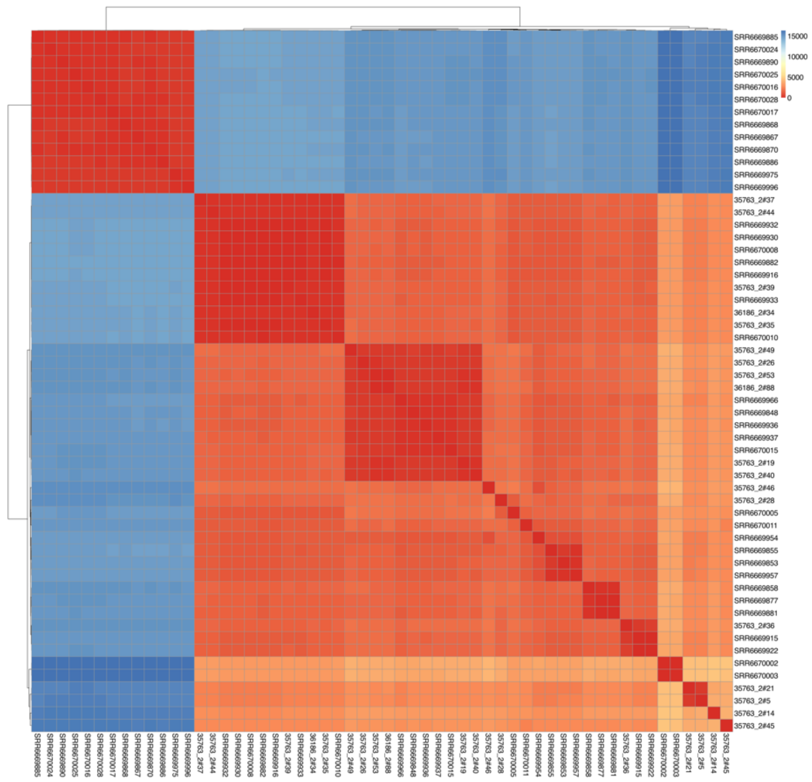

Figure S1. Heatmap of *C. albicans* SNPs showing the SNPs distance between all pairs of genomes included in the analysis, comprising isolates from San Matteo Hospital in Pavia and genomes retrieved from public datasets.

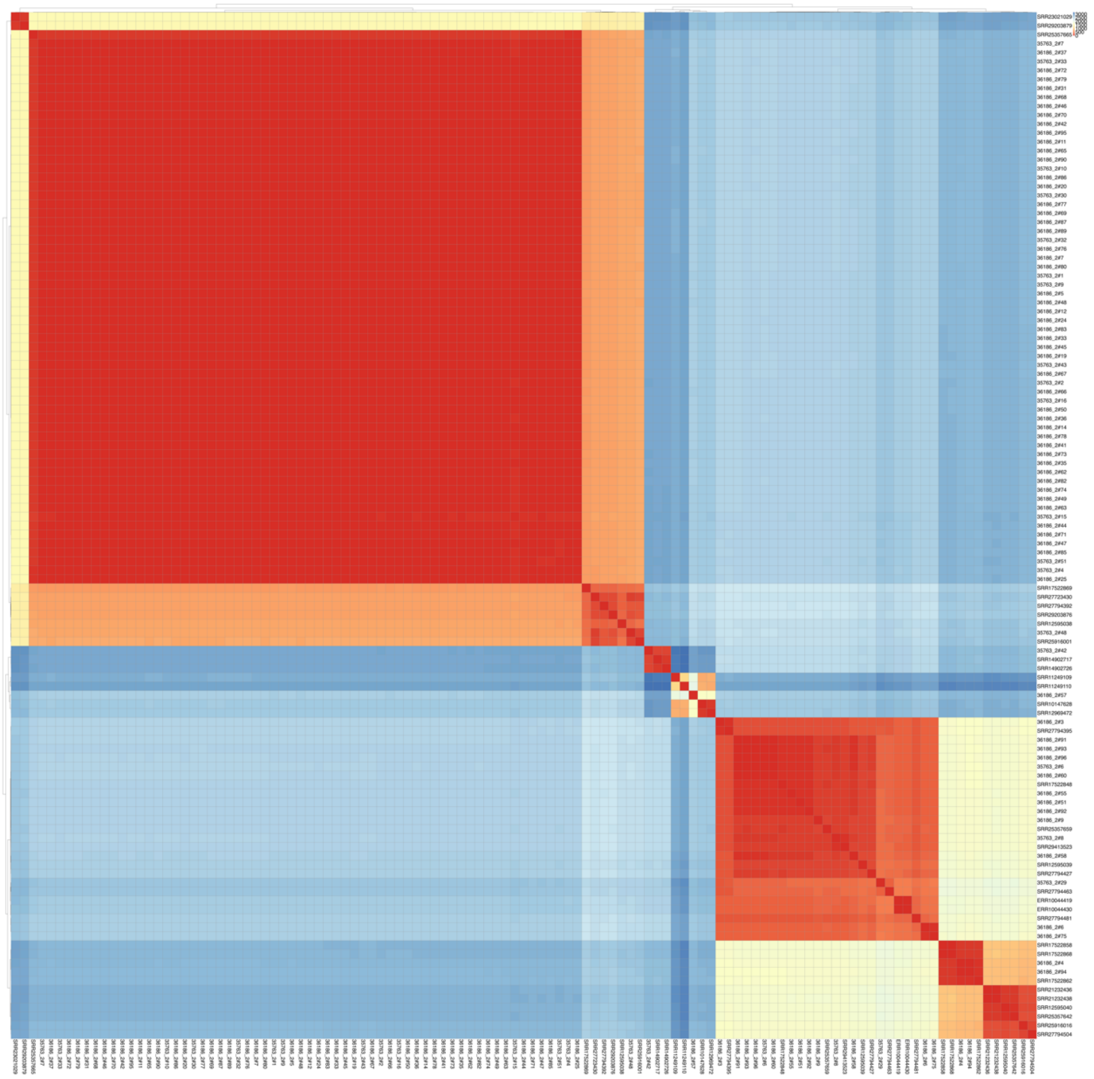

Figure S2. Heatmap of *C. parapsilosis* SNPs showing the SNPs distance between all pairs of genomes included in the analysis, comprising isolates from San Matteo Hospital in Pavia and genomes retrieved from public datasets.

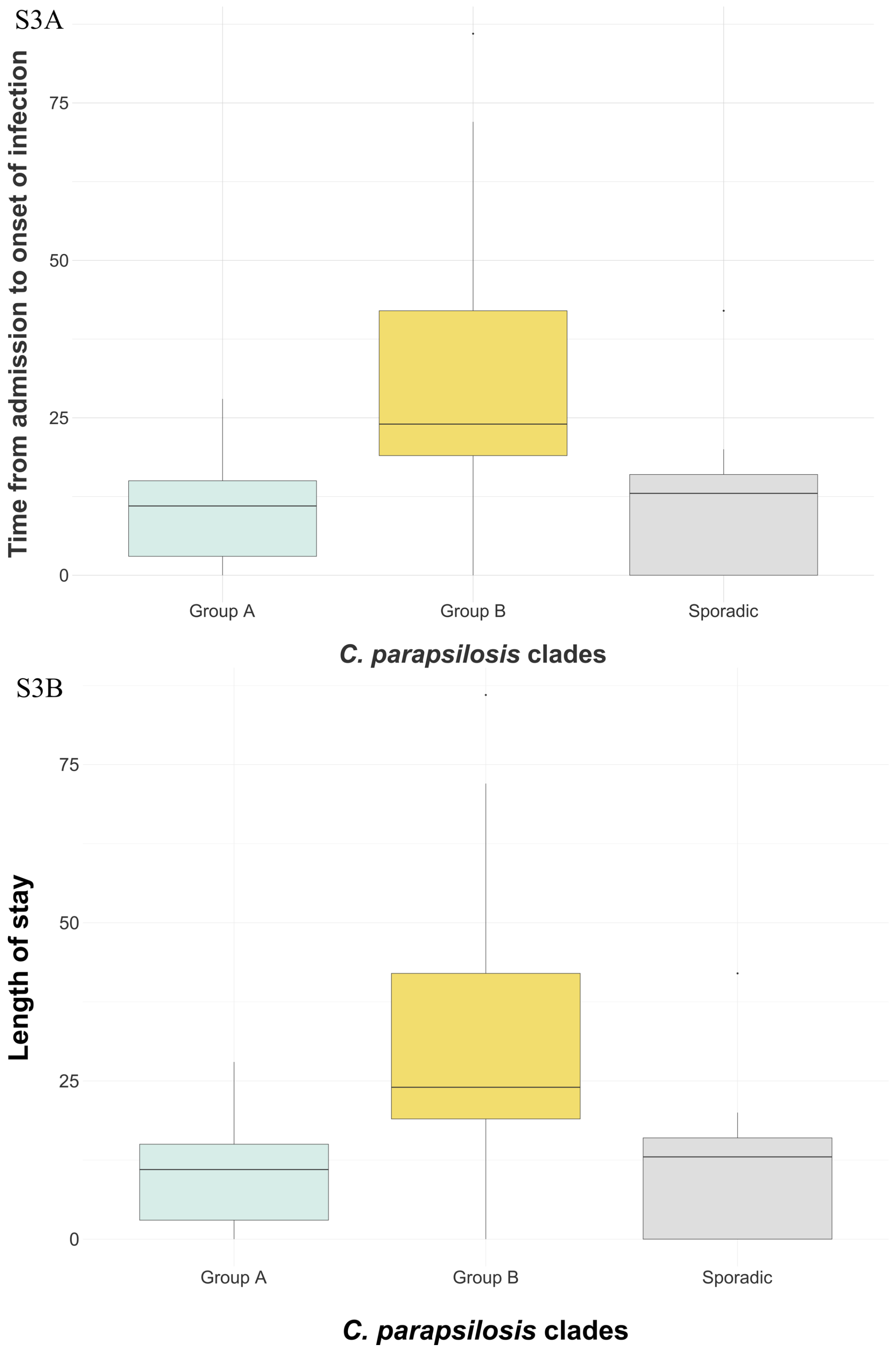

Figure S3. Distribution of (A) time (in days) from admission to onset of infection and (B) total length of stay (in days) across *C. parapsilosis* groups. The boxplots illustrate the median (horizontal line), the interquartile range (box), and the minimum/maximum values (whiskers). Outliers are represented as individual points.

Figure S4. Delly plot of the 81 *C. parapsilosis* isolates representing chromosome aneuploidy.

35763_2_1 whole genome

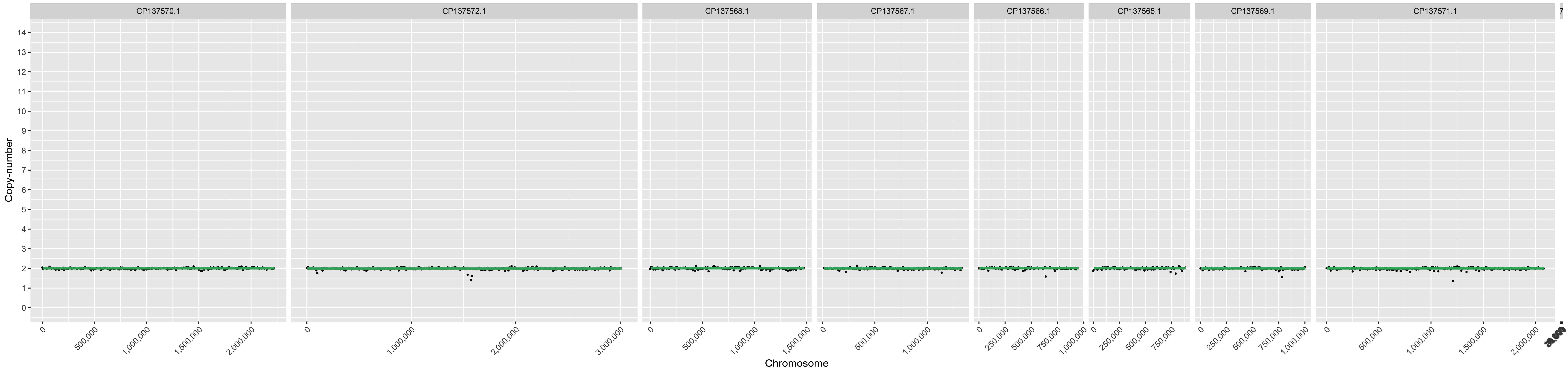

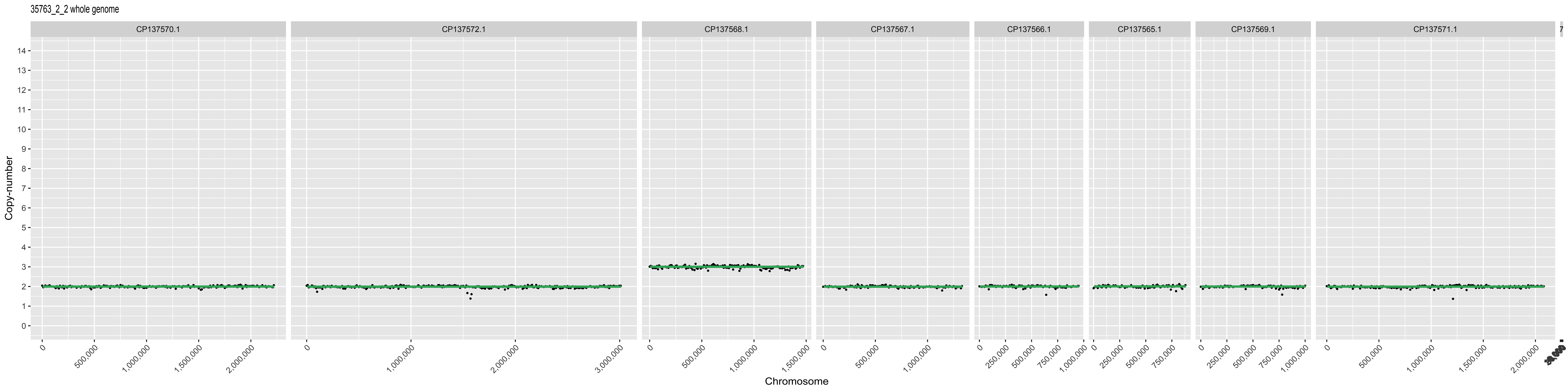

35763_2_2 whole genome
CP137570.1
CP137572.1
CP137568.1
CP137567.1
CP137566.1
CP137565.1
CP137569.1
CP137571.1
Copy-number
Chromosome

35763_2_4 whole genome

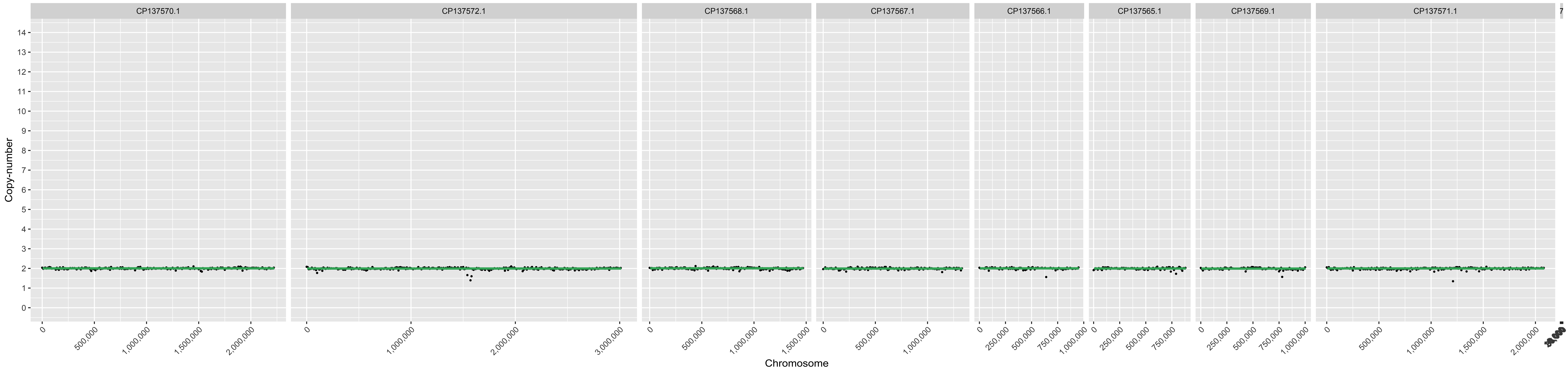

35763_2_6 whole genome

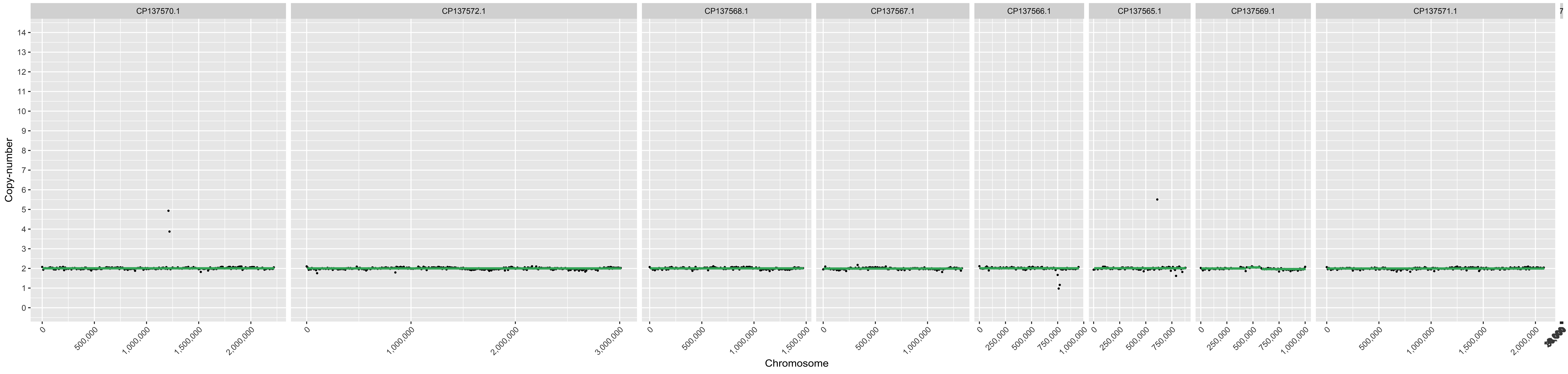

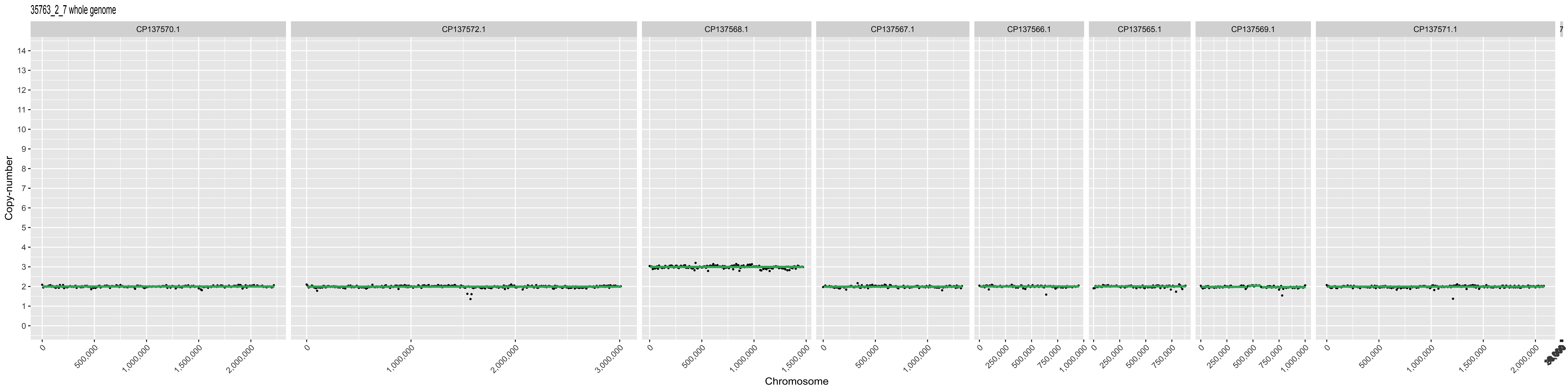
35763_2_7 whole genome
CP137570.1
CP137572.1
CP137568.1
CP137567.1
CP137566.1
CP137565.1
CP137569.1
CP137571.1
Copy-number
Chromosome

35763_2_8 whole genome

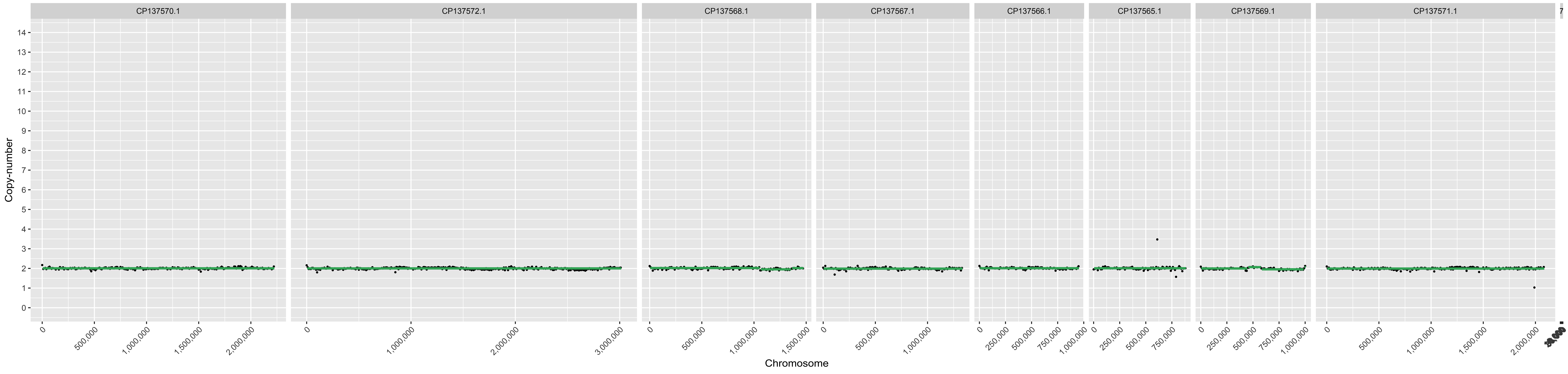

35763_2_9 whole genome

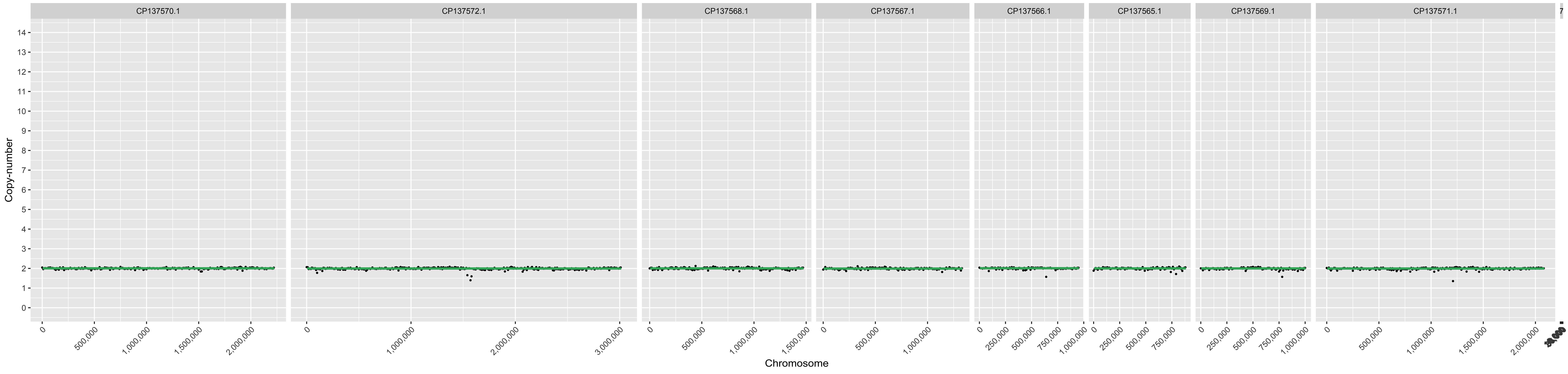

35763_2_10 whole genome

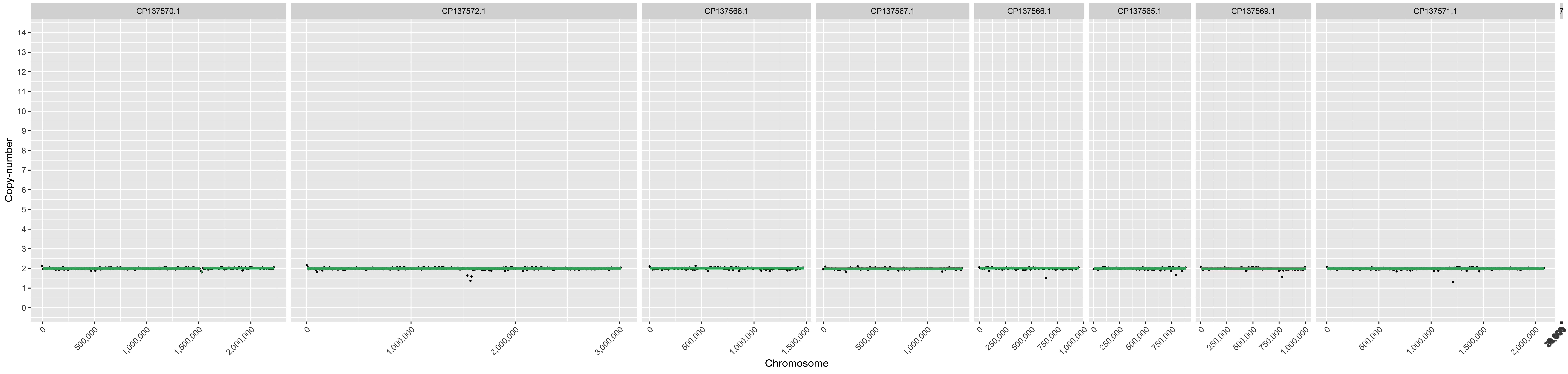

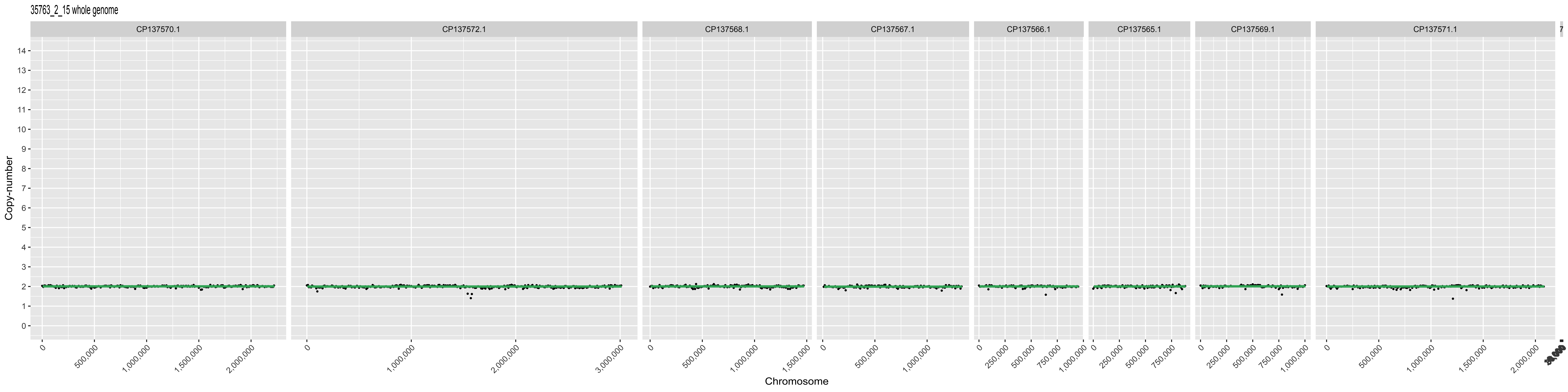
35763_2_15 whole genome
CP137570.1
CP137572.1
CP137568.1
CP137567.1
CP137566.1
CP137565.1
CP137569.1
CP137571.1
Copy-number
Chromosome
0
500,000
1,000,000
1,500,000
2,000,000
3,000,000
250,000
750,000

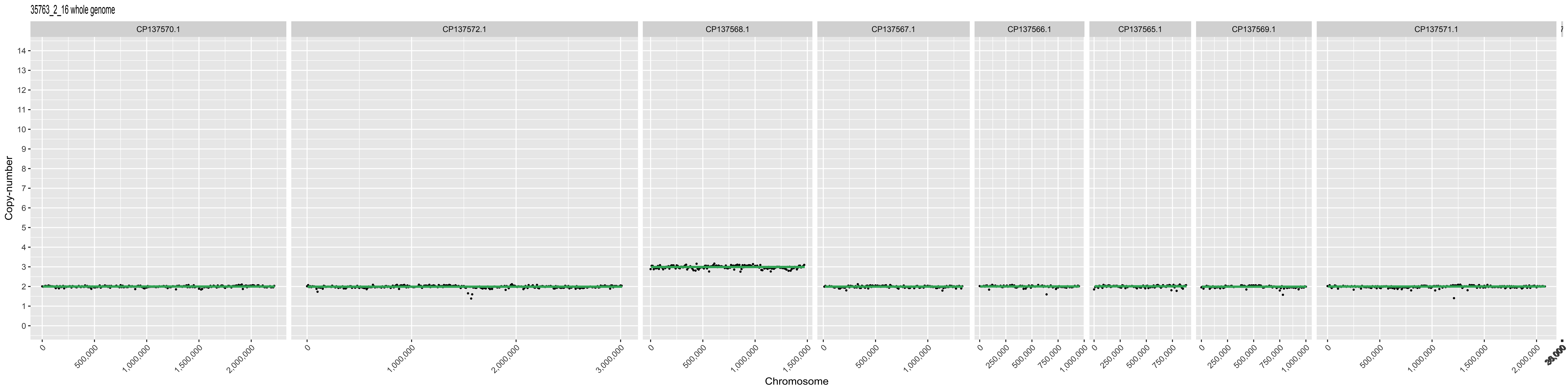

35763_2_16 whole genome
CP137570.1
CP137572.1
CP137568.1
CP137567.1
CP137566.1
CP137565.1
CP137569.1
CP137571.1
Copy-number
Chromosome

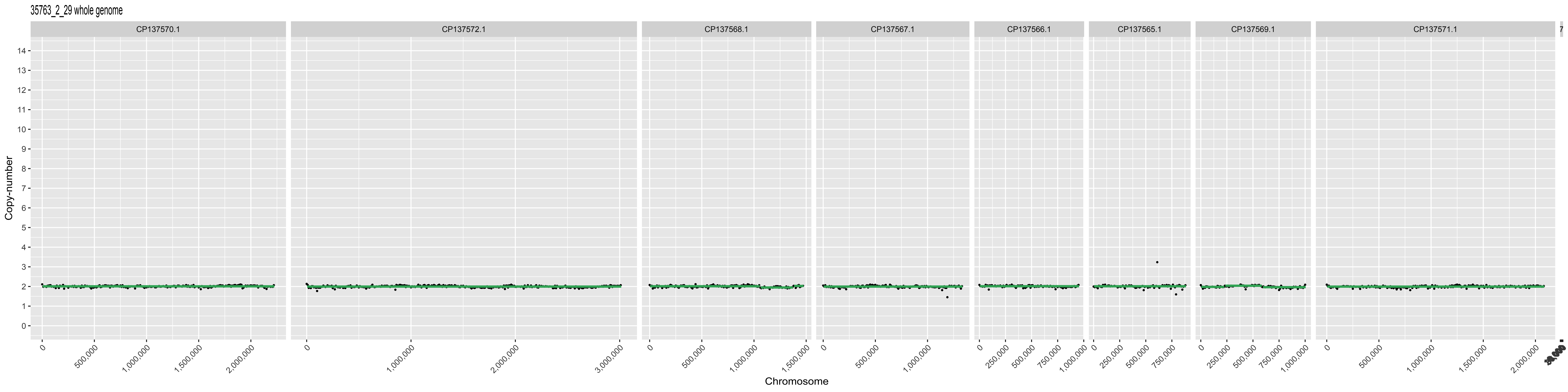
35763_2_29 whole genome
CP137570.1
CP137572.1
CP137568.1
CP137567.1
CP137566.1
CP137565.1
CP137569.1
CP137571.1
Copy-number
Chromosome
0
500,000
1,000,000
1,500,000
2,000,000
3,000,000
250,000
750,000

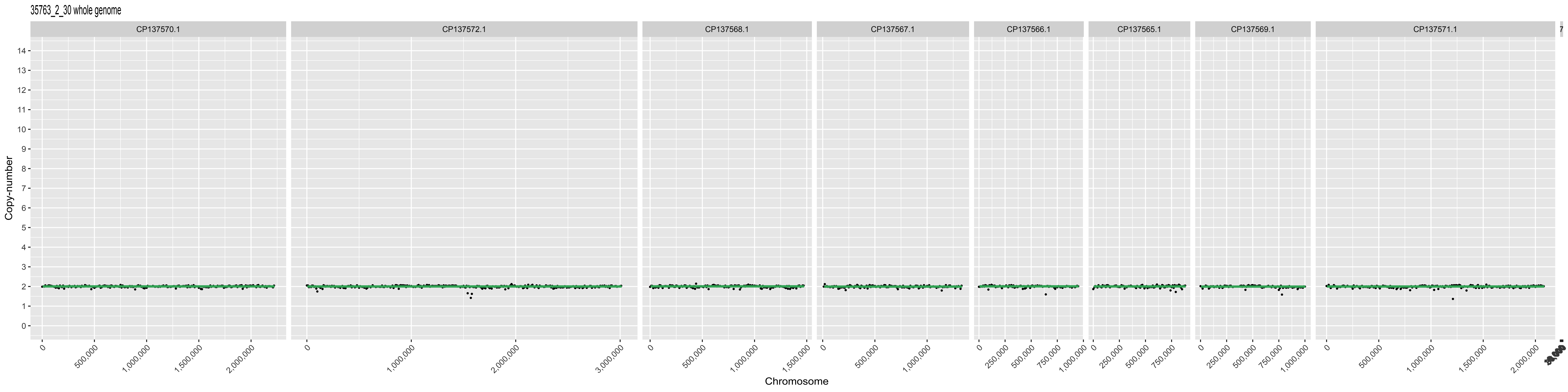
35763_2_30 whole genome
CP137570.1
CP137572.1
CP137568.1
CP137567.1
CP137566.1
CP137565.1
CP137569.1
CP137571.1
Copy-number
Chromosome
0
1
2
3
4
5
6
7
8
9
10
11
12
13
14
500,000
1,000,000
1,500,000
2,000,000
3,000,000
250,000
750,000

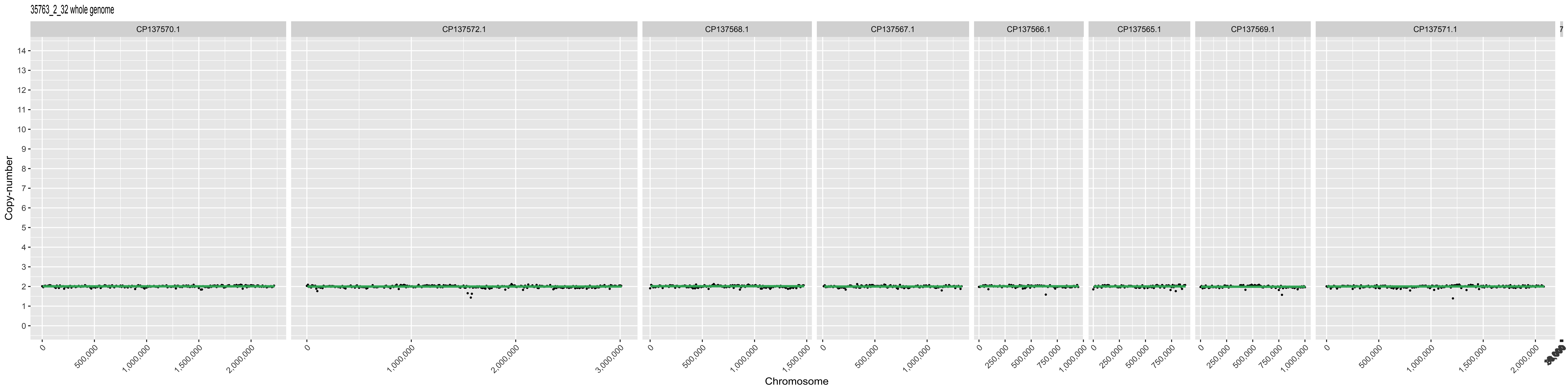
35763_2_32 whole genome
CP137570.1
CP137572.1
CP137568.1
CP137567.1
CP137566.1
CP137565.1
CP137569.1
CP137571.1
Copy-number
Chromosome

35763_2_33 whole genome

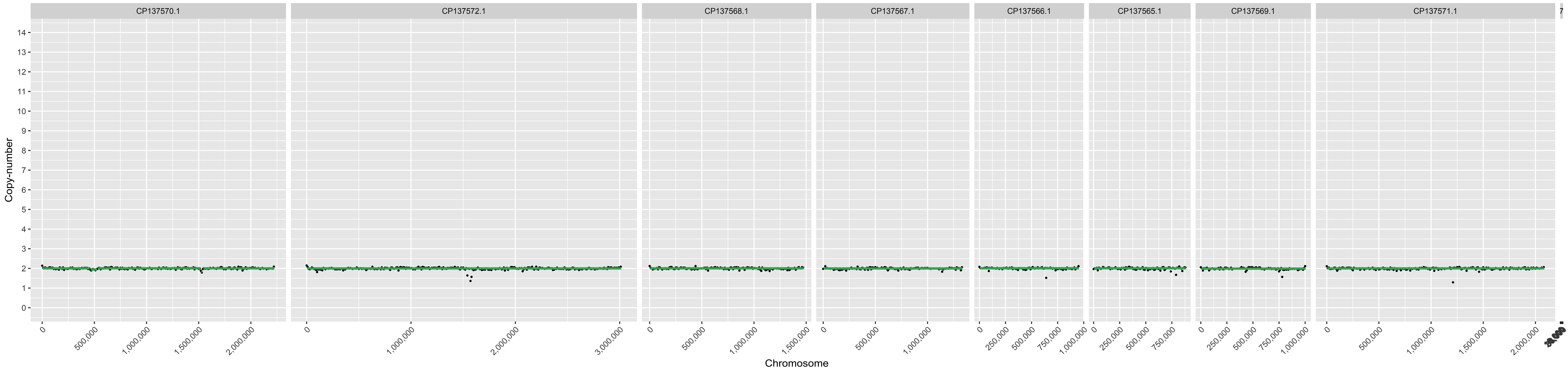

35763_2_42 whole genome

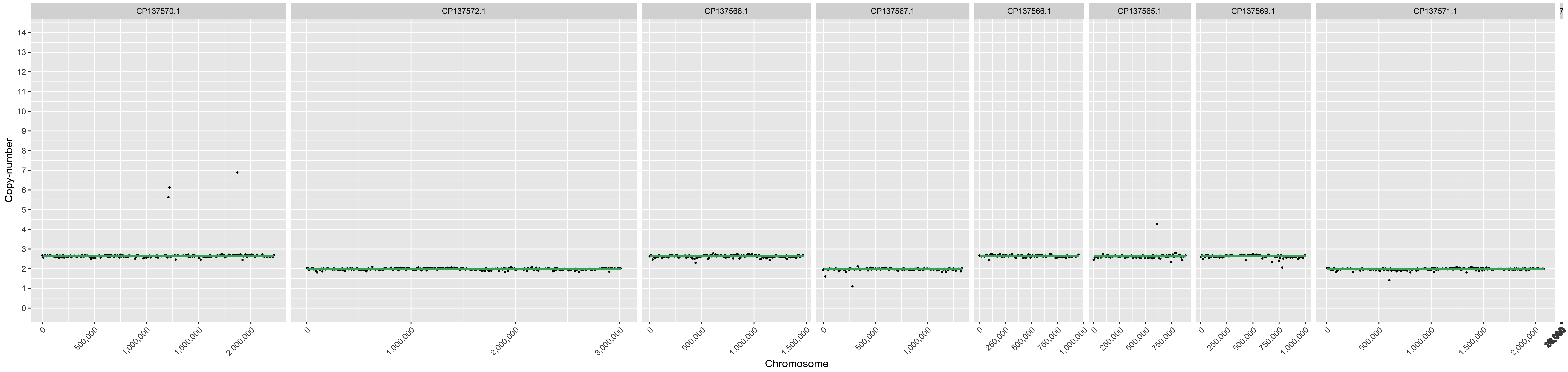

35763_2_43 whole genome

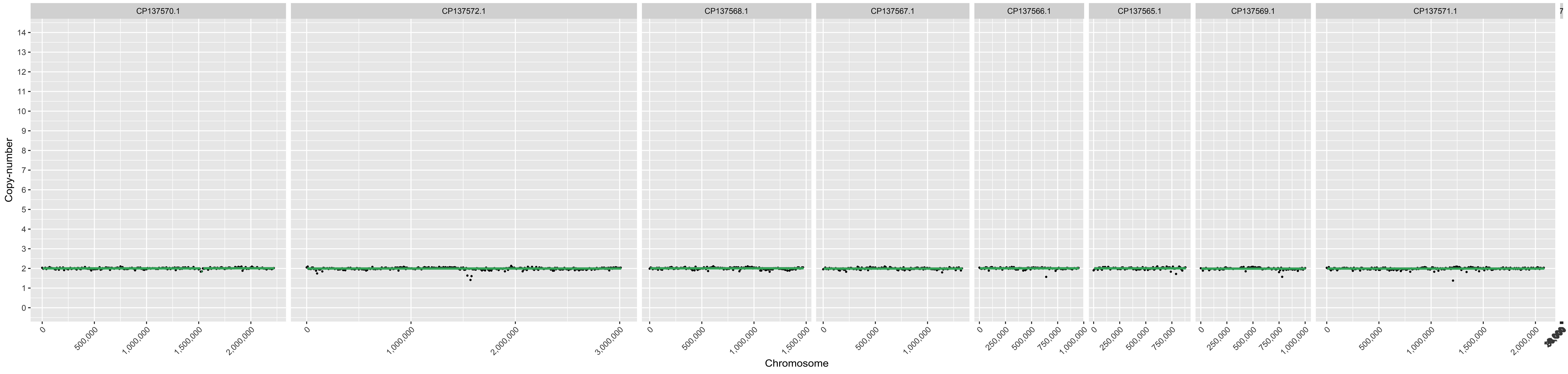

35763_2_48 whole genome

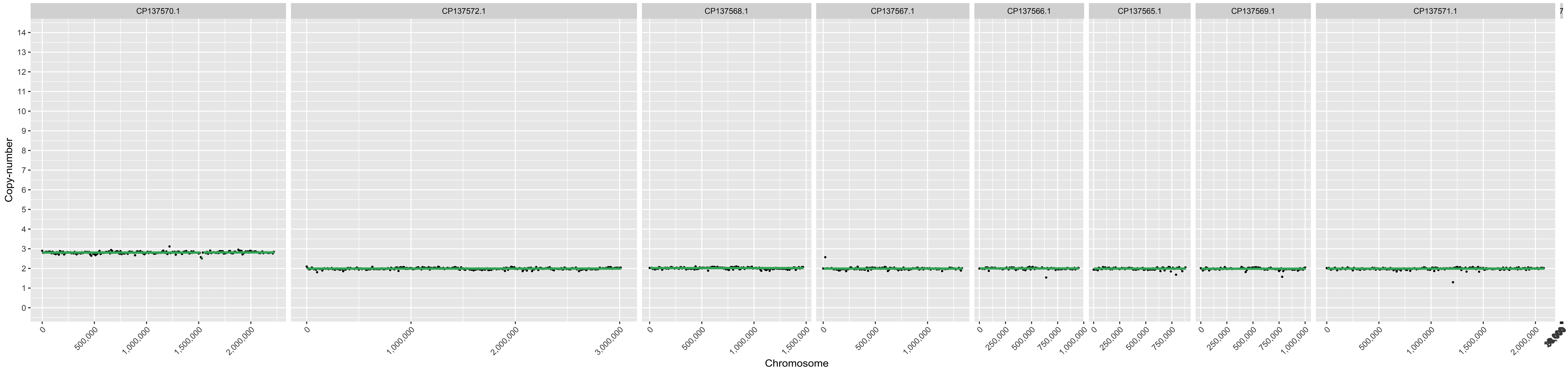

35763_2_51 whole genome

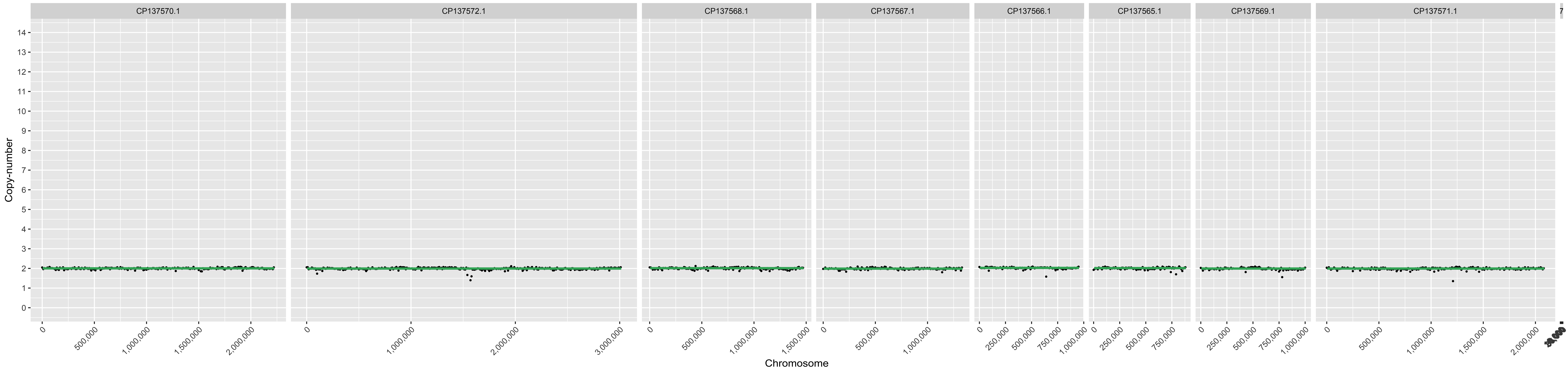

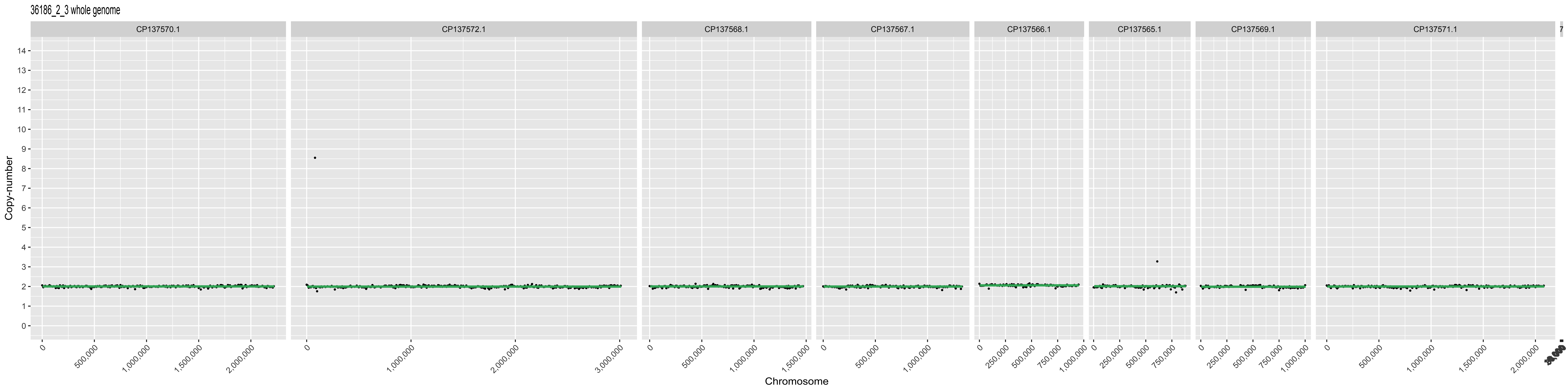

36186_2_3 whole genome
CP137570.1
CP137572.1
CP137568.1
CP137567.1
CP137566.1
CP137565.1
CP137569.1
CP137571.1
Copy-number
Chromosome

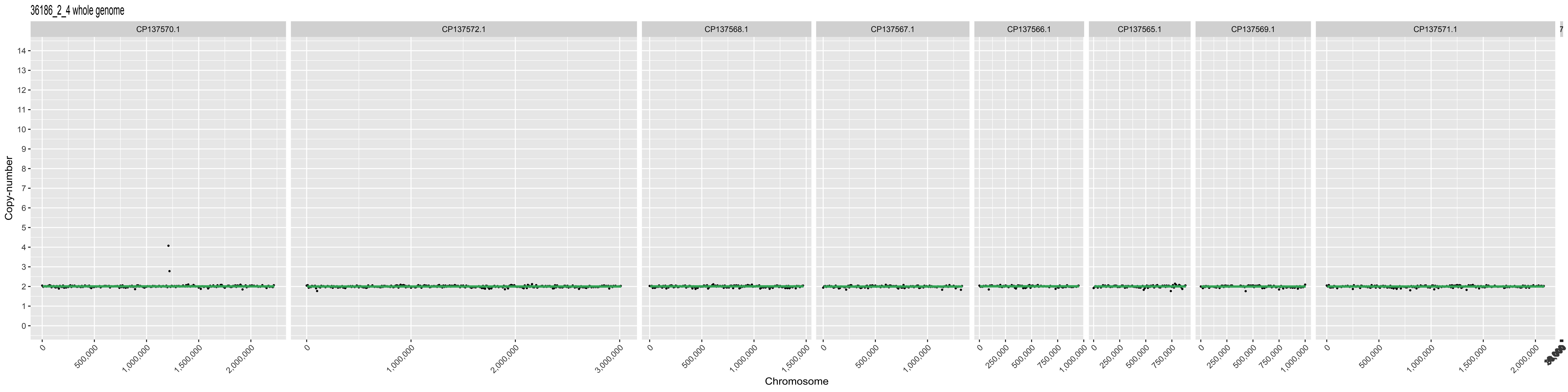

36186_2_4 whole genome
CP137570.1
CP137572.1
CP137568.1
CP137567.1
CP137566.1
CP137565.1
CP137569.1
CP137571.1
Copy-number
Chromosome

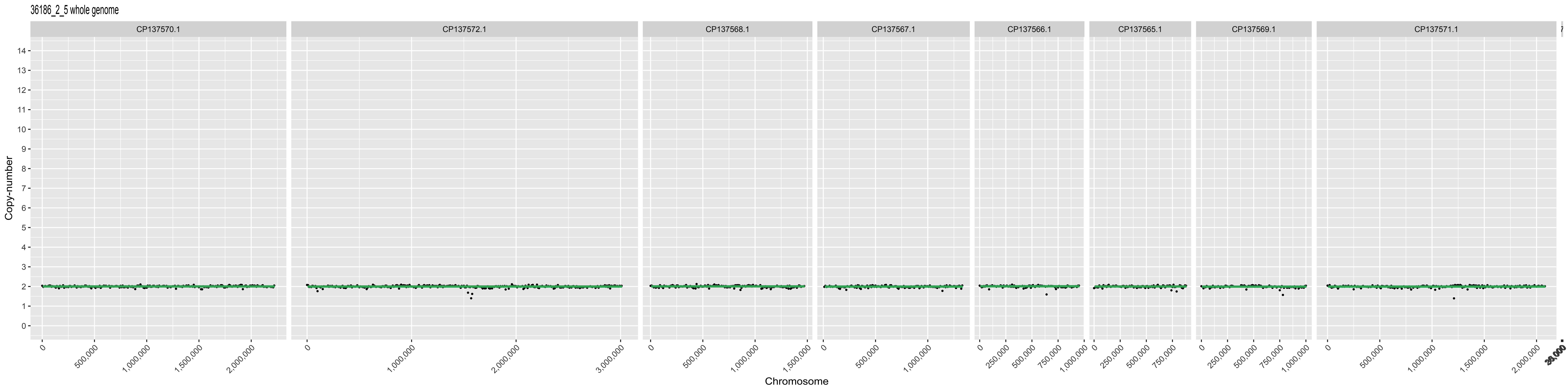

36186_2_5 whole genome
CP137570.1
CP137572.1
CP137568.1
CP137567.1
CP137566.1
CP137565.1
CP137569.1
CP137571.1
Copy-number
Chromosome

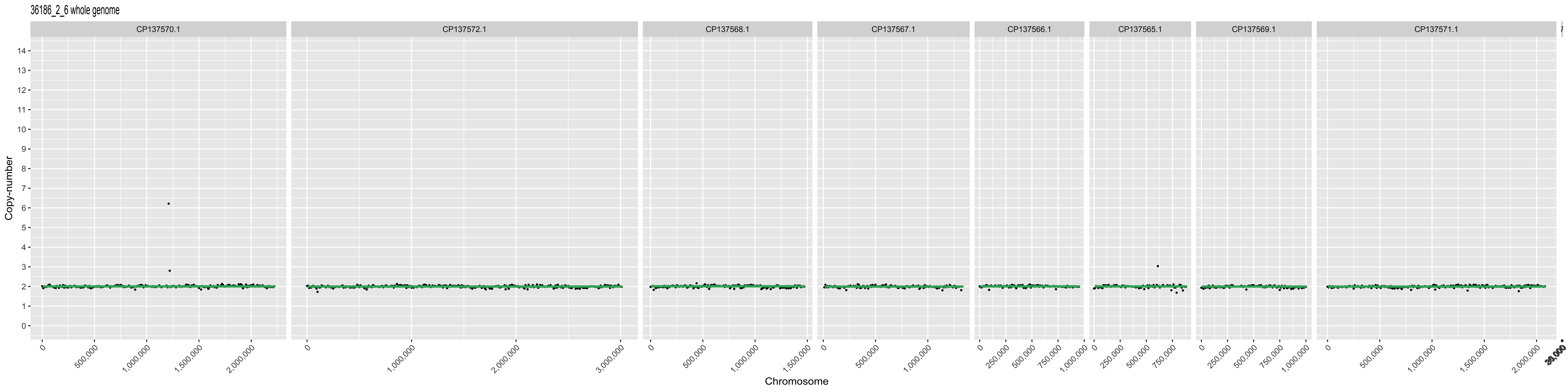

36186_2_6 whole genome
CP137570.1
CP137572.1
CP137568.1
CP137567.1
CP137566.1
CP137565.1
CP137569.1
CP137571.1
Copy-number
Chromosome

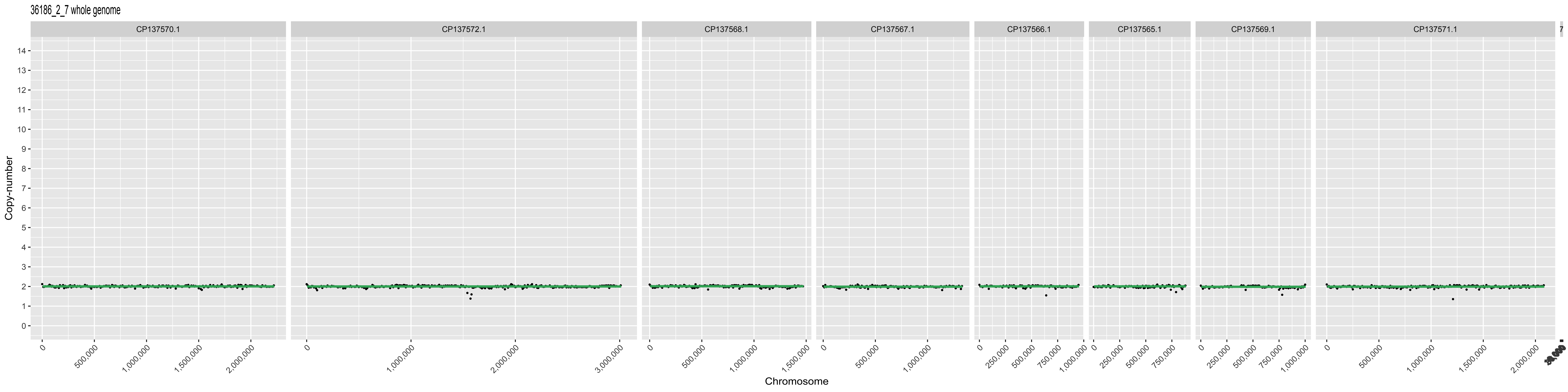

36186_2_7 whole genome
CP137570.1
CP137572.1
CP137568.1
CP137567.1
CP137566.1
CP137565.1
CP137569.1
CP137571.1
Copy-number
Chromosome

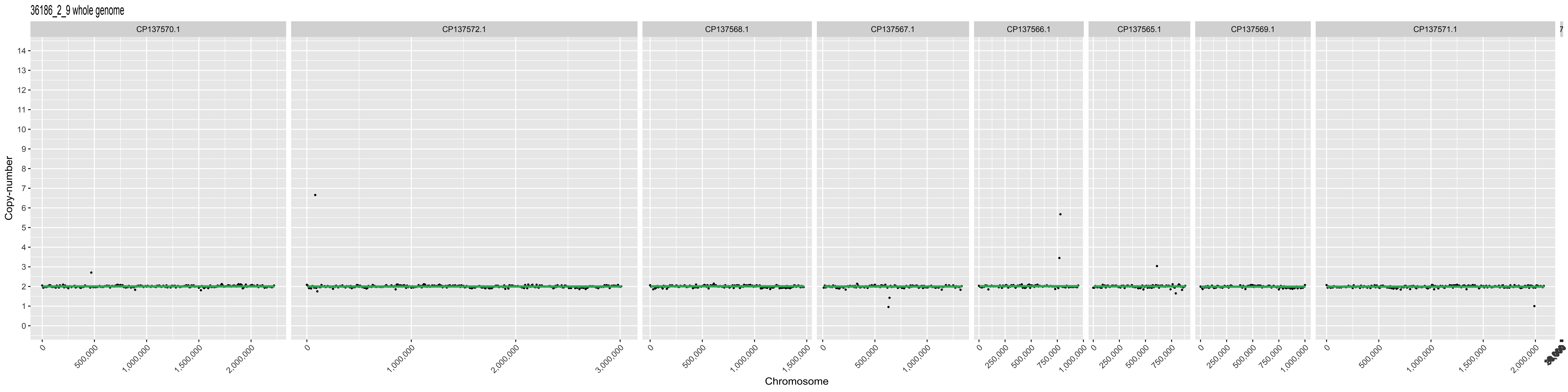
36186_2_9 whole genome
CP137570.1
CP137572.1
CP137568.1
CP137567.1
CP137566.1
CP137565.1
CP137569.1
CP137571.1
Copy-number
Chromosome

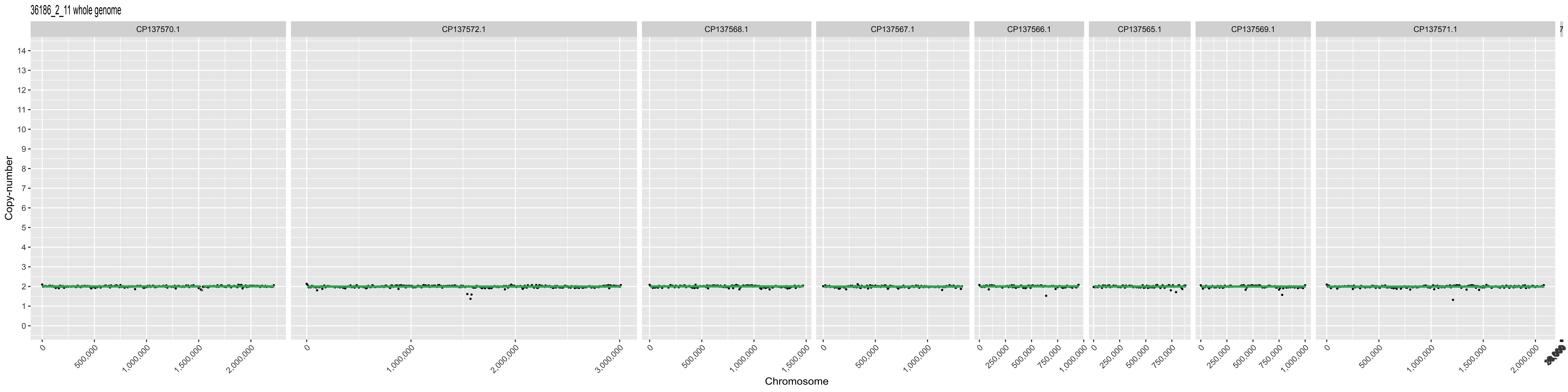

36186_2_11 whole genome
CP137570.1
CP137572.1
CP137568.1
CP137567.1
CP137566.1
CP137565.1
CP137569.1
CP137571.1
Copy-number
Chromosome

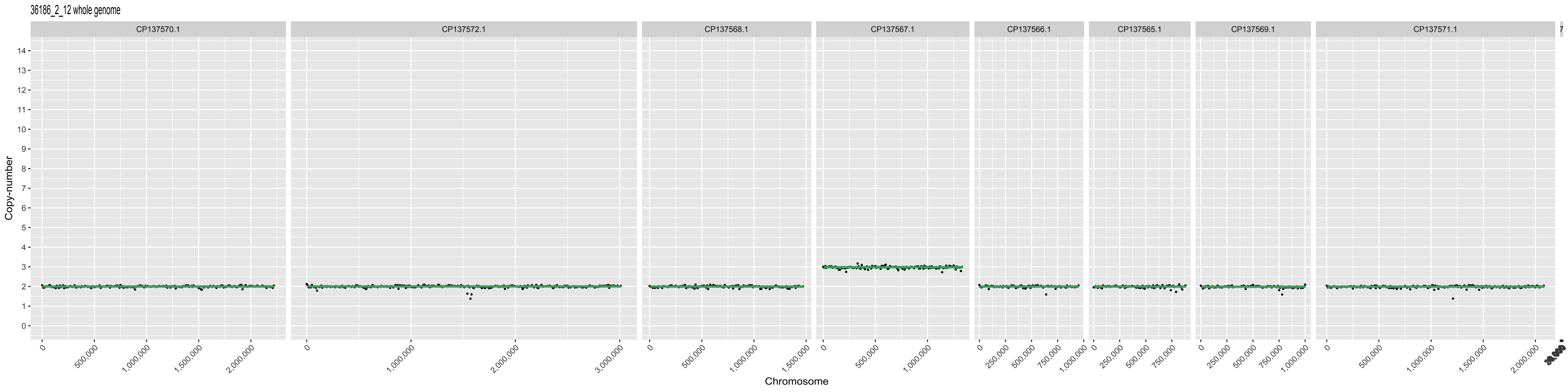

36186_2_12 whole genome
CP137570.1
CP137572.1
CP137568.1
CP137567.1
CP137566.1
CP137565.1
CP137569.1
CP137571.1
Copy-number
0
1
2
3
4
5
6
7
8
9
10
11
12
13
14
Chromosome
0
500,000
1,000,000
1,500,000
2,000,000
3,000,000
250,000
750,000

36186_2_14 whole genome

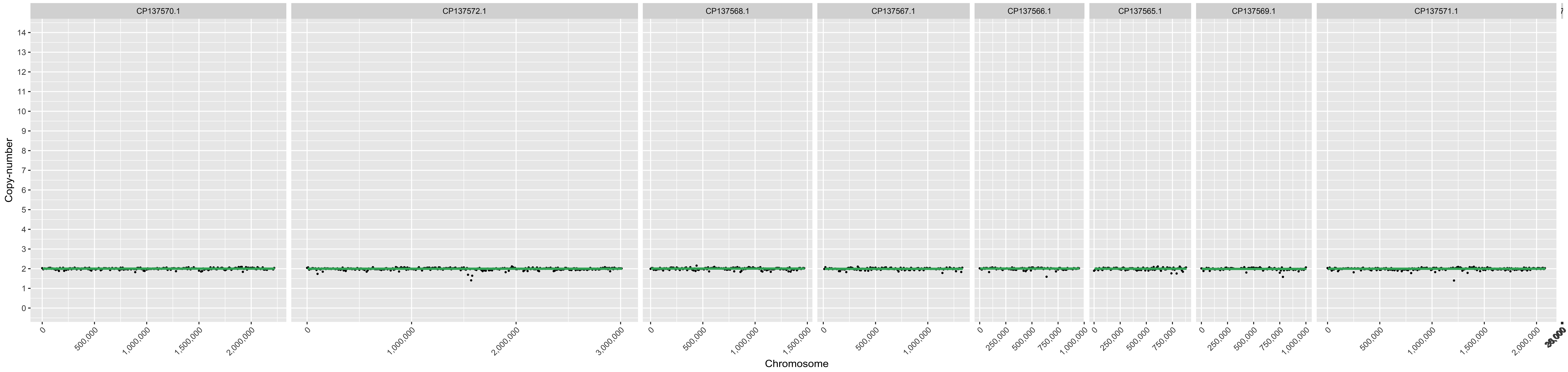

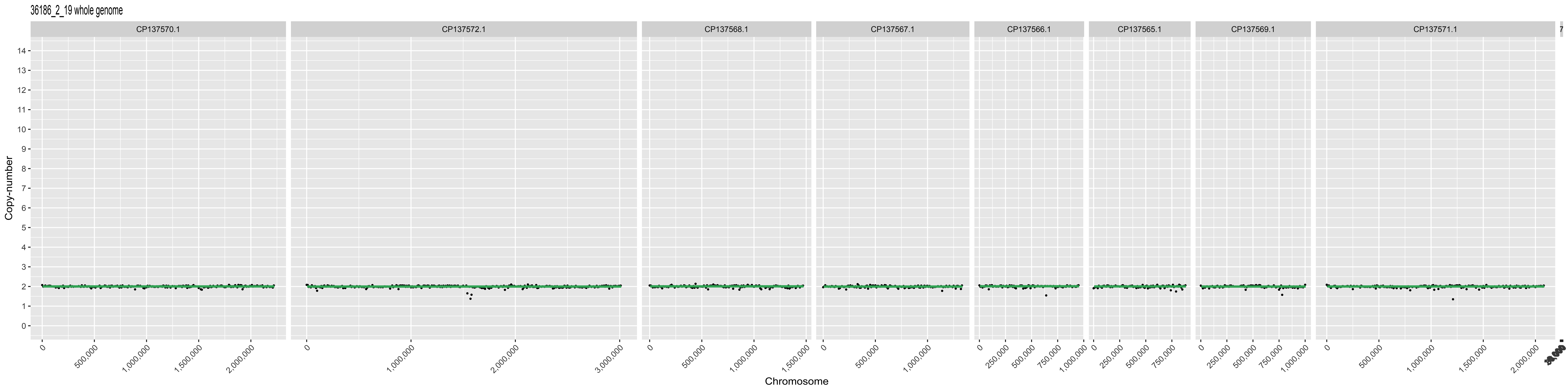
36186_2_19 whole genome
CP137570.1
CP137572.1
CP137568.1
CP137567.1
CP137566.1
CP137565.1
CP137569.1
CP137571.1
Copy-number
Chromosome

36186_2_20 whole genome

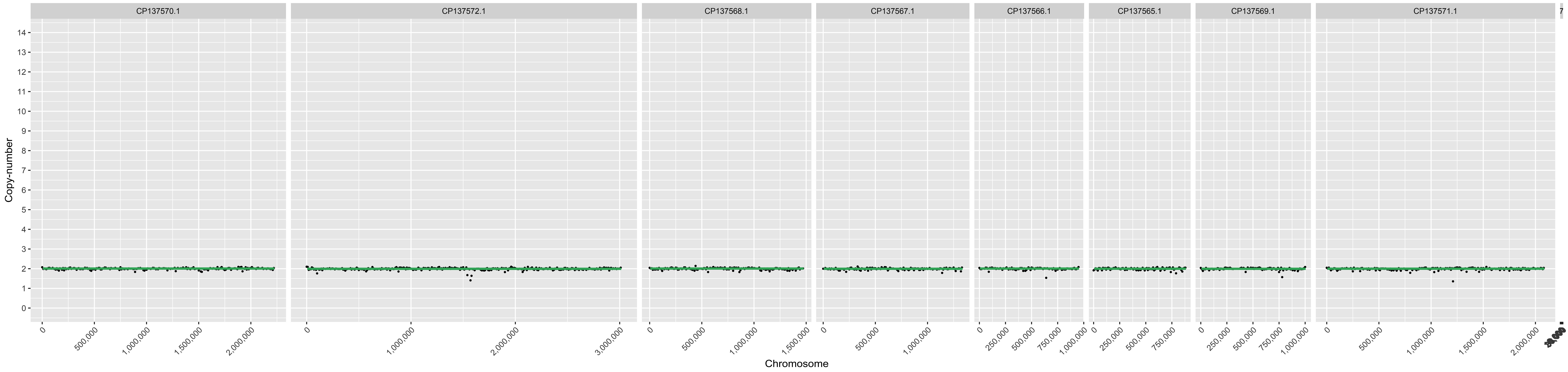

36186_2_24 whole genome

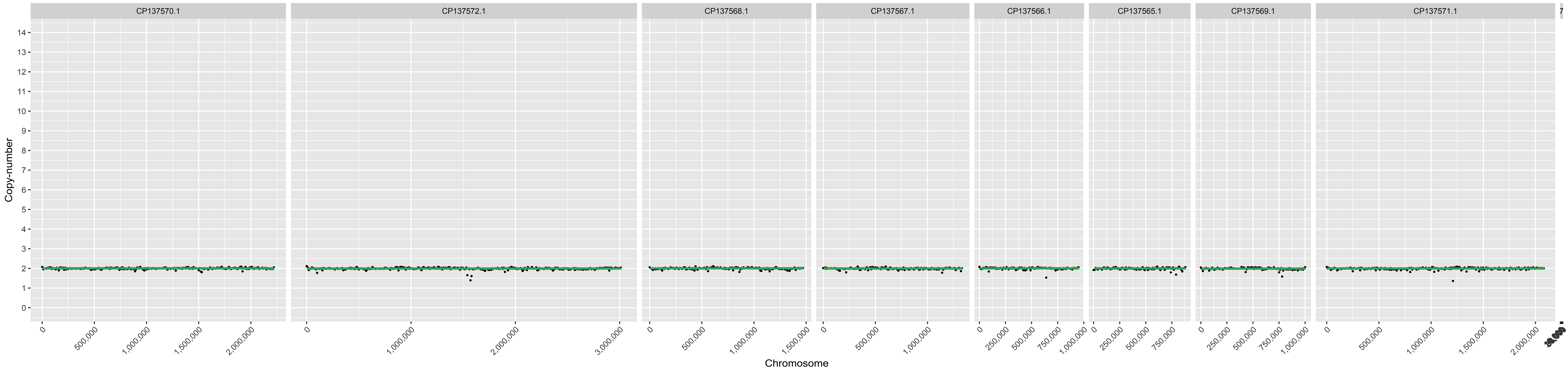

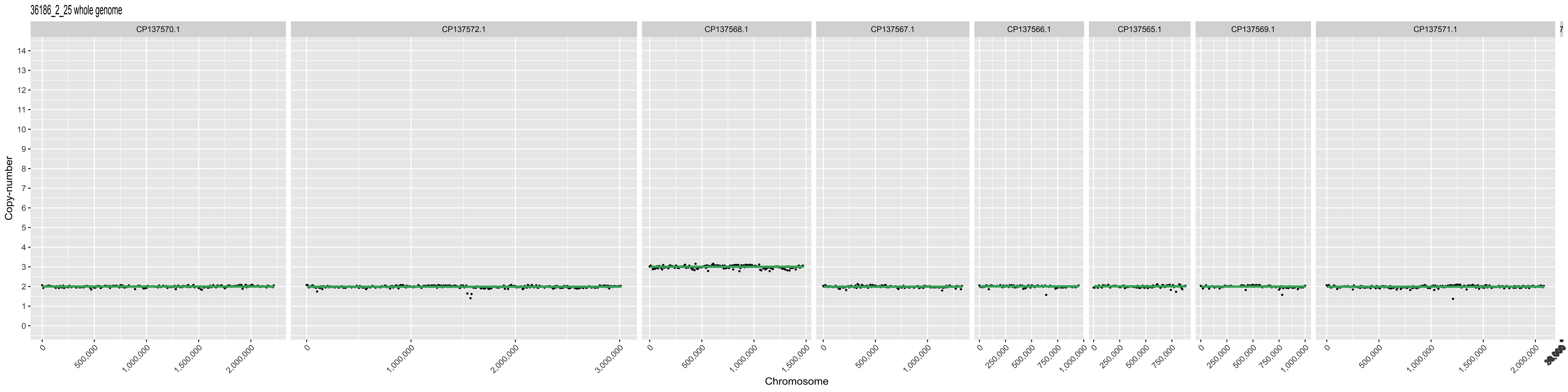

36186_2_25 whole genome
CP137570.1
CP137572.1
CP137568.1
CP137567.1
CP137566.1
CP137565.1
CP137569.1
CP137571.1
Copy-number
Chromosome

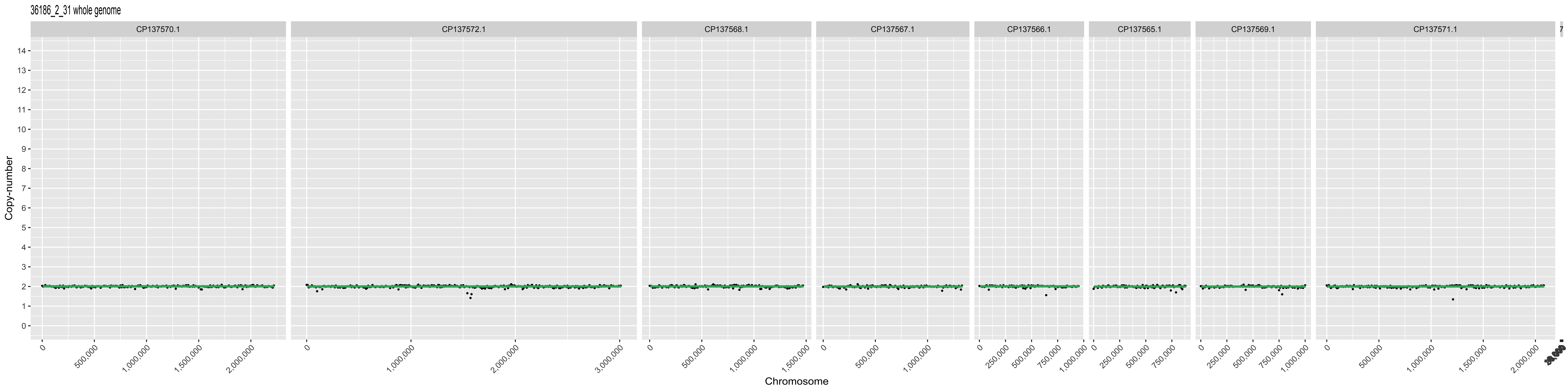
36186_2_31 whole genome
CP137570.1
CP137572.1
CP137568.1
CP137567.1
CP137566.1
CP137565.1
CP137569.1
CP137571.1
Copy-number
0
1
2
3
4
5
6
7
8
9
10
11
12
13
14
0
500,000
1,000,000
1,500,000
2,000,000
3,000,000
250,000
750,000
Chromosome

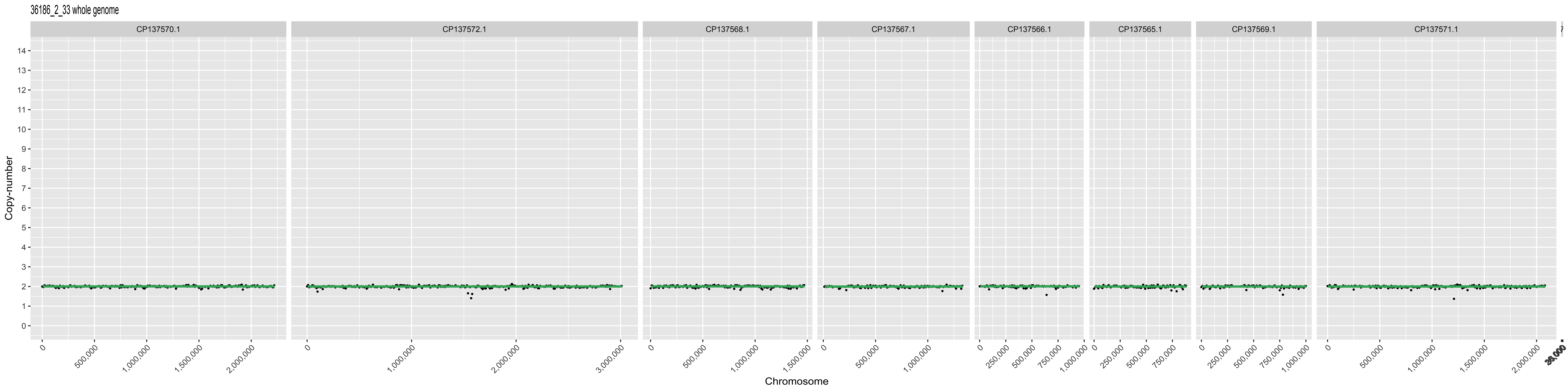
36186_2_33 whole genome
CP137570.1
CP137572.1
CP137568.1
CP137567.1
CP137566.1
CP137565.1
CP137569.1
CP137571.1
Copy-number
Chromosome

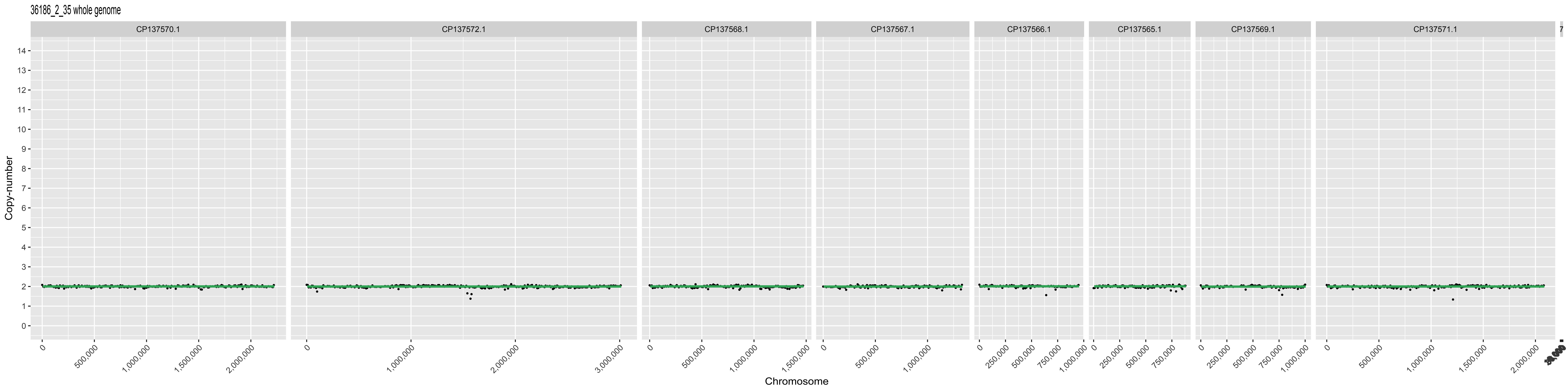
36186_2_35 whole genome
CP137570.1
CP137572.1
CP137568.1
CP137567.1
CP137566.1
CP137565.1
CP137569.1
CP137571.1
Copy-number
Chromosome
0
500,000
1,000,000
1,500,000
2,000,000
3,000,000
250,000
750,000

36186_2_36 whole genome

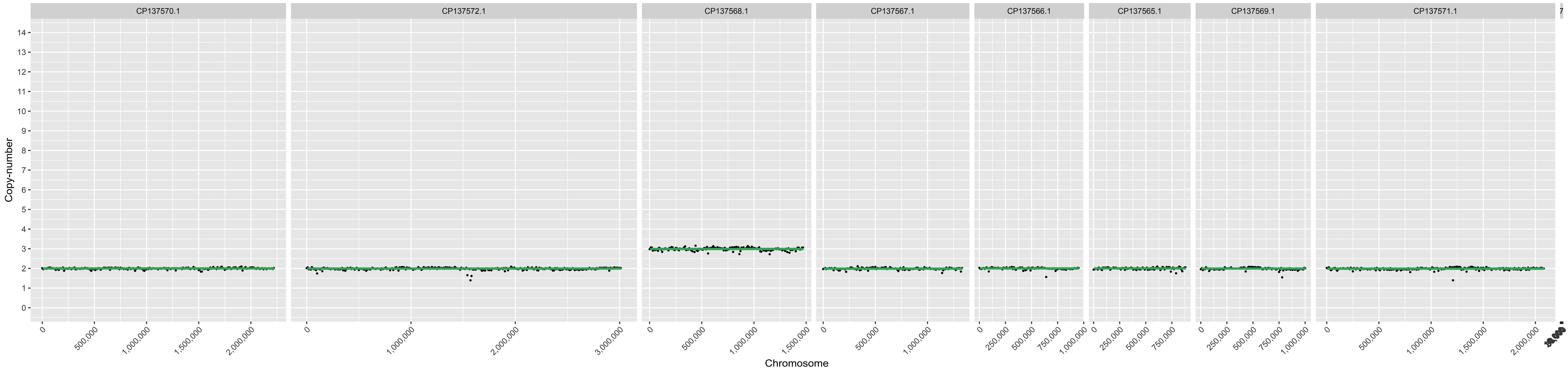

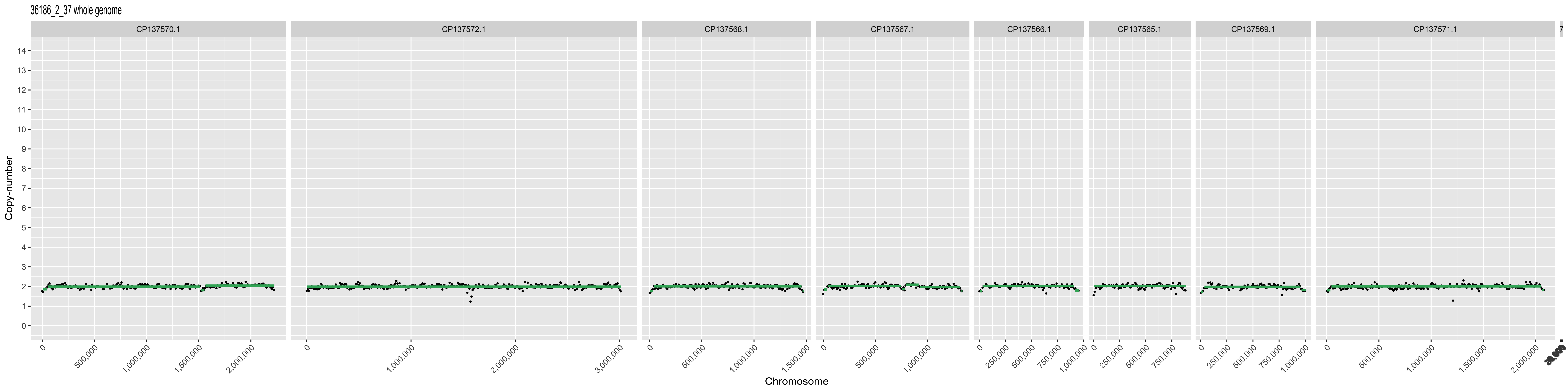
36186_2_37 whole genome
CP137570.1
CP137572.1
CP137568.1
CP137567.1
CP137566.1
CP137565.1
CP137569.1
CP137571.1
Copy-number
Chromosome

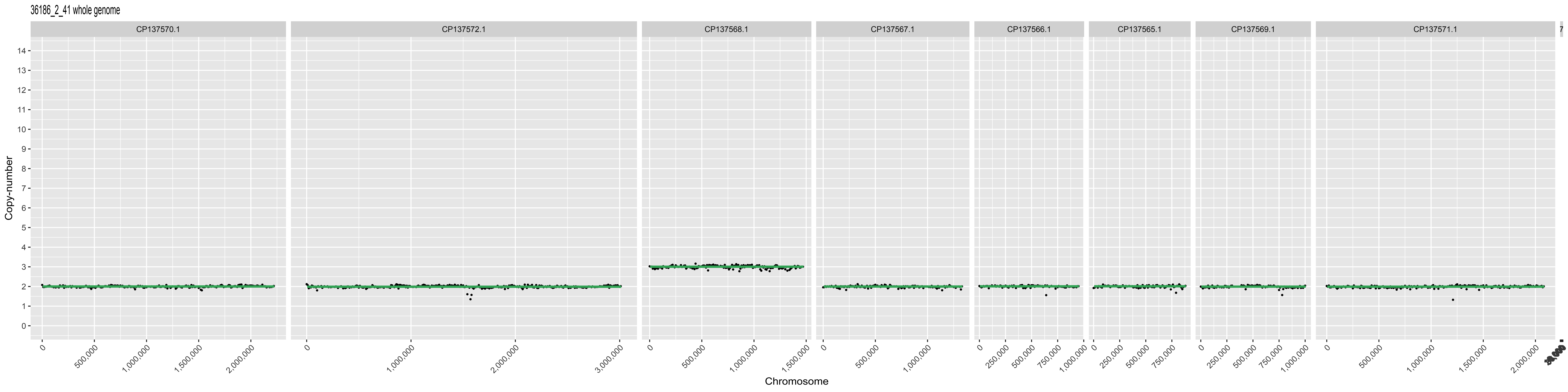

36186_2_41 whole genome
CP137570.1
CP137572.1
CP137568.1
CP137567.1
CP137566.1
CP137565.1
CP137569.1
CP137571.1
Copy-number
Chromosome

36186_2_42 whole genome

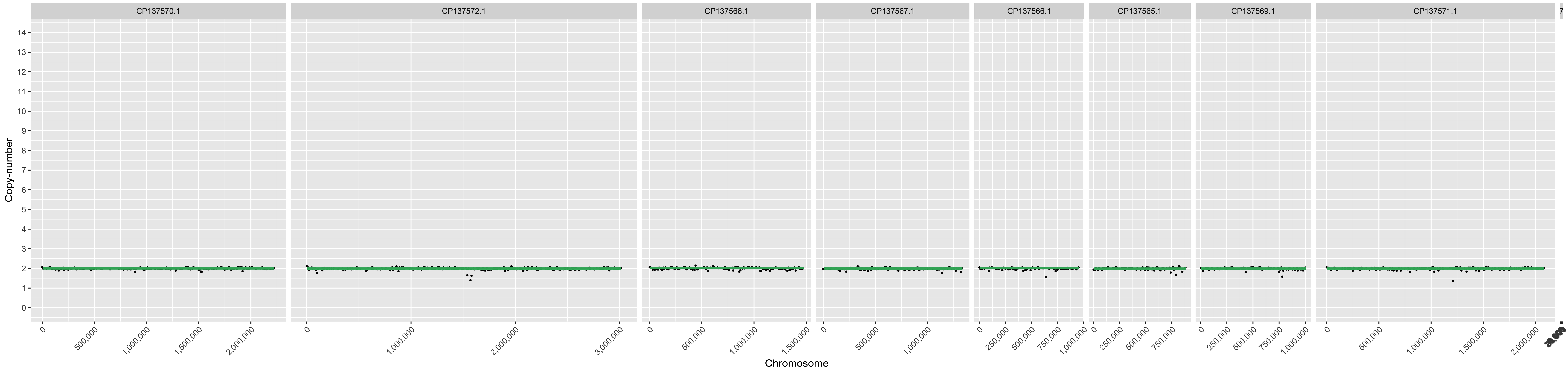

36186_2_44 whole genome

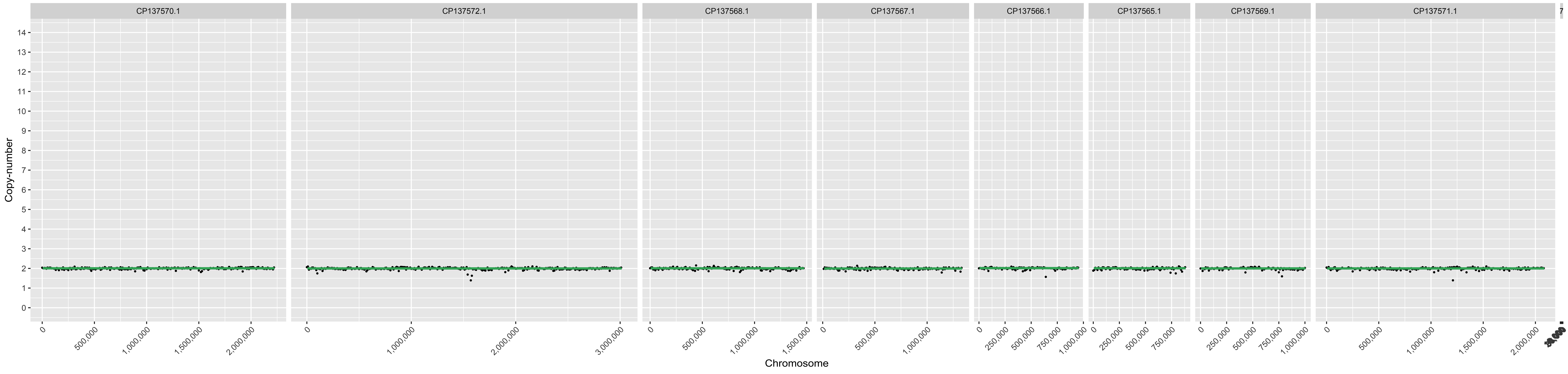

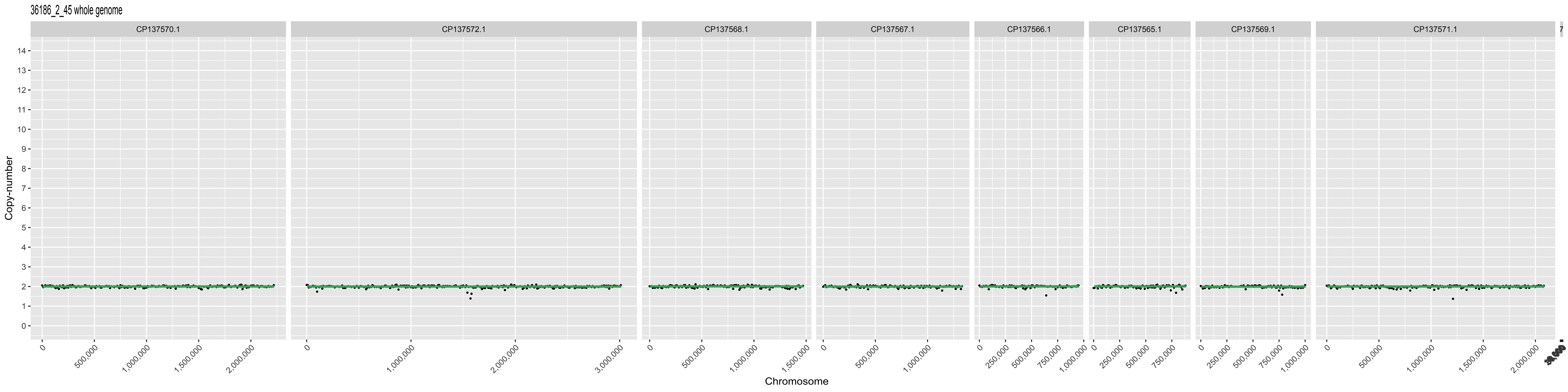
36186_2_45 whole genome
CP137570.1
CP137572.1
CP137568.1
CP137567.1
CP137566.1
CP137565.1
CP137569.1
CP137571.1
Copy-number
Chromosome
0
1
2
3
4
5
6
7
8
9
10
11
12
13
14
500,000
1,000,000
1,500,000
2,000,000
3,000,000
250,000
750,000

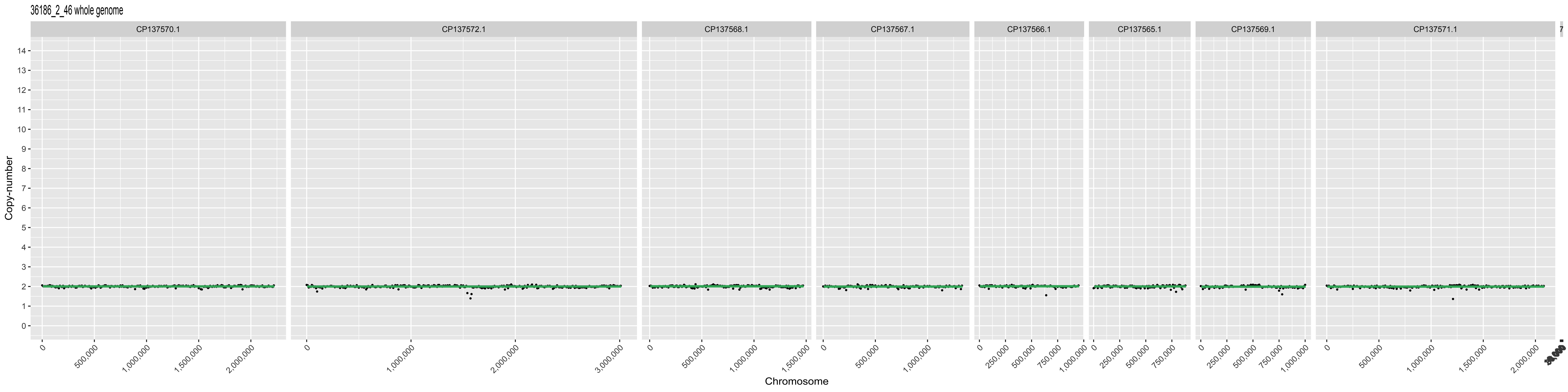

36186_2_46 whole genome
CP137570.1
CP137572.1
CP137568.1
CP137567.1
CP137566.1
CP137565.1
CP137569.1
CP137571.1
Copy-number
Chromosome

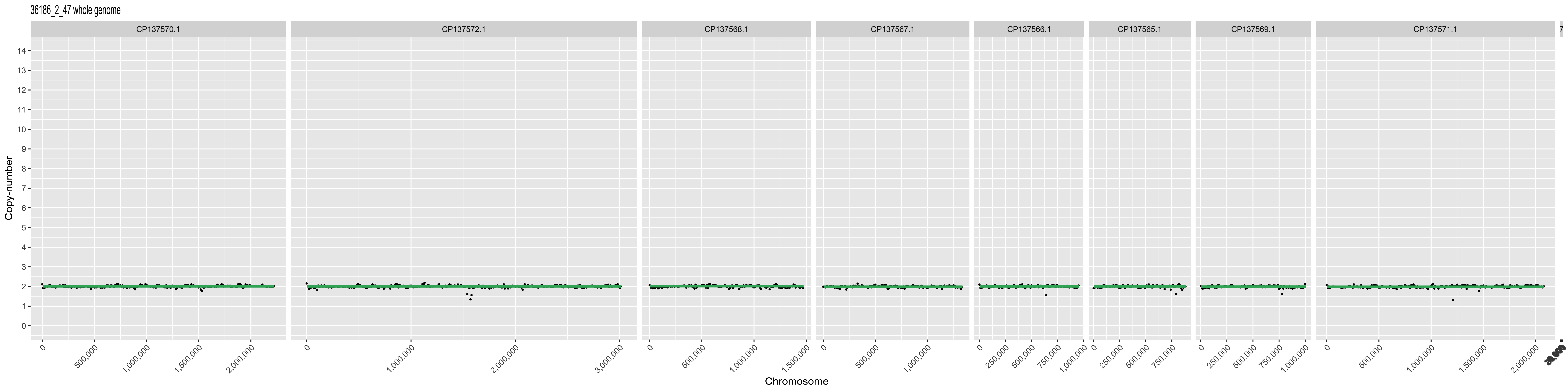
36186_2_47 whole genome
CP137570.1
CP137572.1
CP137568.1
CP137567.1
CP137566.1
CP137565.1
CP137569.1
CP137571.1
Copy-number
Chromosome
0
500,000
1,000,000
1,500,000
2,000,000
3,000,000
250,000
750,000

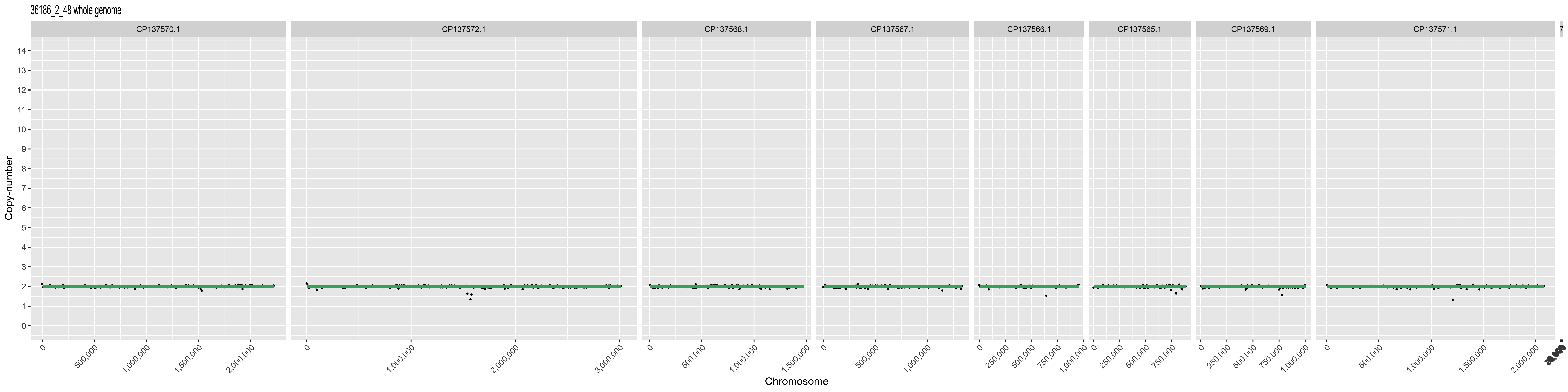

36186_2_48 whole genome
CP137570.1
CP137572.1
CP137568.1
CP137567.1
CP137566.1
CP137565.1
CP137569.1
CP137571.1
Copy-number
Chromosome

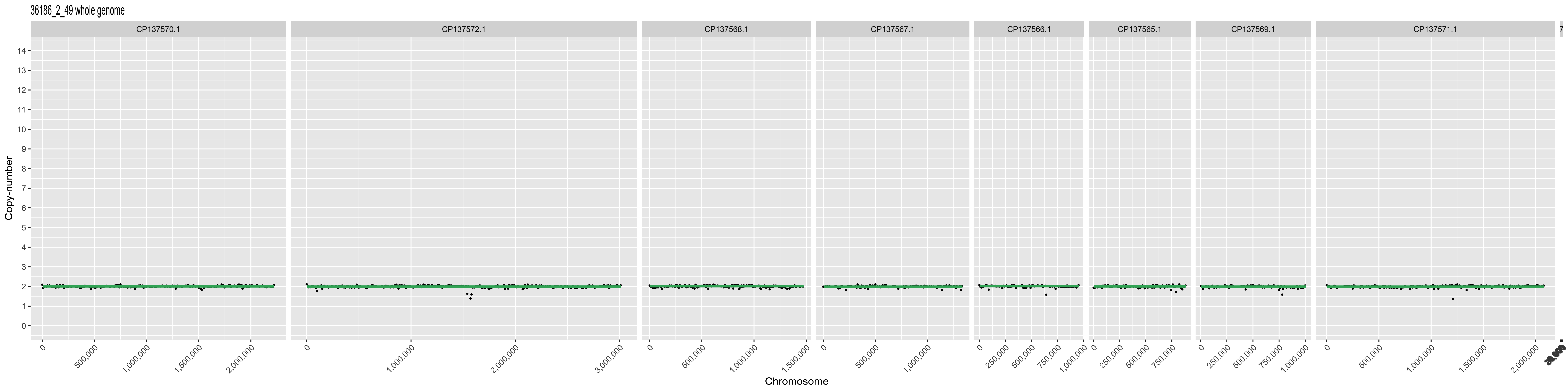

36186_2_49 whole genome
CP137570.1
CP137572.1
CP137568.1
CP137567.1
CP137566.1
CP137565.1
CP137569.1
CP137571.1
Copy-number
Chromosome
0
1
2
3
4
5
6
7
8
9
10
11
12
13
14
500,000
1,000,000
1,500,000
2,000,000
3,000,000
250,000
750,000

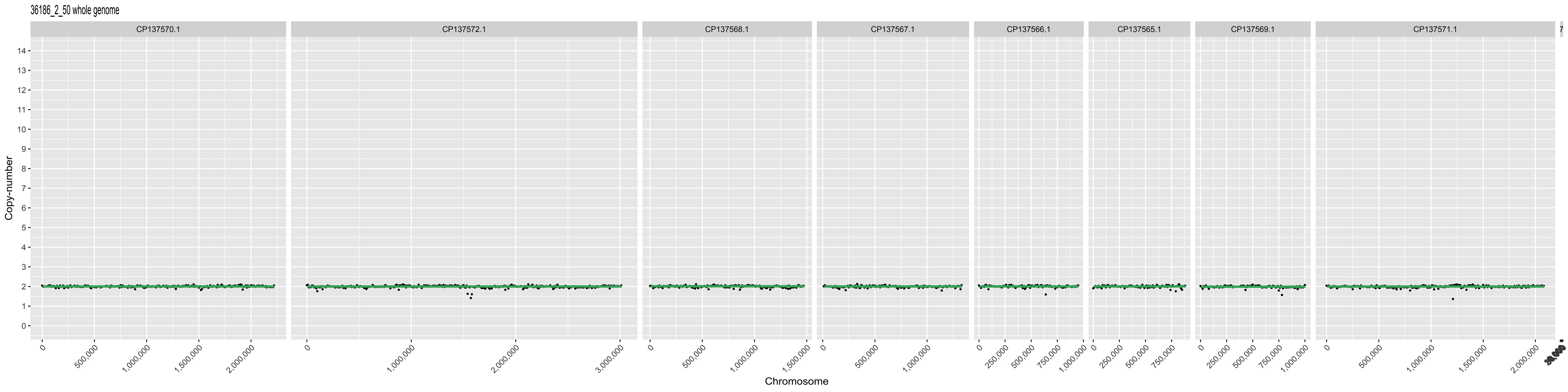
36186_2_50 whole genome
CP137570.1
CP137572.1
CP137568.1
CP137567.1
CP137566.1
CP137565.1
CP137569.1
CP137571.1
Copy-number
Chromosome

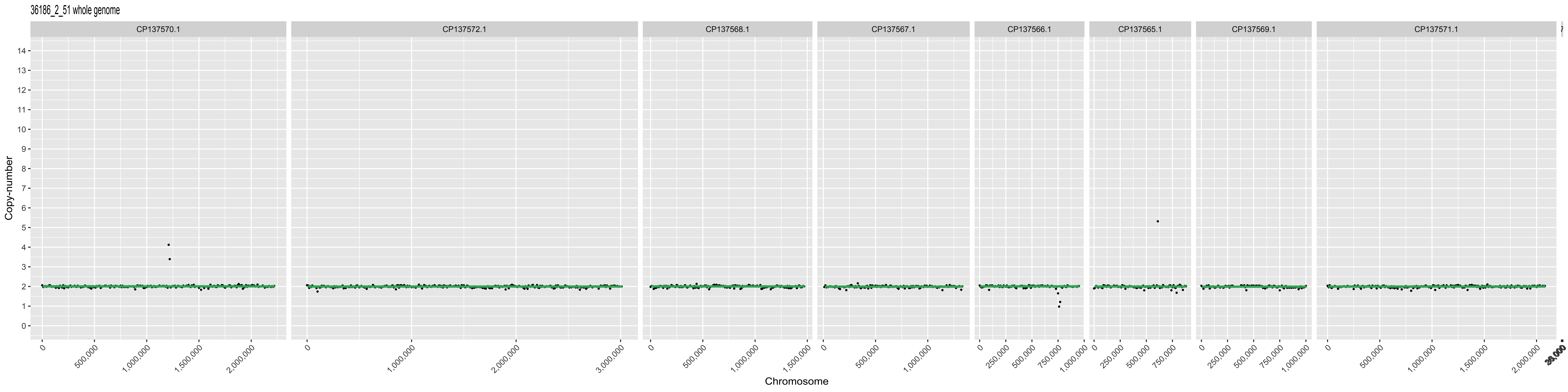

36186_2_51 whole genome
CP137570.1
CP137572.1
CP137568.1
CP137567.1
CP137566.1
CP137565.1
CP137569.1
CP137571.1
Copy-number
Chromosome
0
500,000
1,000,000
1,500,000
2,000,000
3,000,000
250,000
750,000

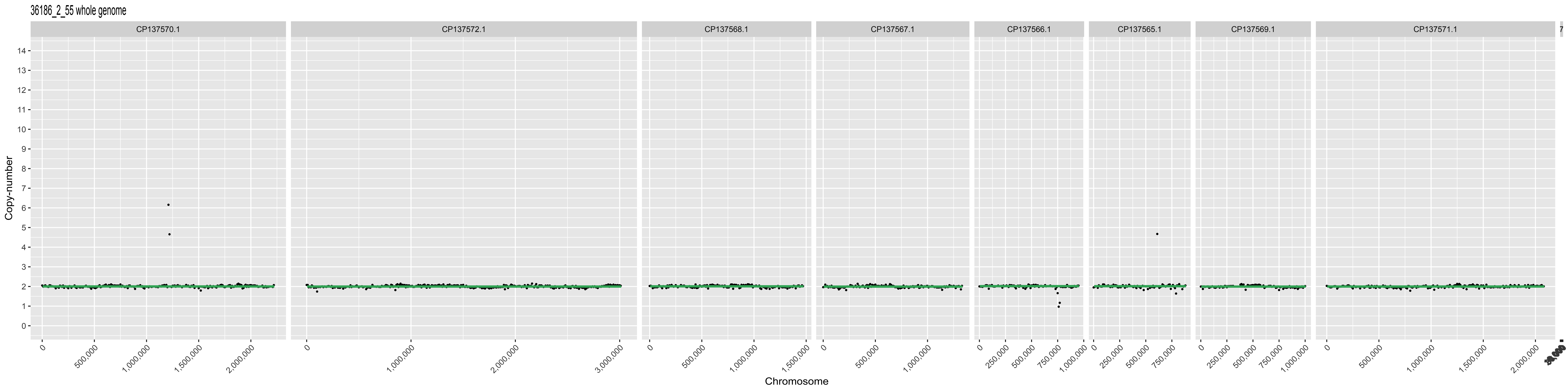
36186_2_55 whole genome
CP137570.1
CP137572.1
CP137568.1
CP137567.1
CP137566.1
CP137565.1
CP137569.1
CP137571.1
Copy-number
Chromosome

36186_2_57 whole genome

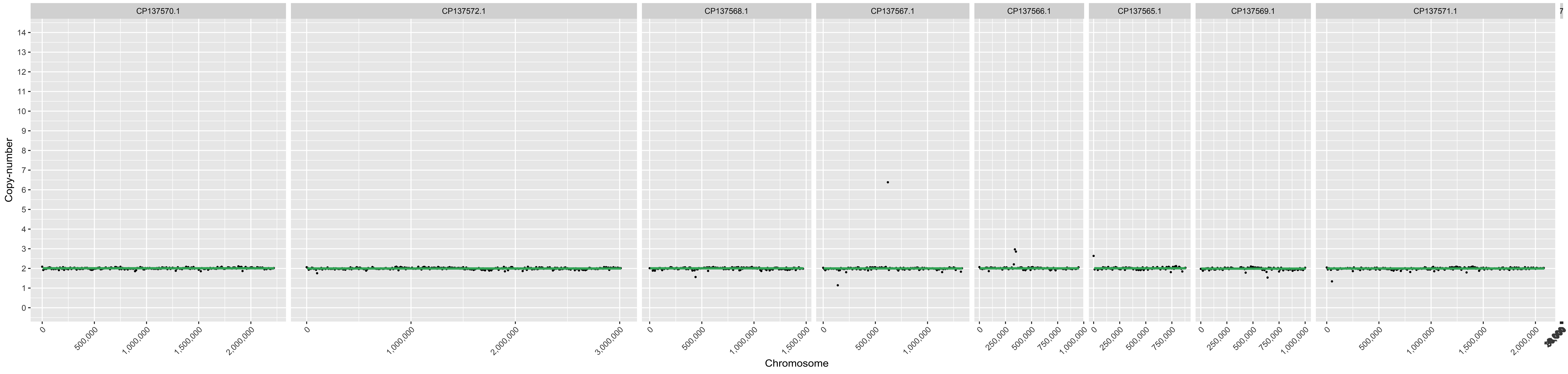

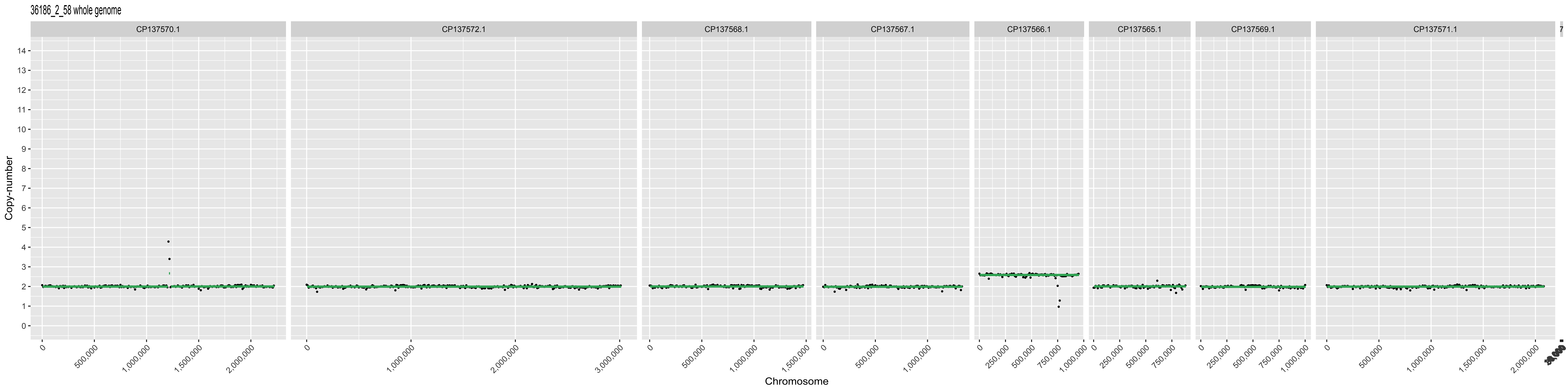

36186_2_58 whole genome
CP137570.1
CP137572.1
CP137568.1
CP137567.1
CP137566.1
CP137565.1
CP137569.1
CP137571.1
Copy-number
Chromosome

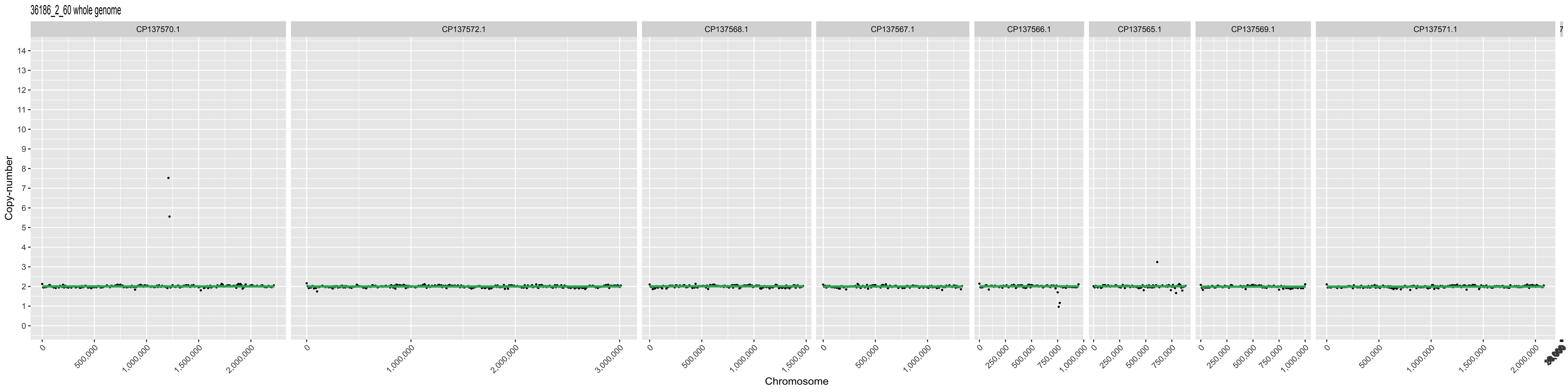
36186_2_60 whole genome
CP137570.1
CP137572.1
CP137568.1
CP137567.1
CP137566.1
CP137565.1
CP137569.1
CP137571.1
Copy-number
Chromosome

36186_2_62 whole genome

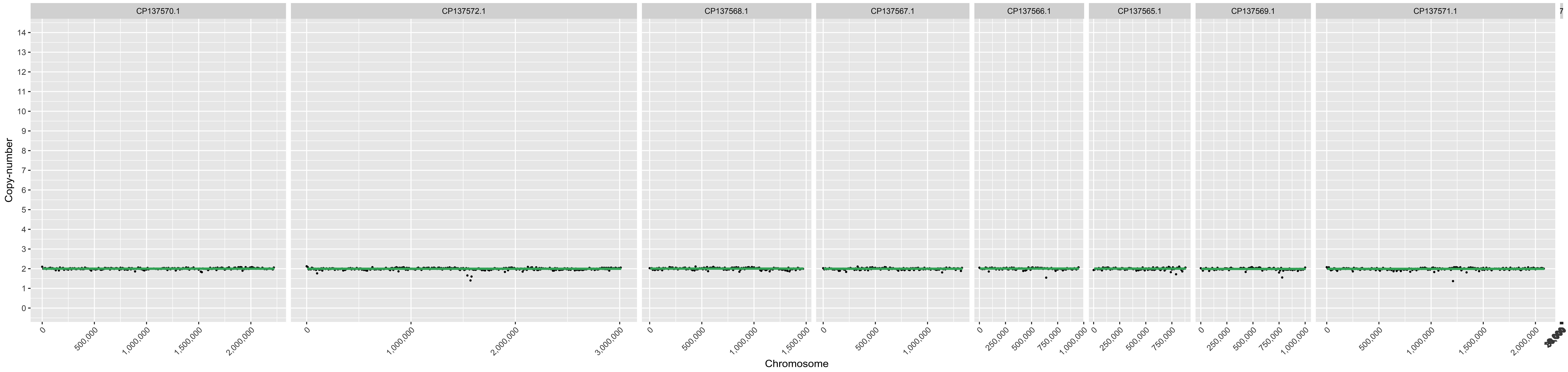

36186_2_63 whole genome

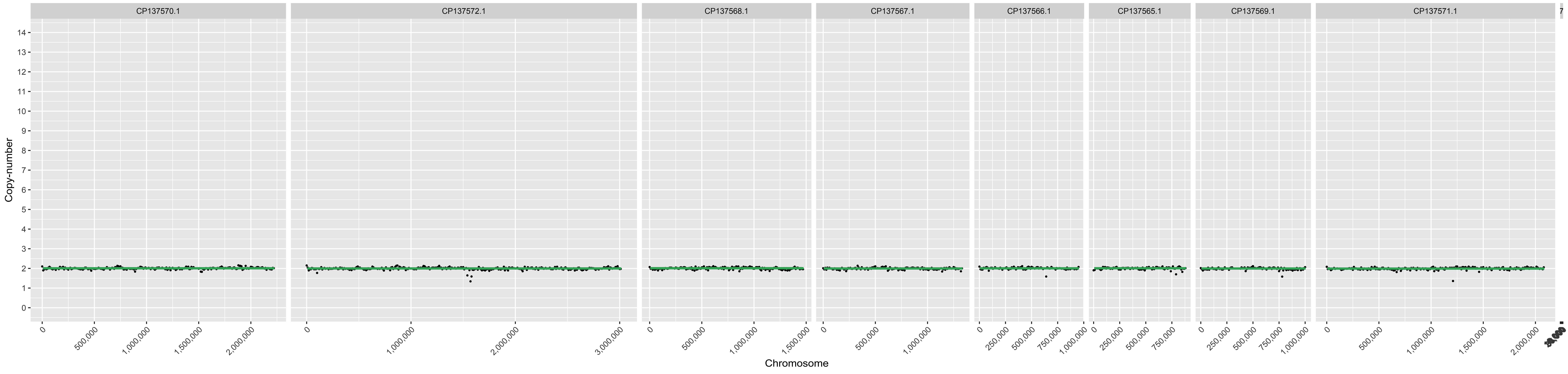

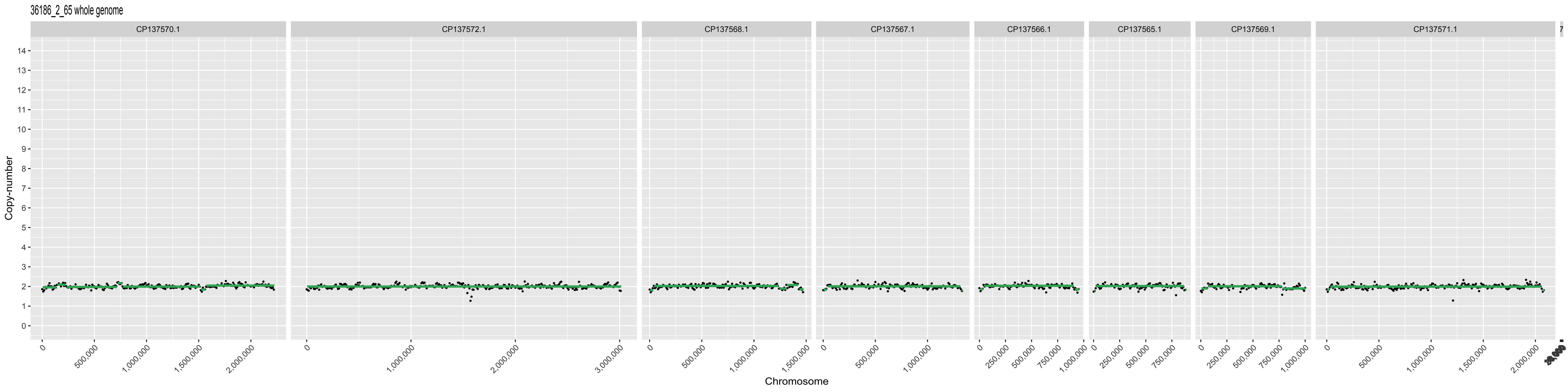
36186_2_65 whole genome
CP137570.1
CP137572.1
CP137568.1
CP137567.1
CP137566.1
CP137565.1
CP137569.1
CP137571.1
Copy-number
Chromosome

36186_2_66 whole genome

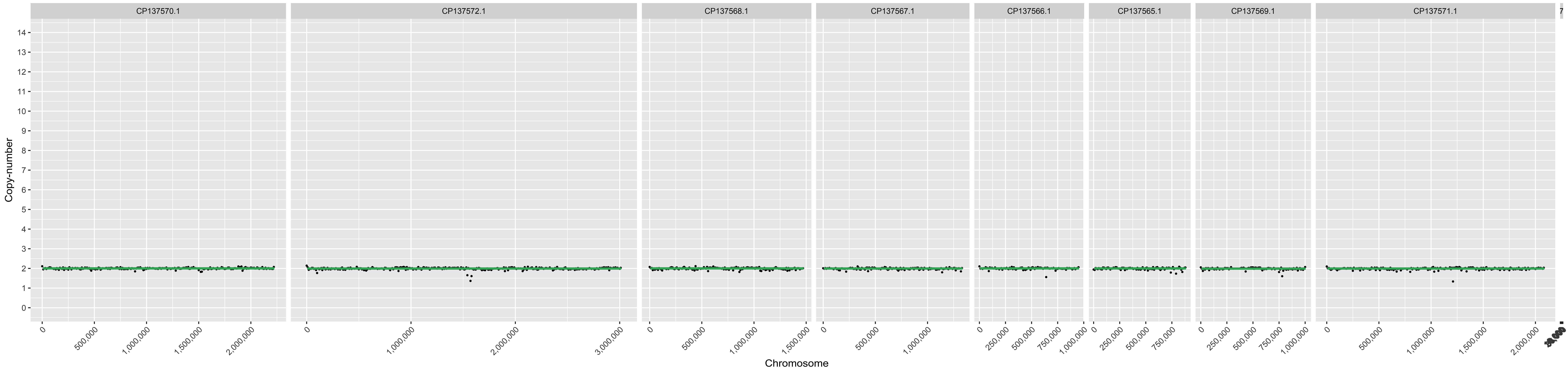

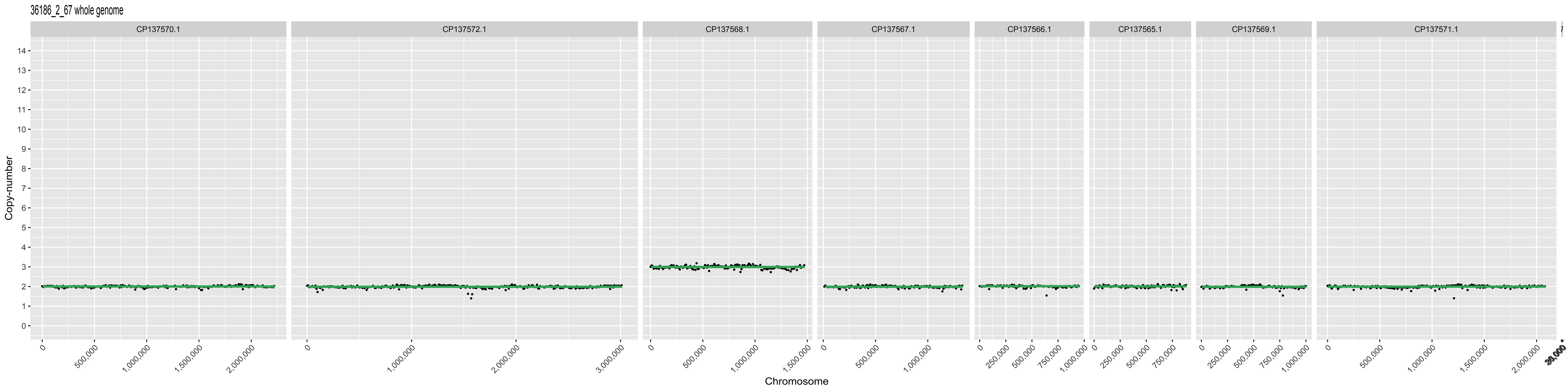

36186_2_67 whole genome
CP137570.1
CP137572.1
CP137568.1
CP137567.1
CP137566.1
CP137565.1
CP137569.1
CP137571.1
Copy-number
Chromosome

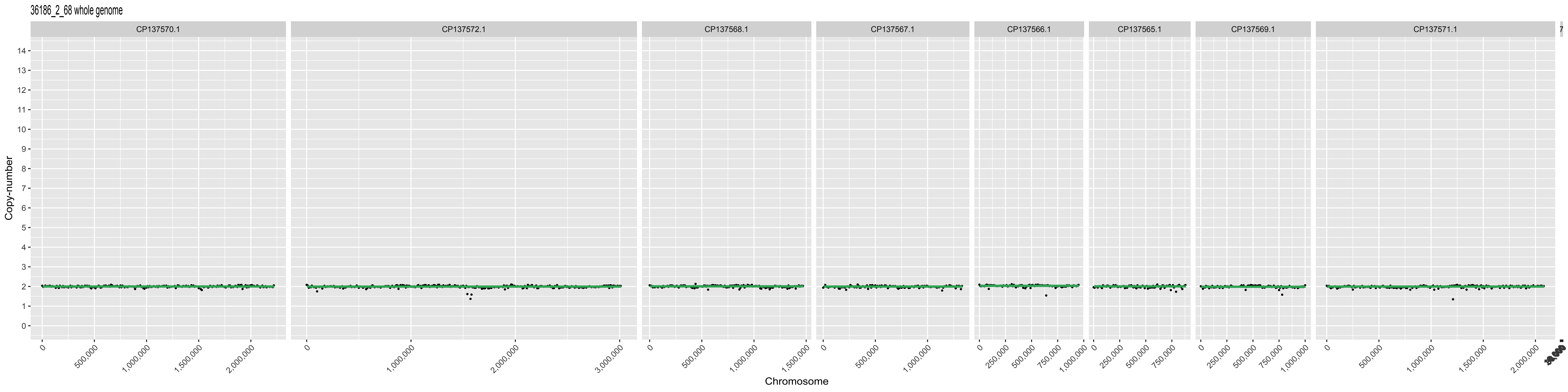

36186_2_68 whole genome
CP137570.1
CP137572.1
CP137568.1
CP137567.1
CP137566.1
CP137565.1
CP137569.1
CP137571.1
Copy-number
Chromosome

36186_2_69 whole genome

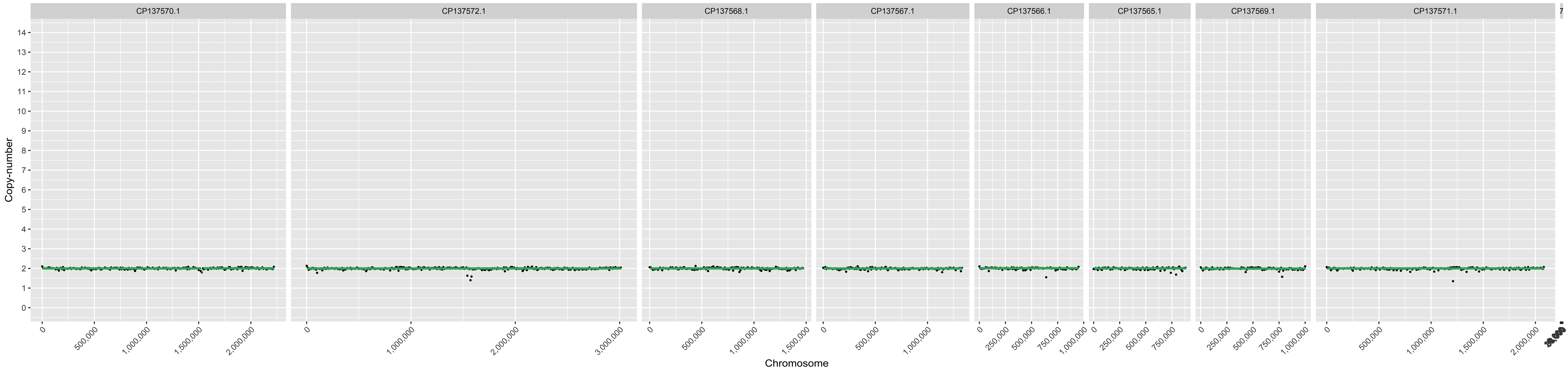

36186_2_70 whole genome

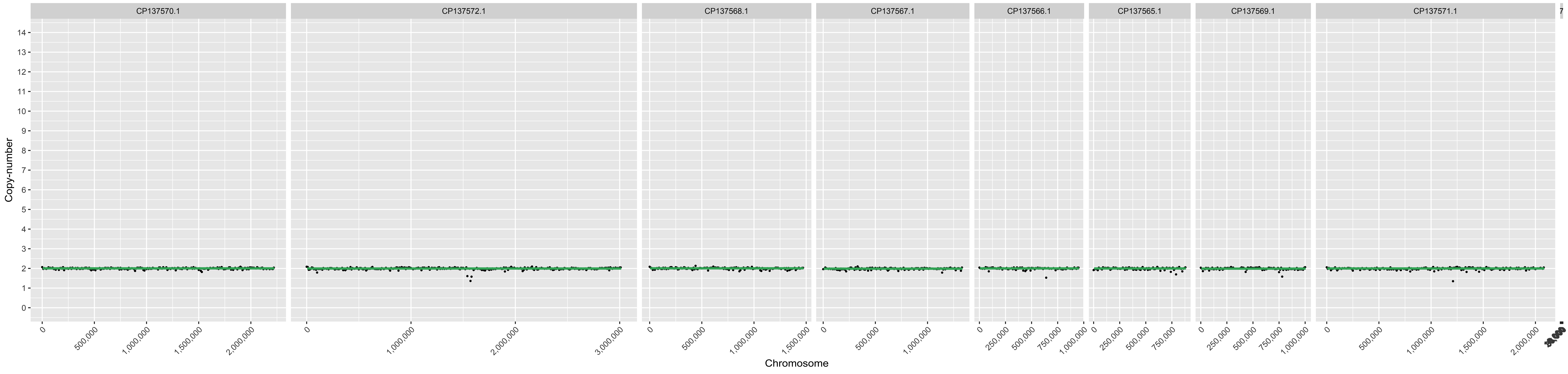

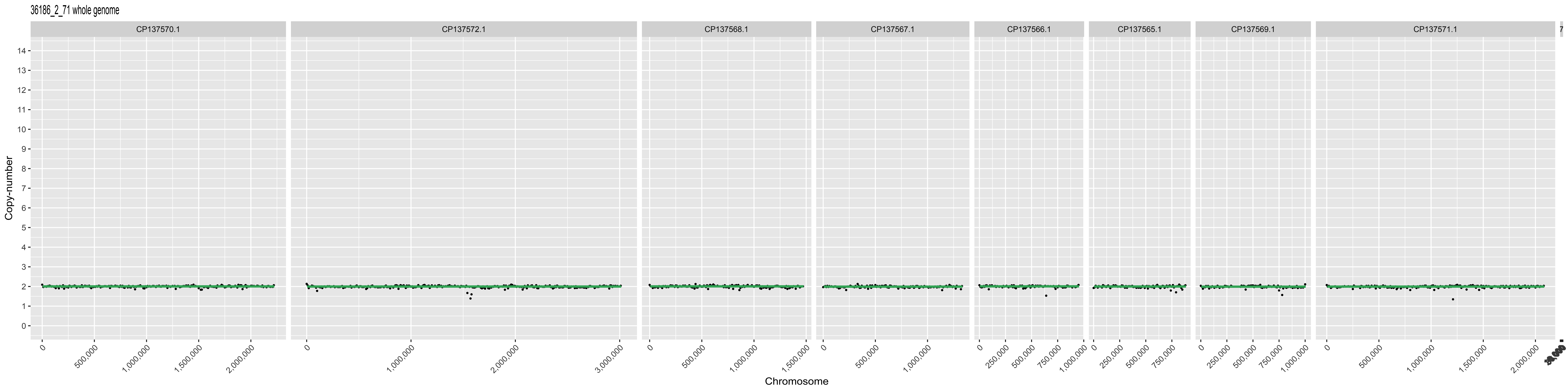

36186_2_71 whole genome
Copy-number
Chromosome
CP137570.1
CP137572.1
CP137568.1
CP137567.1
CP137566.1
CP137565.1
CP137569.1
CP137571.1
0
1
2
3
4
5
6
7
8
9
10
11
12
13
14
500,000
1,000,000
1,500,000
2,000,000
3,000,000
250,000
750,000

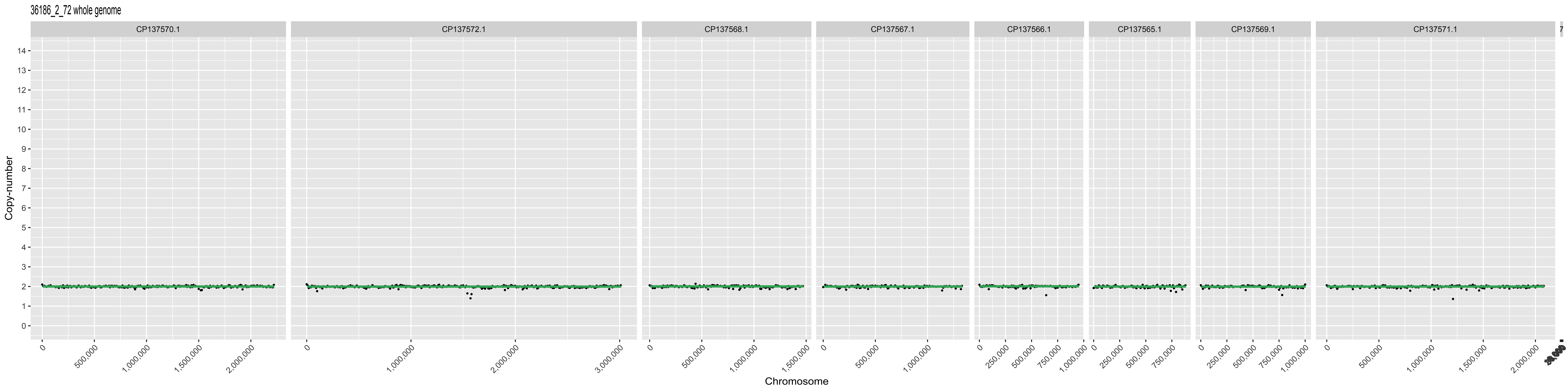

36186_2_72 whole genome
CP137570.1
CP137572.1
CP137568.1
CP137567.1
CP137566.1
CP137565.1
CP137569.1
CP137571.1
Copy-number
0
1
2
3
4
5
6
7
8
9
10
11
12
13
14
0
500,000
1,000,000
1,500,000
2,000,000
3,000,000
250,000
750,000
Chromosome

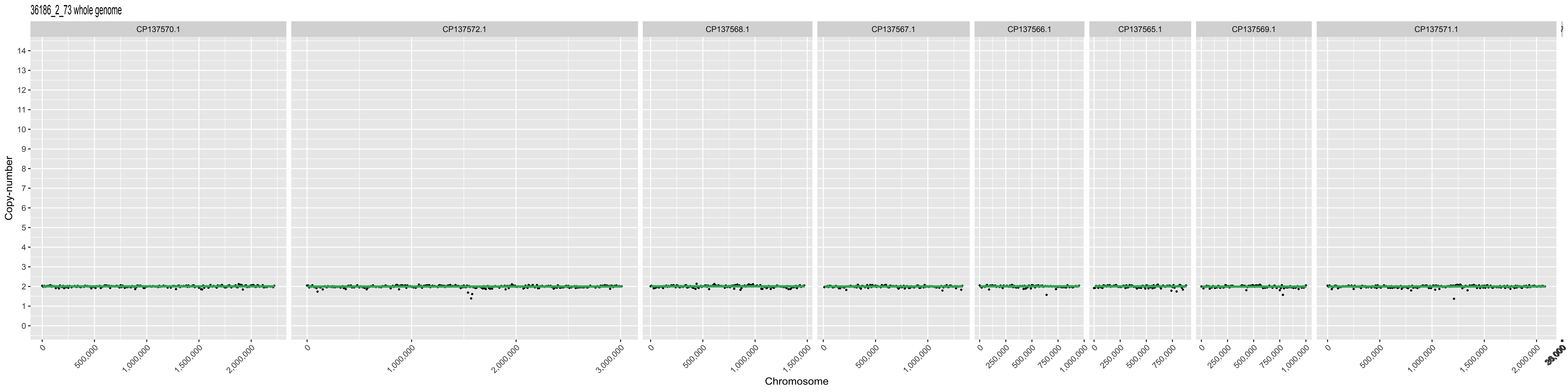
36186_2_73 whole genome
CP137570.1
CP137572.1
CP137568.1
CP137567.1
CP137566.1
CP137565.1
CP137569.1
CP137571.1
Copy-number
Chromosome
0
1
2
3
4
5
6
7
8
9
10
11
12
13
14
500,000
1,000,000
1,500,000
2,000,000
3,000,000
250,000
750,000

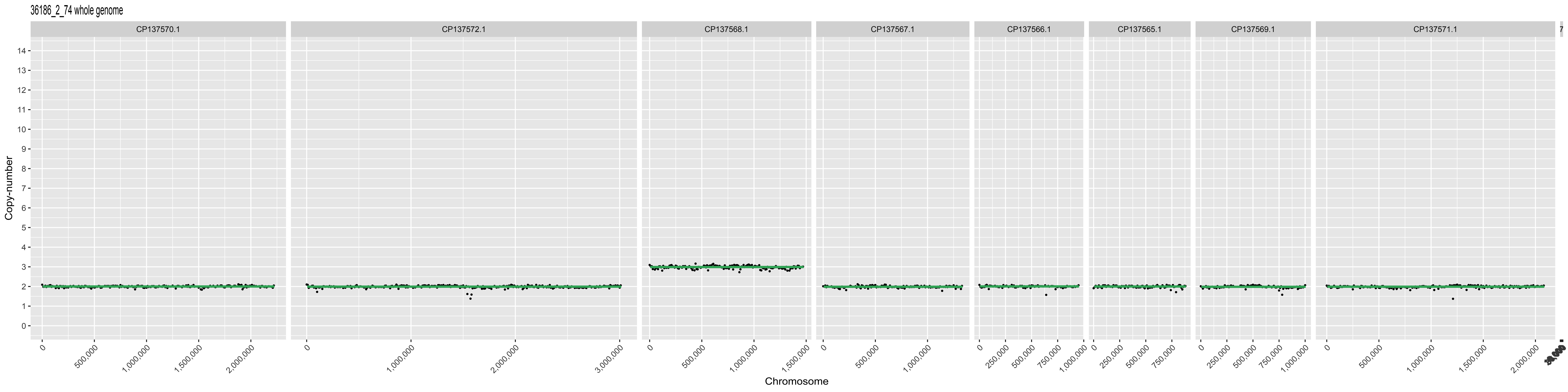
36186_2_74 whole genome
CP137570.1
CP137572.1
CP137568.1
CP137567.1
CP137566.1
CP137565.1
CP137569.1
CP137571.1
Copy-number
Chromosome
0
1
2
3
4
5
6
7
8
9
10
11
12
13
14
500,000
1,000,000
1,500,000
2,000,000
3,000,000
250,000
750,000

36186_2_75 whole genome

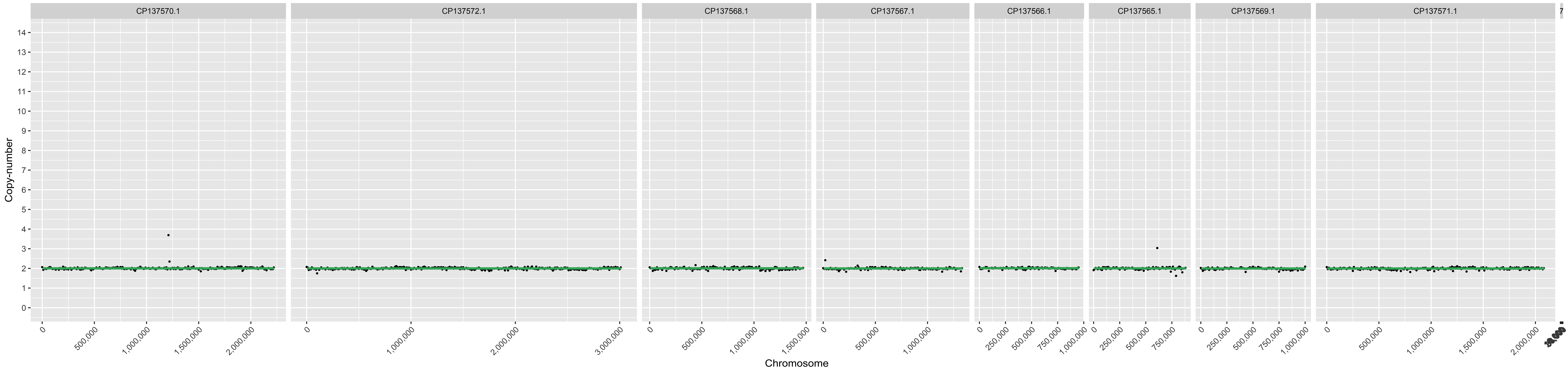

36186_2_76 whole genome

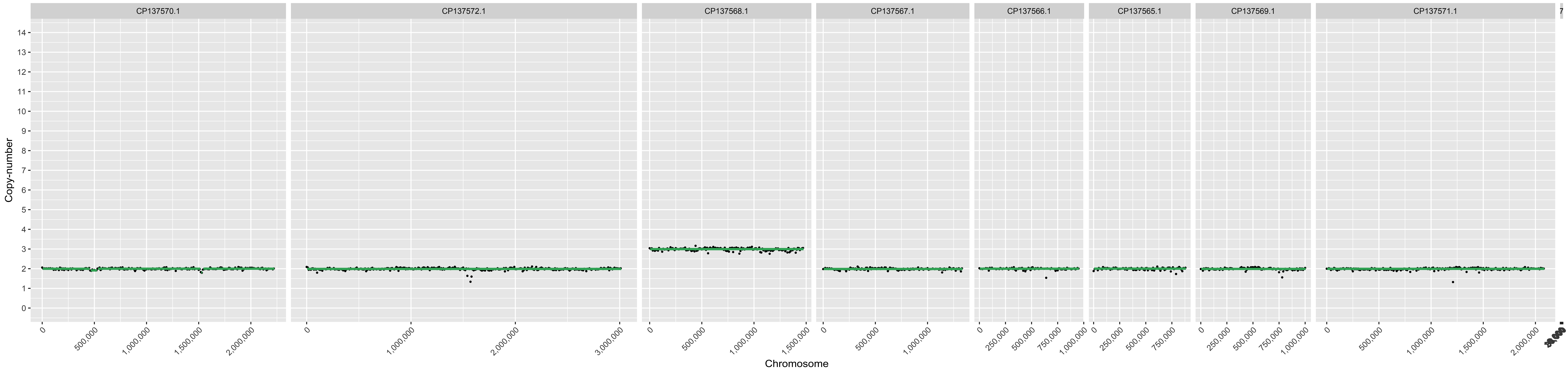

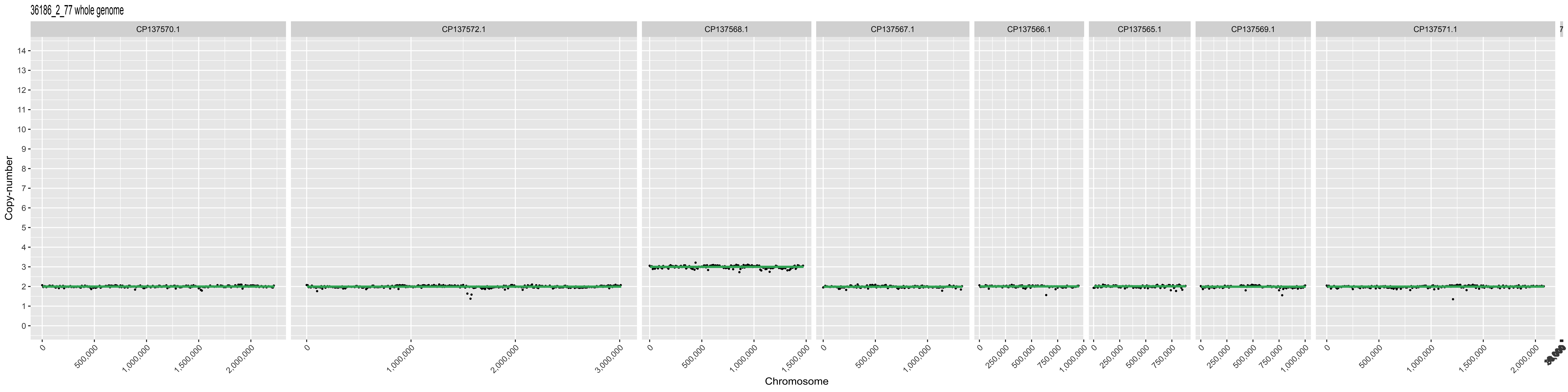

36186_2_77 whole genome
CP137570.1
CP137572.1
CP137568.1
CP137567.1
CP137566.1
CP137565.1
CP137569.1
CP137571.1
Copy-number
0
1
2
3
4
5
6
7
8
9
10
11
12
13
14
Chromosome
0
500,000
1,000,000
1,500,000
2,000,000
3,000,000
250,000
750,000

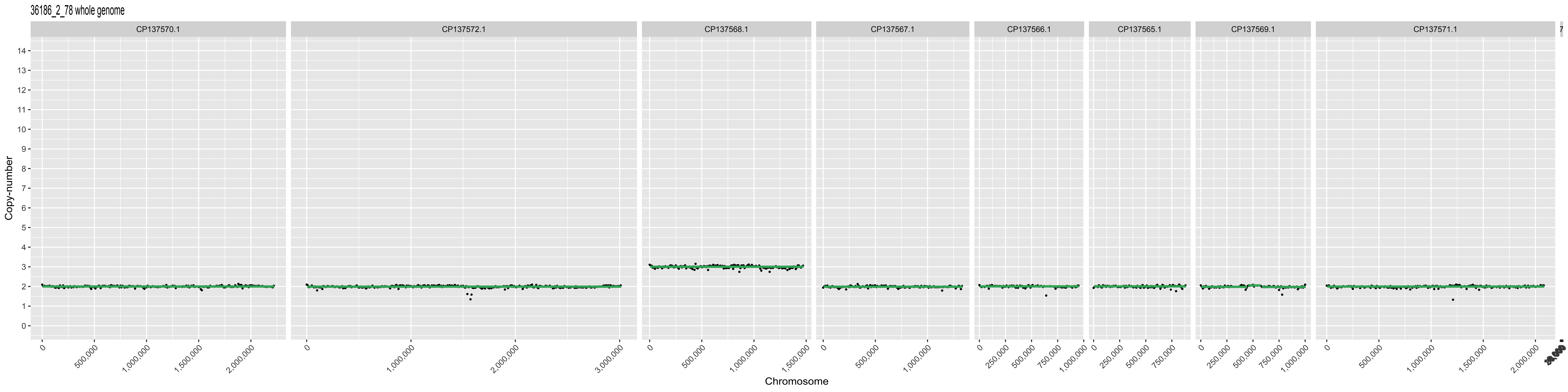
36186_2_78 whole genome
CP137570.1
CP137572.1
CP137568.1
CP137567.1
CP137566.1
CP137565.1
CP137569.1
CP137571.1
Copy-number
Chromosome

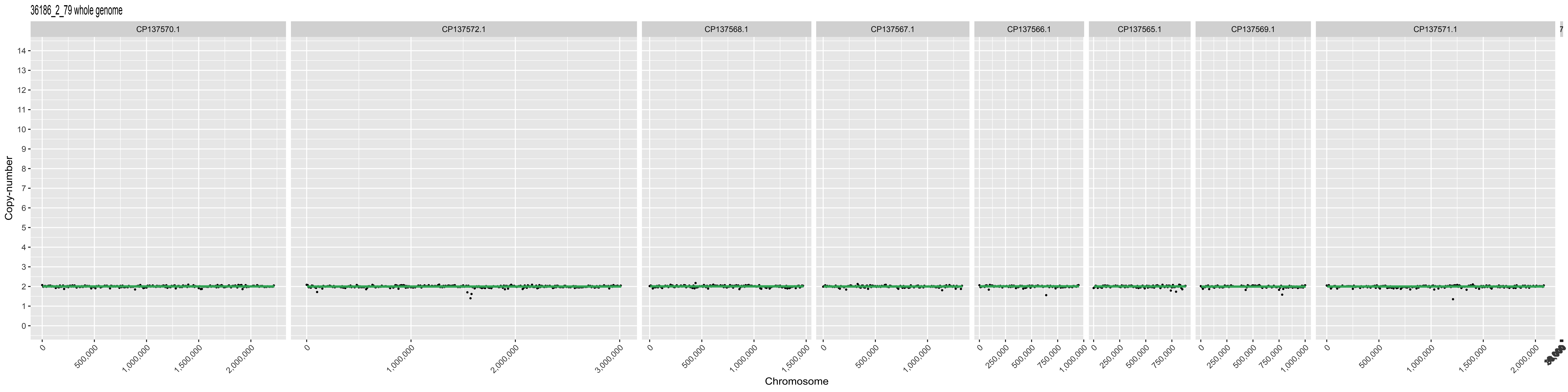

36186_2_79 whole genome
CP137570.1
CP137572.1
CP137568.1
CP137567.1
CP137566.1
CP137565.1
CP137569.1
CP137571.1
Copy-number
Chromosome

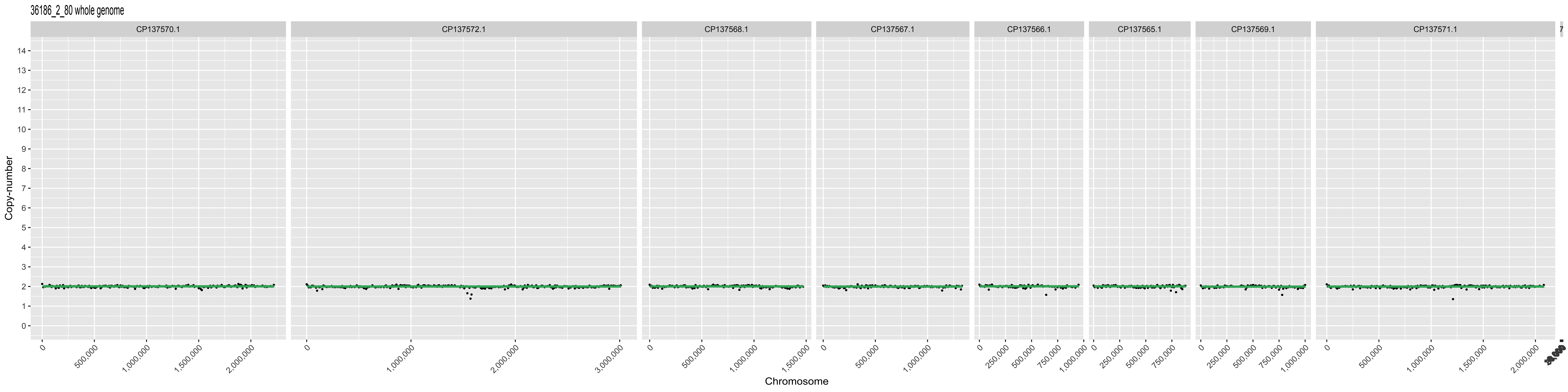
36186_2_80 whole genome
CP137570.1
CP137572.1
CP137568.1
CP137567.1
CP137566.1
CP137565.1
CP137569.1
CP137571.1
Copy-number
Chromosome
0
500,000
1,000,000
1,500,000
2,000,000
3,000,000
250,000
750,000

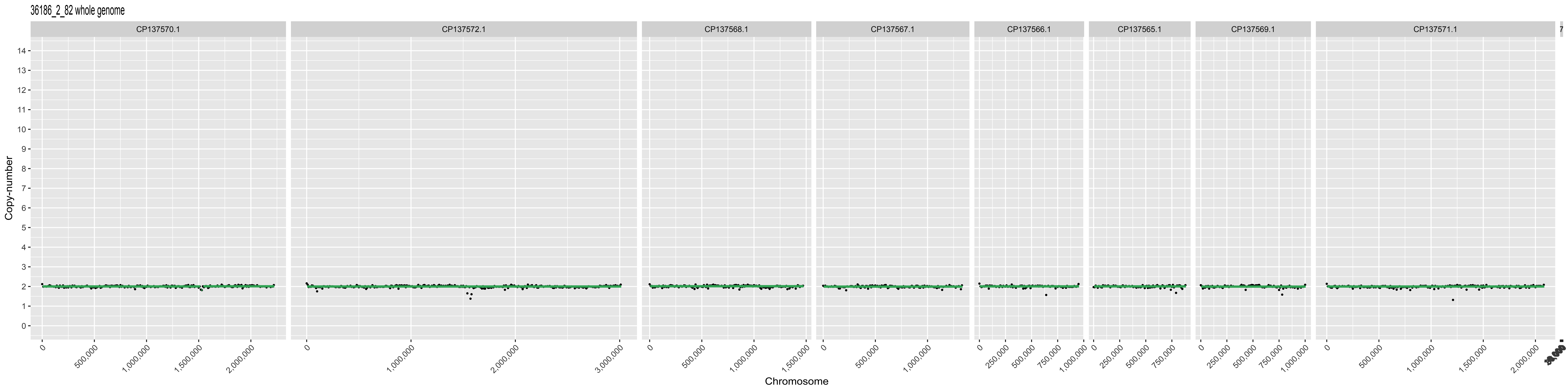

36186_2_82 whole genome
CP137570.1
CP137572.1
CP137568.1
CP137567.1
CP137566.1
CP137565.1
CP137569.1
CP137571.1
Copy-number
0
1
2
3
4
5
6
7
8
9
10
11
12
13
14
Chromosome
500,000
1,000,000
1,500,000
2,000,000
3,000,000
250,000
750,000

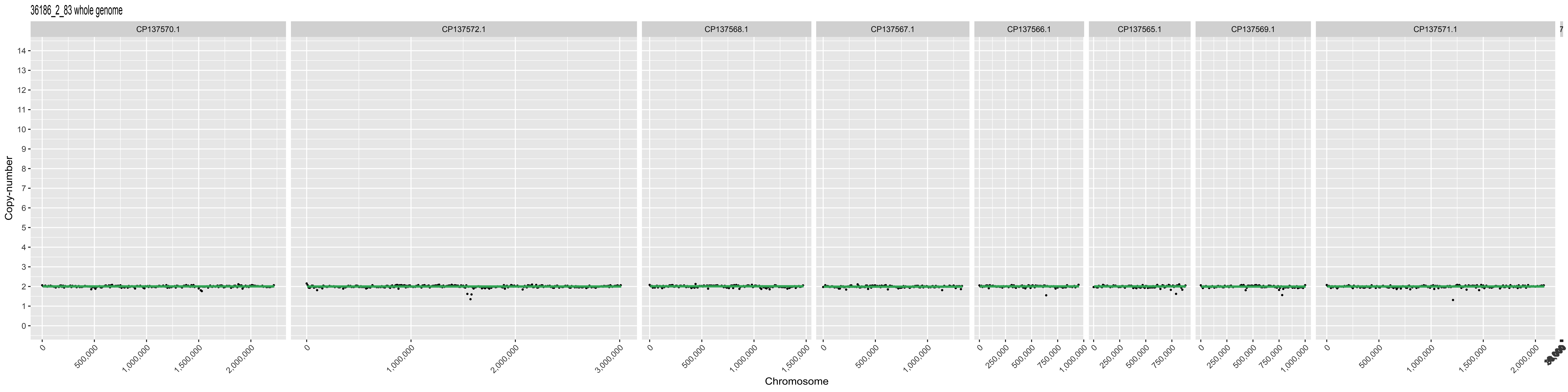

36186_2_83 whole genome
CP137570.1
CP137572.1
CP137568.1
CP137567.1
CP137566.1
CP137565.1
CP137569.1
CP137571.1
Copy-number
Chromosome

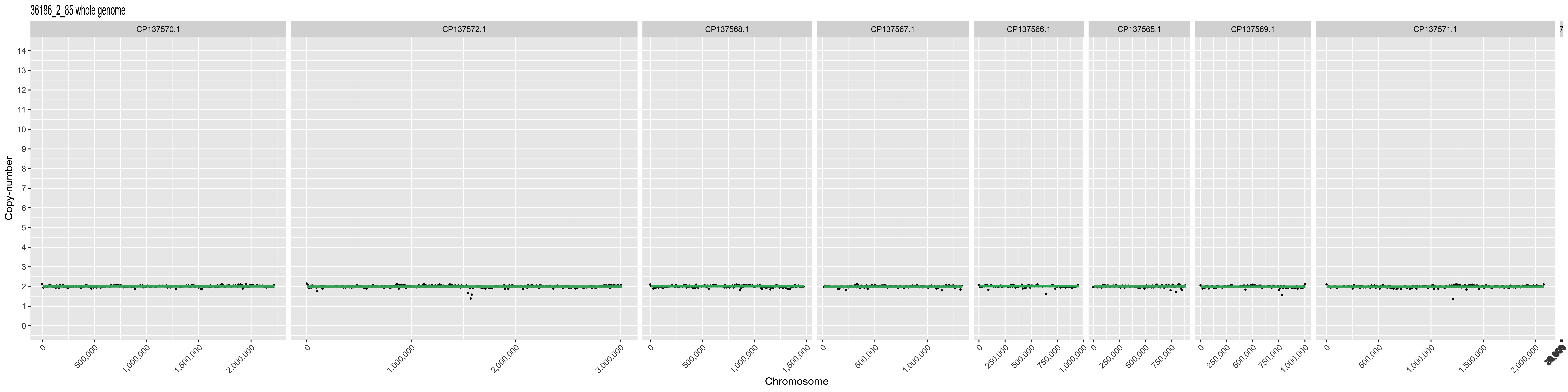

36186_2_85 whole genome
CP137570.1
CP137572.1
CP137568.1
CP137567.1
CP137566.1
CP137565.1
CP137569.1
CP137571.1
Copy-number
0
1
2
3
4
5
6
7
8
9
10
11
12
13
14
0
500,000
1,000,000
1,500,000
2,000,000
3,000,000
250,000
750,000
Chromosome

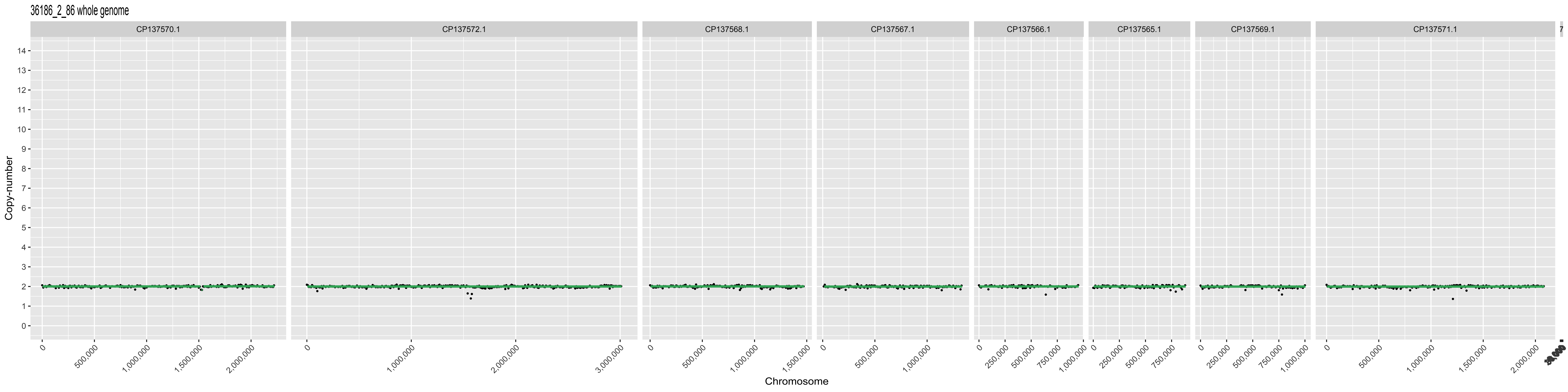

36186_2_86 whole genome
CP137570.1
CP137572.1
CP137568.1
CP137567.1
CP137566.1
CP137565.1
CP137569.1
CP137571.1
Copy-number
Chromosome

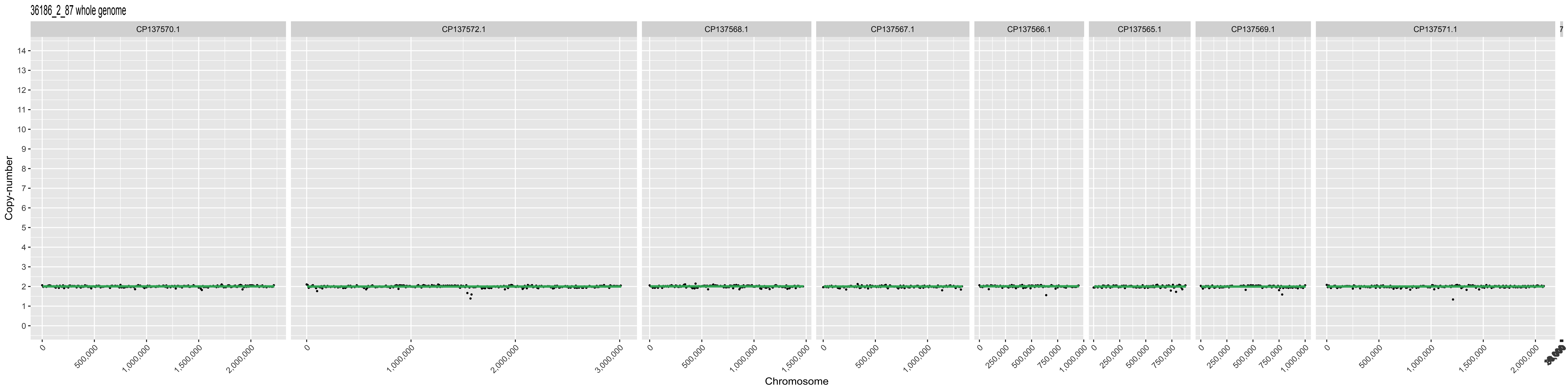
36186_2_87 whole genome
CP137570.1
CP137572.1
CP137568.1
CP137567.1
CP137566.1
CP137565.1
CP137569.1
CP137571.1
Copy-number
Chromosome

36186_2_89 whole genome

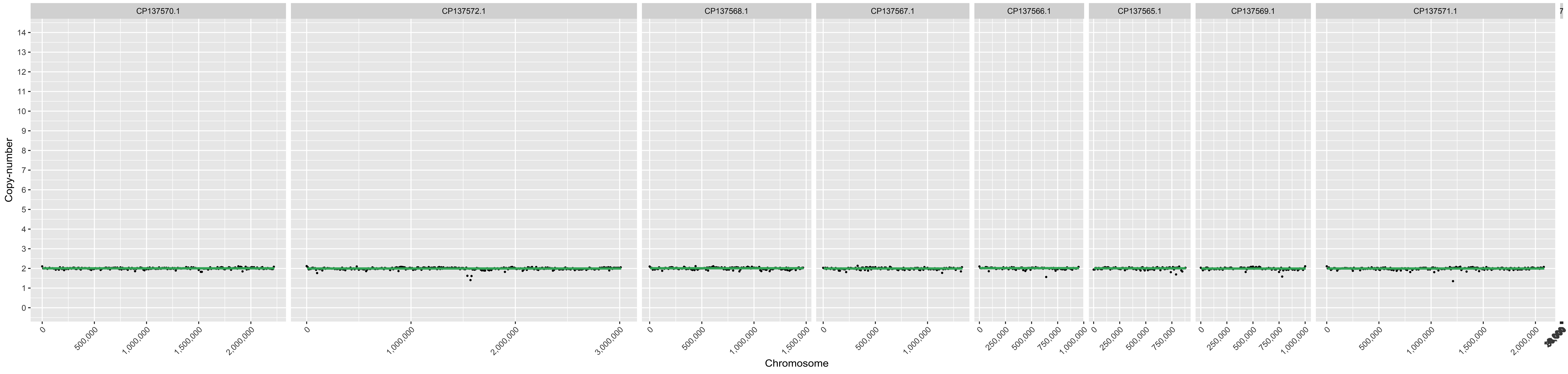

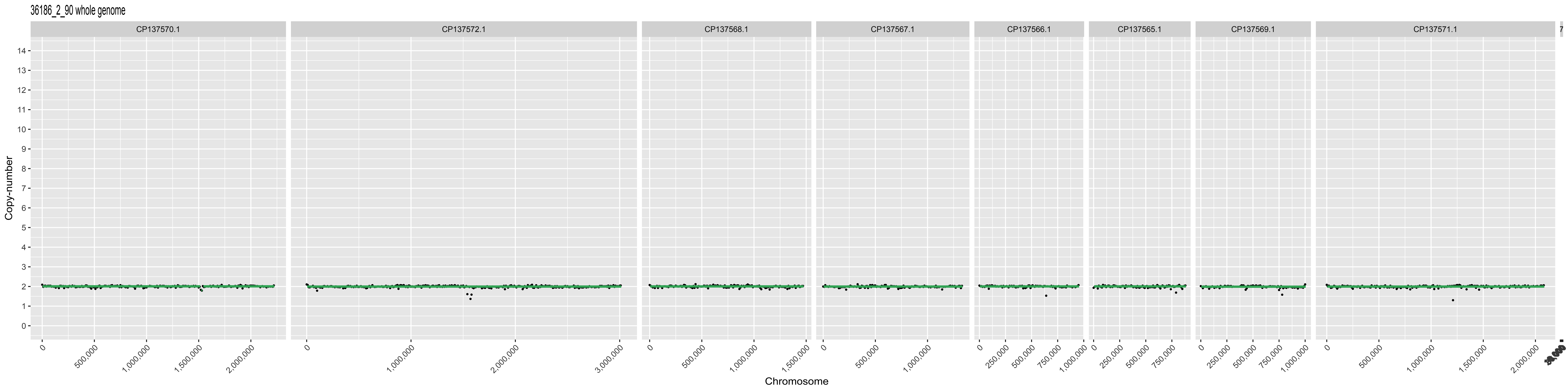
36186_2_90 whole genome
CP137570.1
CP137572.1
CP137568.1
CP137567.1
CP137566.1
CP137565.1
CP137569.1
CP137571.1
Copy-number
Chromosome

36186_2_91 whole genome

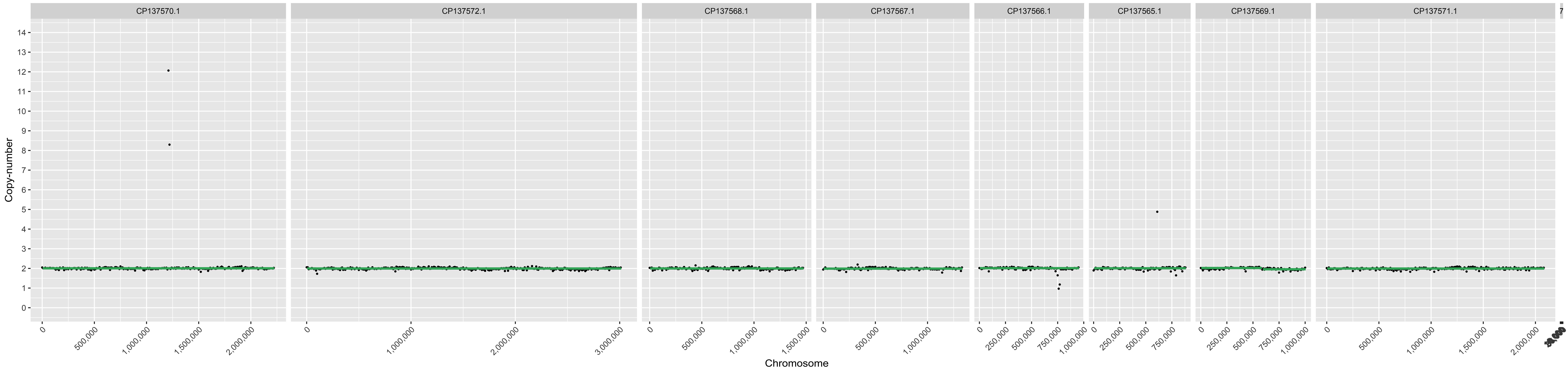

36186_2_92 whole genome

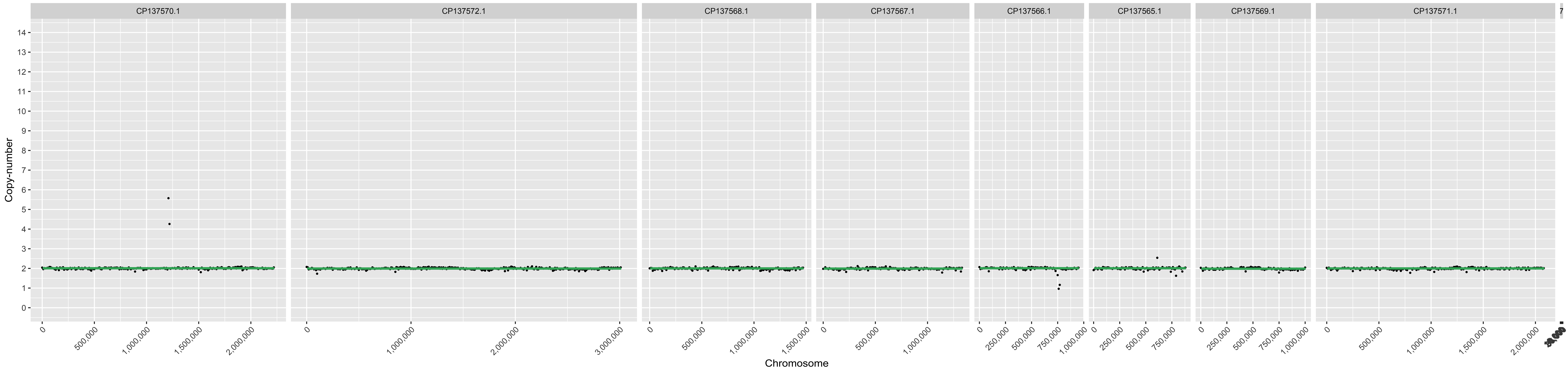

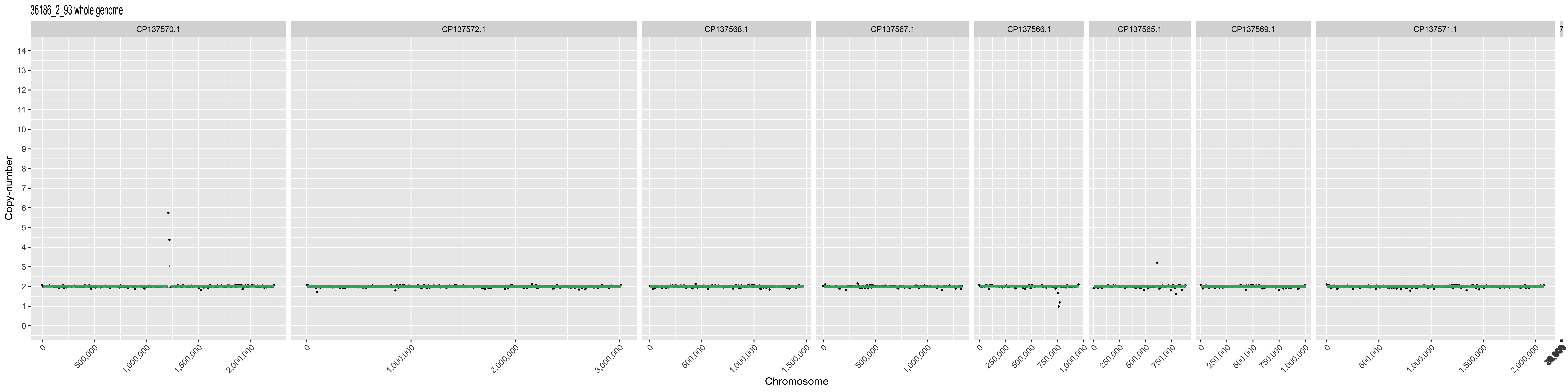
36186_2_93 whole genome
CP137570.1
CP137572.1
CP137568.1
CP137567.1
CP137566.1
CP137565.1
CP137569.1
CP137571.1
Copy-number
Chromosome
0
500,000
1,000,000
1,500,000
2,000,000
3,000,000
250,000
750,000

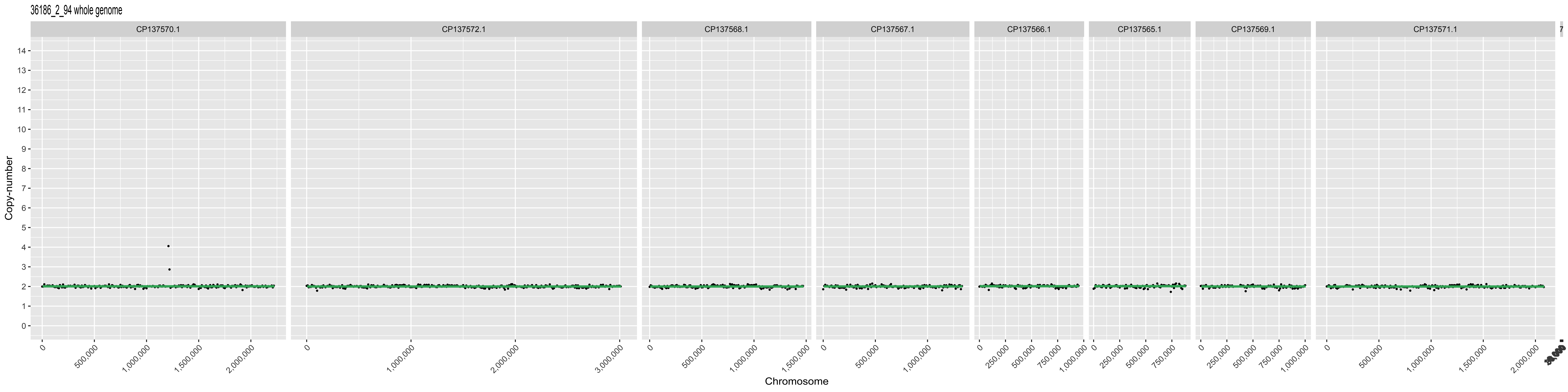

36186_2_94 whole genome
CP137570.1
CP137572.1
CP137568.1
CP137567.1
CP137566.1
CP137565.1
CP137569.1
CP137571.1
Copy-number
Chromosome

36186_2_95 whole genome

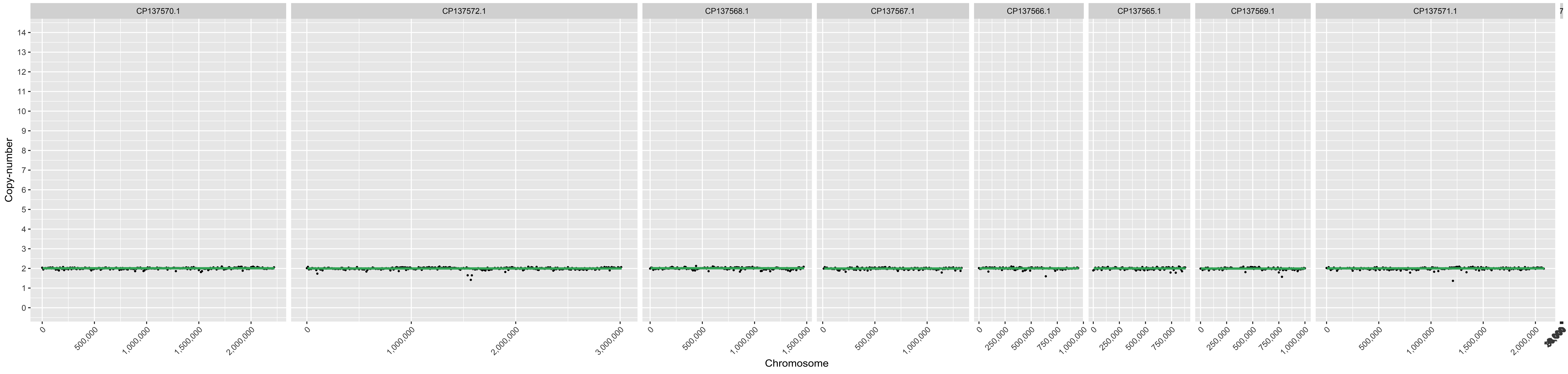

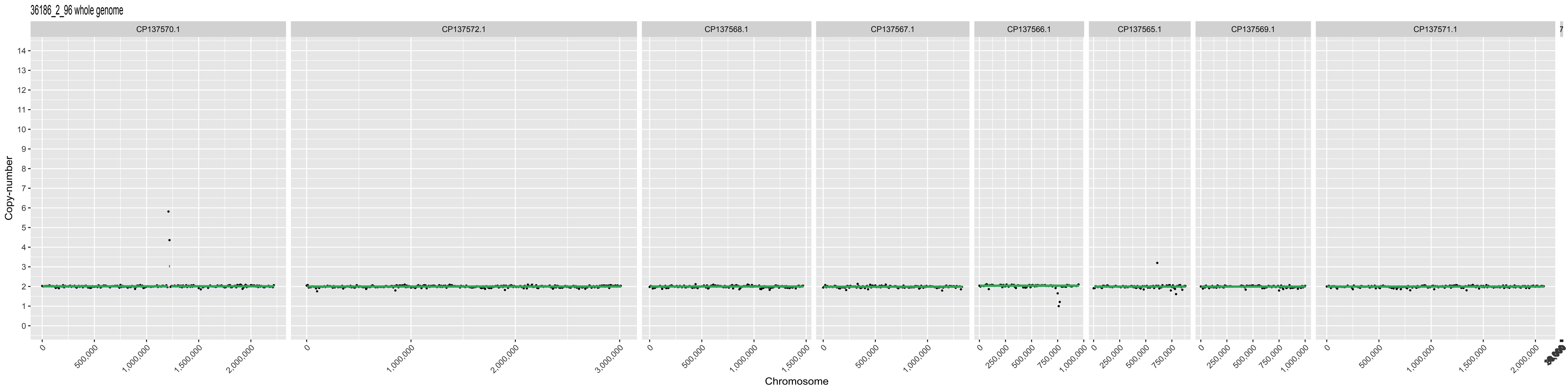

36186_2_96 whole genome
CP137570.1
CP137572.1
CP137568.1
CP137567.1
CP137566.1
CP137565.1
CP137569.1
CP137571.1
Copy-number
Chromosome
