# Supplementary material for: Genomic Characterization of Candida spp. Highlights a Persistent, Azole-Resistant C. parapsilosis Clone Circulating in a Tertiary Care Hospital During the First COVID-19 Wave
Source: Mycopathologia. 2026 Mar 16;191(2):44. doi: 10.1007/s11046-026-01070-9 (PMC12992396; doi:10.1007/s11046-026-01070-9)
Supplement: Supplementary file 5 — (PDF 85 KB) [file 11046_2026_1070_MOESM5_ESM.pdf]

Bergin, S. A., Zhao, F., Ryan, A. P., Müller, C. A., Nieduszynski, C. A., Zhai, B., Rolling, T., Hohl, T. M., Morio, F., Scully, J., Wolfe, K. H., & Butler, G. (2022). Systematic Analysis of Copy Number Variations in the Pathogenic Yeast Candida parapsilosis Identifies a Gene Amplification in *RTA3* That is Associated with Drug Resistance. *mBio*, *13*(5), e0177722. https://doi.org/10.1128/mbio.01777-22

Zhai, B., Liao, C., Jaggavarapu, S., Tang, Y., Rolling, T., Ning, Y., Sun, T., Bergin, S. A., Gjonbalaj, M., Miranda, E., Babady, N. E., Bader, O., Taur, Y., Butler, G., Zhang, L., Xavier, J. B., Weiss, D. S., & Hohl, T. M. (2024). Antifungal heteroresistance causes prophylaxis failure and facilitates breakthrough Candida parapsilosis infections. *Nature medicine*, 10.1038/s41591-024-03183-4. Advance online publication. https://doi.org/10.1038/s41591-024-03183-4

Guinea, J., Mezquita, S., Gómez, A., Padilla, B., Zamora, E., Sánchez-Luna, M., Sánchez-Carrillo, C., Muñoz, P., & Escribano, P. (2021). Whole genome sequencing confirms Candida albicans and Candida parapsilosis microsatellite sporadic and persistent clones causing outbreaks of candidemia in neonates. *Medical mycology*, *60*(1), myab068. https://doi.org/10.1093/mmy/myab068

Phillip Brassington, Frank-Rainer Klefisch, Barbara Graf, Roland Pfüller, Oliver Kurzai, Grit Walther, View ORCID ProfileAmelia E. Barber (2024). Genomic reconstruction of an azole-resistant Candida parapsilosis outbreak and the creation of a multilocus sequence typing scheme. https://doi.org/10.1101/2024.02.22.24302918

Asadzadeh M, Dashti M, Ahmad S, Alfouzan W, Alameer A. 2021. Whole-Genome and Targeted-Amplicon Sequencing of Fluconazole-Susceptible and -Resistant Candida parapsilosis Isolates from Kuwait Reveals a Previously Undescribed N1132D Polymorphism in *CDR1*. Antimicrob Agents Chemother 65:10.1128/aac.01633-20.
https://doi.org/10.1128/aac.01633-20

Yamin, D., Wan Juhari, W. K., Hanis Zainal Abidin, N. W., Mat-Sharani, S., & Harun, A. (2022). Draft Genome Sequence of Clinical Isolate USM026 of the Pathogenic Yeast Candida parapsilosis. *Microbiology resource announcements*, *11*(11), e0083922. https://doi.org/10.1128/mra.00839-22

De Luca, D. G., Alexander, D. C., Dingle, T. C., Dufresne, P. J., Hoang, L. M., Kus, J. V., Schwartz, I. S., Mulvey, M. R., & Bharat, A. (2022). Four genomic clades of Candida auris identified in Canada, 2012-2019. *Medical mycology*, *60*(1), myab079. https://doi.org/10.1093/mmy/myab079

Kim, T. Y., Huh, H. J., Lee, G. Y., Choi, M. J., Yu, H. J., Cho, S. Y., Chang, Y. S., Kim, Y. J., Shin, J. H., & Lee, N. Y. (2022). Evolution of Fluconazole Resistance Mechanisms and Clonal Types of *Candida parapsilosis* Isolates from a Tertiary Care Hospital in South Korea. *Antimicrobial agents and chemotherapy*, *66*(11), e0088922. https://doi.org/10.1128/aac.00889-22

Pinto, M., Borges, V., Nascimento, M., Martins, F., Pessanha, M. A., Faria, I., Rodrigues, J., Matias, R., Gomes, J. P., & Jordao, L. (2022). Insights on catheter-related bloodstream infections: a prospective observational study on the catheter colonization and multidrug resistance. *The Journal of hospital infection*, *123*, 43–51. https://doi.org/10.1016/j.jhin.2022.01.025

Daneshnia, F., Floyd, D. J., Ryan, A. P., Ghahfarokhy, P. M., Ebadati, A., Jusuf, S., Munoz, J., Jeffries, N. E., Elizabeth Yvanovich, E., Apostolopoulou, A., Perry, A. M., Lass-Flörl, C., Birinci, A., Hilmioğlu-Polat, S., Ilkit, M., Butler, G., Nobile, C. J., Arastehfar, A., & Mansour, M. K. (2024). Evaluation of outbreak persistence caused by multidrug-resistant and echinocandin-resistant *Candida parapsilosis* using multidimensional experimental and epidemiological approaches. *Emerging microbes & infections*, *13*(1), 2322655. https://doi.org/10.1080/22221751.2024.2322655

Daneshnia, F., Hilmioğlu-Polat, S., Ilkit, M., Fuentes, D., Lombardi, L., Binder, U., Scheler, J., Hagen, F., Mansour, M. K., Butler, G., Lass-Flörl, C., Gabaldon, T., & Arastehfar, A. (2023). Whole-genome sequencing confirms a persistent candidaemia clonal outbreak due to multidrug-resistant Candida parapsilosis. *The Journal of antimicrobial chemotherapy*, *78*(6), 1488–1494. https://doi.org/10.1093/jac/dkad112

Misas, E., Witt, L. S., Farley, M. M., Thomas, S., Jenkins, E. N., Gade, L., Peterson, J. G., Mesa Restrepo, A., Fridkin, S., Lockhart, S. R., Chow, N. A., & Lyman, M. (2024). Molecular and Epidemiological Investigation of Fluconazole-resistant *Candida parapsilosis*-Georgia, United States, 2021. *Open forum infectious diseases*, *11*(6), ofae264. https://doi.org/10.1093/ofid/ofae264

Ola, M., O'Brien, C. E., Coughlan, A. Y., Ma, Q., Donovan, P. D., Wolfe, K. H., & Butler, G. (2020). Polymorphic centromere locations in the pathogenic yeast *Candida parapsilosis*. *Genome research*, *30*(5), 684–696. https://doi.org/10.1101/gr.257816.119

Chew, K. L., Octavia, S., Jureen, R., Lin, R. T. P., & Teo, J. W. P. (2021). Targeted amplification and MinION nanopore sequencing of key azole and echinocandin resistance determinants of clinically relevant Candida spp. from blood culture bottles. *Letters in applied microbiology*, *73*(3), 286–293. https://doi.org/10.1111/lam.13516

NCBI Bioproject (2024) Draft genome sequence of Candida parapsilosis NCYC 4418. Bioproject ID: PRJNA1124071. Available at: https://www.ncbi.nlm.nih.gov/bioproject/PRJNA1124071

Ropars, J., Maufrais, C., Diogo, D., Marcet-Houben, M., Perin, A., Sertour, N., Mosca, K., Permal, E., Laval, G., Bouchier, C., Ma, L., Schwartz, K., Voelz, K., May, R. C., Poulain, J., Battail, C., Wincker, P., Borman, A. M., Chowdhary, A., Fan, S., … d'Enfert, C. (2018). Gene flow contributes to diversification of the major fungal pathogen Candida albicans. *Nature communications*, *9*(1), 2253. https://doi.org/10.1038/s41467-018-04787-4
